# Benzamides of 2-(aminophenyl)benzimidazoles and 2-(aminophenyl)indoles as Anticancer Scaffolds: Synthesis, In Silico and In Vitro Evaluation

**DOI:** 10.3390/ph19091485

**Published:** 2026-09-17

**Authors:** Adil Saeed, Humaira Nadeem, Fouzia Perveen Malik, Rehan Zafar Paracha, Sehrosh Naz Khan

**Affiliations:** 1 Riphah Institute of Pharmaceutical Sciences, Riphah International University, Islamabad 44000, Pakistan; humaira.nadeem@riphah.edu.pk (H.N.); rehan.paracha@riphah.edu.pk (R.Z.P.); 2 Materials Modelling Lab, School of Interdisciplinary Engineering & Sciences (SINES), NUST, Islamabad 44000, Pakistan; fouzia@sines.nust.edu.pk; 3 Panjwani Center for Molecular Medicine and Drug Research, International Center for Chemical & Biological Sciences (ICCBS), University of Karachi, Karachi 75270, Pakistan; ana_sehrosh@live.com

**Keywords:** 2-phenylbenzimidazole, 2-phenylindole, kinase, MD Simulation, cytotoxicity

## Abstract

**Background/Objectives**: Indoles and benzimidazoles are important nitrogen-containing heterocyclic scaffolds and are a cornerstone of synthetic drugs. In this study, benzamides of 2-aminophenylindoles and 2-aminophenylbenzimidazoles were synthesized and evaluated for their potential. Further, they were also subjected to computational studies for their ADMET profiling and binding with target proteins. **Methods**: The synthesized compounds were characterized by ATR-FTIR, 1H NMR, 13C NMR and electrospray ionization mass spectroscopy (ESI-MS). They were screened for their in vitro antibacterial activity by the Microplate Alamar Blue Assay (MABA) and for their anticancer potential by the MTT assay against the HeLa, PC3 and 3T3 cell lines. Further, the compounds were assessed for their ADMET profiles by the Deep-PK platform, and binding with selected kinases was assessed by AutoDock Vina v1.2.7, followed by MD simulations in GROMACS. Density functional theory (DFT) calculations were also performed to investigate the electronic properties of the synthesized compounds. **Results**: All synthesized compounds were inactive in antibacterial assays and mildly active against cancer cells. N-[4-(1H-benzimidazol-2-yl-phenyl]benzamide (4-APB-B) exhibited good activity against the HeLa cell line (IC50: 5.48 ± 0.01 µM) while showing very low cytotoxicity against the 3T3 cell line (selectivity index: 10.7), against which doxorubicin was highly active, indicating selectivity towards specific cancer cells. Docking studies indicated favorable binding affinities towards the selected kinase targets. Further, ligands with the best binding affinities in docking studies were subjected to MD simulations of 100 ns, and DFT studies were also performed to assess the electronic properties of the synthesized compounds. **Conclusions**: Our study provides a pathway for the synthesis of 2-phenylbenzimidazoles and 2-phenylindoles and demonstrates that the synthetic compound 4-APB-B possesses remarkable selective cytotoxic activity and can serve as a lead molecule for further development into a successful anticancer agent.

## 1. Introduction

Benzimidazoles and indoles are nitrogen-containing bicyclic aromatic scaffolds with great medicinal importance and are part of the structures of multiple approved drugs. These heterocyclic molecules also resemble multiple biomolecules. For example, tryptophan, an essential amino acid, contains an indole nucleus. Given their structural similarities with adenine, their derivatives have the potential to bind to ATP binding sites, which can result in the inhibition of vital cellular functions, leading to cell death [1,2,3,4].

Benzimidazole and indole derivatives have been frequently reported as potent inhibitors of bacterial DNA gyrase and Topoisomerase IV, resulting in activity against both Gram-positive and Gram-negative pathogens. Several marketed antibiotics, like daptomycin and mytomycin C, have indole scaffolds, and ridinilazole, a benzimidazole-based antibiotic, is currently under clinical development [5]. In anticancer research, these scaffolds show activity as tubulin polymerization inhibitors, DNA intercalators, and receptor tyrosine kinase blockers (such as EGFR and VEGFR-2). Furthermore, these scaffolds are present in USFDA-approved drugs like Abemaciclib and Binimetinib, which are currently established as anticancer agents [6,7].

2-Phenylindole was first synthesized by Herman Emil Fischer in 1883, and the reaction is well known by his name: Fischer indole synthesis. The classical Fischer approach first involves the synthesis of phenylhydrazones from acetophenones and then cyclization under acidic conditions [8]. Several acid catalysts, such as HCl, H_2_SO_4_, ZnCl, and polyphosphoric acid, have been reported for the synthesis of 2-phenylindole [9]. Literature reports also suggest that the isolation of phenylhydrazone is not necessary and reaction can proceed from the synthesis of phenylhydrazone to 2-phenylindole in the presence of an acid catalyst as a one-pot reaction [10]. The presence of electron-withdrawing substituents on phenylhydrazine has been reported to hinder the cyclization of hydrazone into indole [11,12]. However, limited literature is available regarding the effect of the substituents present on the aromatic ring of acetophenone.

2-Phenylbenzimidazoles can be synthesized by reacting o-phenylenediamine with either an aromatic aldehyde or any benzoic acid under different conditions [13]. Similar to the synthesis of substituted 2-phenylindoles, the effect of substituents on the aromatic aldehyde or benzoic acid in the synthesis of 2-phenylbenzimidizoles has not been extensively reported in the literature.

In the current study, we investigated synthetic approaches for the N-benzamides of 2-(3-aminophenyl)indole (3-API-B), 2-(4-aminophenyl)indole (4-API-B), 2-(3-aminophenyl)benzimidazoles (3APB-B), and 2-(4-aminophenyl)benzimidazoles (4-APB-B). The synthesized compounds were evaluated for their antibacterial and anticancer potential. Further, we performed an in silico evaluation of the ADMET properties and to assess the probable mode of action on the basis of structural similarity and docking studies followed by MD simulations [5,14].

## 2. Results

### 2.1. Synthesis

**Figure 1:**
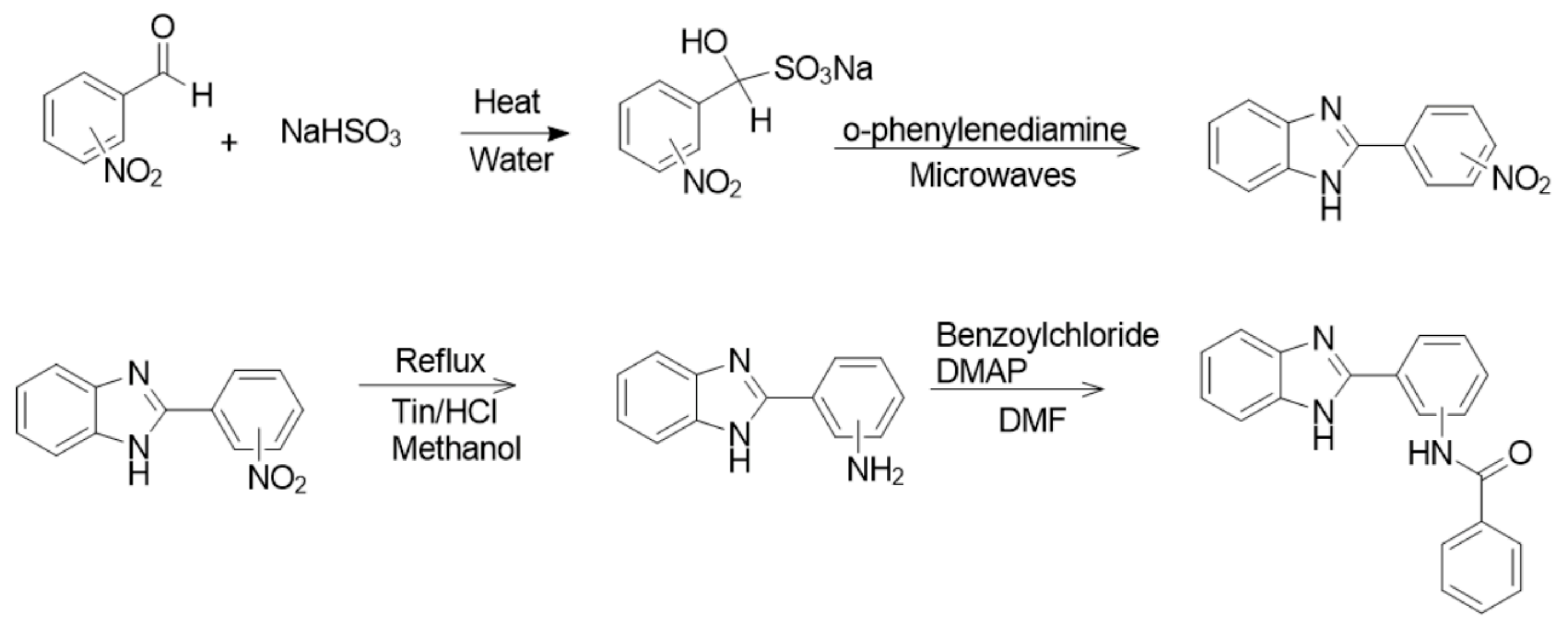
Synthesis of 2-(3&4-aminophenyl)benzimidazole benzamides from 3- and 4-nitrobenzaldehyde.

The synthesis of the benzamide of 2-(3-aminophenyl)benzimidazole (3-APB-B) was carried out by the reduction of 2-(3-nitrophenyl)benzimidazole, which was synthesized by reacting 3-nitrobenzaldehyde with o-phenylenediamine. The resulting amine was reacted with benzoyl chloride to obtain the product 3-APB-B. The benzamide of 2-(4-aminophenyl)benzimidazole 4-APB-B was also synthesized in a similar way [15,16]. The scheme is illustrated in Figure 1.

**Figure 2:**
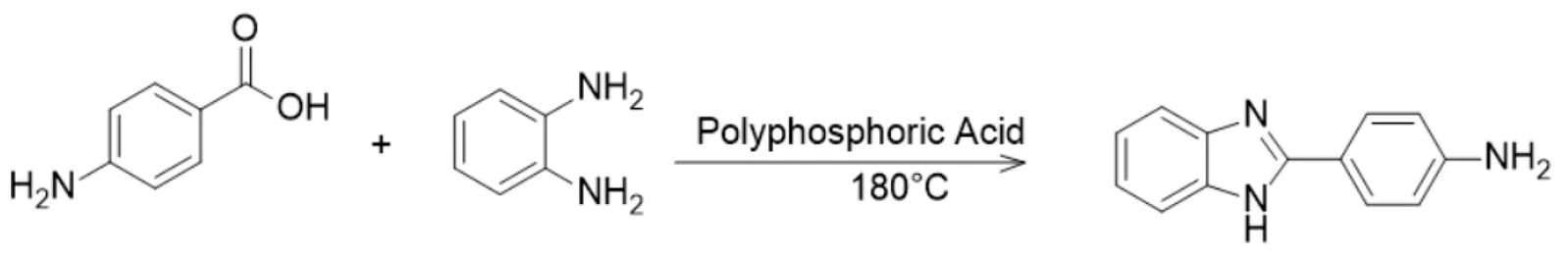
Synthesis of 2-(4-aminophenyl)benzimidazole directly from PABA.

2-(4-aminophenyl)benzimidazole was also directly synthesized from PABA by heating it with o-phenylenediamine in polyphosphoric acid, as shown in Figure 2. It was later reacted with benzoyl chloride to obtain the respective benzamide [16].

**Figure 3:**
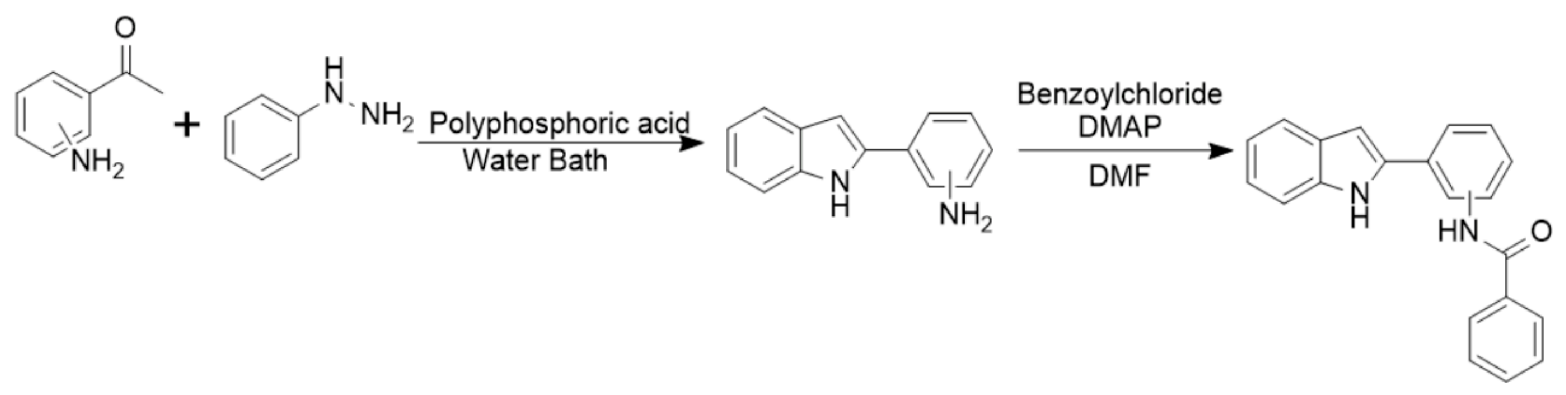
Synthesis of 2-(3&4-aminophenyl)indole benzamides from 3- and 4-aminoacetophenones.

Benzamides of 2-(3&4-aminophenyl)indoles were successfully synthesized by classical Fischer indole synthesis, followed by arylation, as shown in Figure 3.

The structures of the synthesized compounds and their corresponding codes are listed in Table 1.

### 2.2. Characterization

The synthesized compounds were characterized via FTIR, 1H NMR, 13C NMR and ESI Mass Spec in positive mode.

The compounds were dissolved in acetonitrile, and positive ESI mass spectra were obtained using methanol with formic acid as the carrier solvent. The mass spectra indicated the formation of multiple oligomers, and the molecular ion peak was only acquired for the benzimidazole derivatives. In the case of indole derivatives, highly dynamic behavior under positive-ionization conditions was observed, yielding distinct multi-charged oligomeric clusters and cluster adduct matrixes rather than simple standalone monomeric parent ions. FTIR analysis was performed on the solid product by an ATR-FTIR instrument. NMR spectra were obtained using DMSO-d6 as the solvent. NMR spectra were obtained via multiple NMR spectrophotometers with frequencies ranging from 300 MHz to 500 MHz.

### 2.3. Physicochemical Properties

The general physicochemical properties of the synthesized compounds were computationally predicted to assess their drug-likeness and pharmacokinetic profiles. The predicted boiling points (BPs) for all compounds were between 470 °C and 505 °C, predicting them as non-volatile in nature. The hydration free energy ranged from −9.06 to −9.92 kJ/mol, indicating the hydrophobic characters of the compounds. The Log (D) at a pH value of 7.4 spanned from 3.25 to 4.43, suggesting good lipophilicity, while the Log (P) values ranged from 3.97 to 4.53, further confirming the higher degree of lipophilicity of the synthesized compounds. The water solubility, represented by Log (S), was between −5.62 and −6.86, indicating poor water solubility. The melting points (MPs) of the derivatives varied from 240.73 °C to 265.43 °C, consistent with solid-state behavior at room temperature. The pKa values for the acidic characteristic were between 10 and 13, while the pKa values for the basic characteristic were between 4.6 and 5.2, predicting the basic character of the compounds. These predicted parameters provided a comprehensive insight into the molecular behavior of the derivatives and support their potential suitability for further drug development studies, as shown in Table 2.

### 2.4. ADMET Profiling

The absorption potentials of the synthesized compounds were evaluated through in silico pharmacokinetic modeling using the Deep-PK platform. The predicted Caco-2 permeability values (log Paap), which serve as a surrogate for intestinal epithelial transport, fell within a relatively low range (–4.89 to –5.12), indicating moderate passive diffusion. Despite this, the compounds showed strong predicted performances in terms of human intestinal absorption (HIA), with most exhibiting high probability scores (>0.95) with high confidence, suggesting effective GI absorption. Predicted oral bioavailability at the 20% and 50% thresholds indicated that the synthesized compounds have the potential for systemic bioavailability upon oral administration, although confidence levels varied. Benzimidazole derivatives were predicted as potential inhibitors of P-glycoprotein (P-gp), whereas indole derivatives were predicted as non-inhibitors. All four compounds were predicted to have poor skin permeability (log Kp < –2.5), reflecting limited suitability for transdermal drug delivery. These predictive insights underline the favorable oral absorption profiles of the derivatives and provide a basis for prioritizing candidates for subsequent biological evaluation, as shown in Table 3.

To evaluate the pharmacokinetic distribution characteristics, the drug distribution profiles of the synthesized compounds were predicted using the Deep-PK neural network-based ADMET modeling platform. Indole derivatives were predicted to have good blood–brain barrier (BBB) penetration potential, while benzimidazole derivatives were predicted to be non-penetrating in nature. The model predicted that all four compounds would have very low unbound fractions. Plasma protein binding (PPB) was between 85% and 92%, suggesting a good plasma protein binding profile. The predicted steady-state volume of distribution (VDss) values indicated moderate tissue distribution for all four compounds. The results are summarized in Table 4.

The in silico metabolism profiling of the synthesized compounds was assessed similarly by the Deep-PK online server. Indole derivatives were predicted to be BCRP (Breast Cancer Resistance Protein) inhibitors with high confidence. It was predicted that benzimidazole derivatives would not be substrates of most CYP enzymes, indicating that they can have a prolonged half-life. Deep-PK predicted mixed metabolic properties of the compounds with varying levels of confidence. All four compounds were predicted as non-inhibitors of Organic Anion Transporting Polypeptides 1B1 and 1B3 with medium-to-high confidence, indicating that these compounds would not interfere with metabolism or the plasma concentrations of other drugs like statins. The results of the overall metabolic predictions of Deep-PK are summarized in Table 5.

The Deep-PK deep learning algorithm predicted that all compounds would have half-lives of less than 3 h and would be non-inhibitors of organic cation transporter 2. All predictions were made with medium-to-high confidence. The results are summarized in Table 6.

The in silico toxicological profiles of the synthesized compounds were predicted using the Deep-PK online server. The AMES mutagenicity assay prediction indicated that all compounds should be non-mutagenic except 3-APB-B. The compounds were predicted to be the same for both avian species and bees. The Bioconcentration Factor (BCF) for all compounds was predicted as none, suggesting low potential for bioaccumulation. The biodegradation potential was predicted to be safe, indicating that the synthesized compounds would not persist in nature. The carcinogenicity risk remained low in all four compounds. Drug-induced liver injury-I (DILI) predictions revealed that indole derivates have potential hepatotoxicity and that benzimidazole derivates would be safer. Liver injury-II was predicted toxic for all four compounds. Again, indole derivatives were predicted to cause eye irritation while benzimidazole derivates were predicted to be safe for the eyes. All compounds were predicted to be cardio-toxic based on their possible interaction with hERG receptors. All compounds were predicted to be safe for *Daphnia magna.* The compounds are predicted to be nontoxic for fishes. Further, all compounds are predicted to have very mixed types of toxicities for nuclear receptors, as shown in Table 7.

### 2.5. Hierarchical Clustering

**Figure 4:**
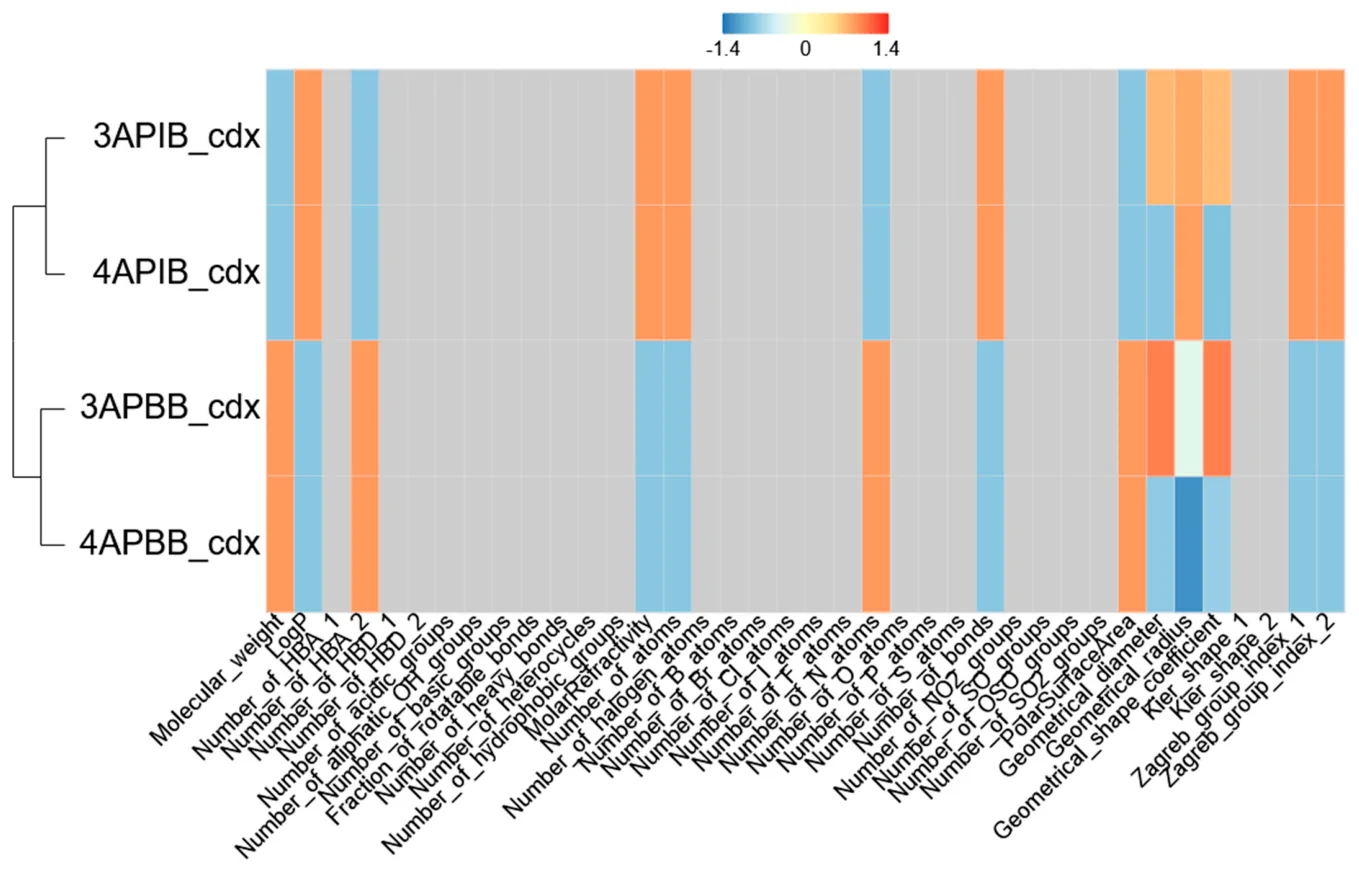
Hierarchical clustering heatmap of synthesized compounds based on selected physicochemical and structural descriptors.

Hierarchical clustering was conducted using a diverse set of molecular descriptors to analyze the structural and physicochemical relationships among the synthesized compounds. Prior to clustering, all descriptor values were normalized using Z-score transformation to eliminate scale-related biases and ensure comparability across different descriptor types. The heatmap (Figure 4) visually illustrates the Z-score-normalized values, highlighting variations among the compounds in terms of molecular weight, hydrogen bond donors and acceptors, rotatable bonds, topological indices, and other geometry-based properties. These clustering trends suggest internal structural similarities within the series and provide a foundation for structure–activity relationship (SAR) interpretation, potentially guiding the further optimization of the scaffold, as shown in Figure 4.

### 2.6. DFT Studies

**Figure 5:**
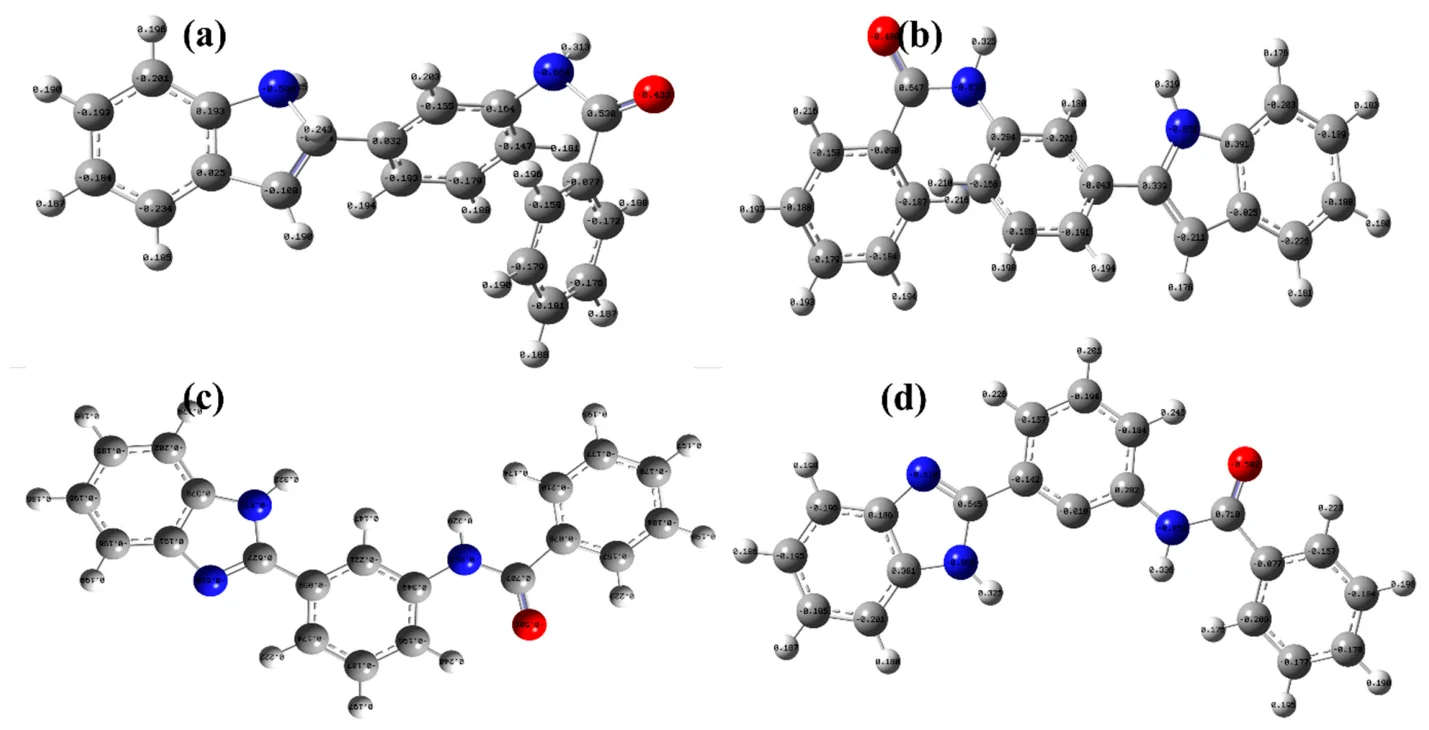
Optimized chemical structures of (**a**) 3-API-B, (**b**) 4-API-B, (**c**) 3-APB-B and (**d**) 4-APB-B simulated using DFT approach B3LYP/6-311(++) level of theory.

To determine the electronic parameters (electronic charge distribution, energies of frontier orbitals, i.e., *E_HOMO_*, *E_LUMO_*) of the ligands, they were simulated and optimized using the DFT/B3LYP level. The lowest-energy-optimized geometry with Mullikan charge distribution is shown in Figure 5, which indicates regions of higher electron density. Mullikan charge analysis revealed the highest charge distribution on oxygen atoms, i.e., [N−0.599], [O −0.433], [O −0.833] and [N, −0.866], demonstrative of a higher electron density concentrated on N & O atoms compared to C and H atoms in any substitution reaction.

#### 2.6.1. Frontier Molecular Orbital (FMO) Analysis

**Figure 6:**
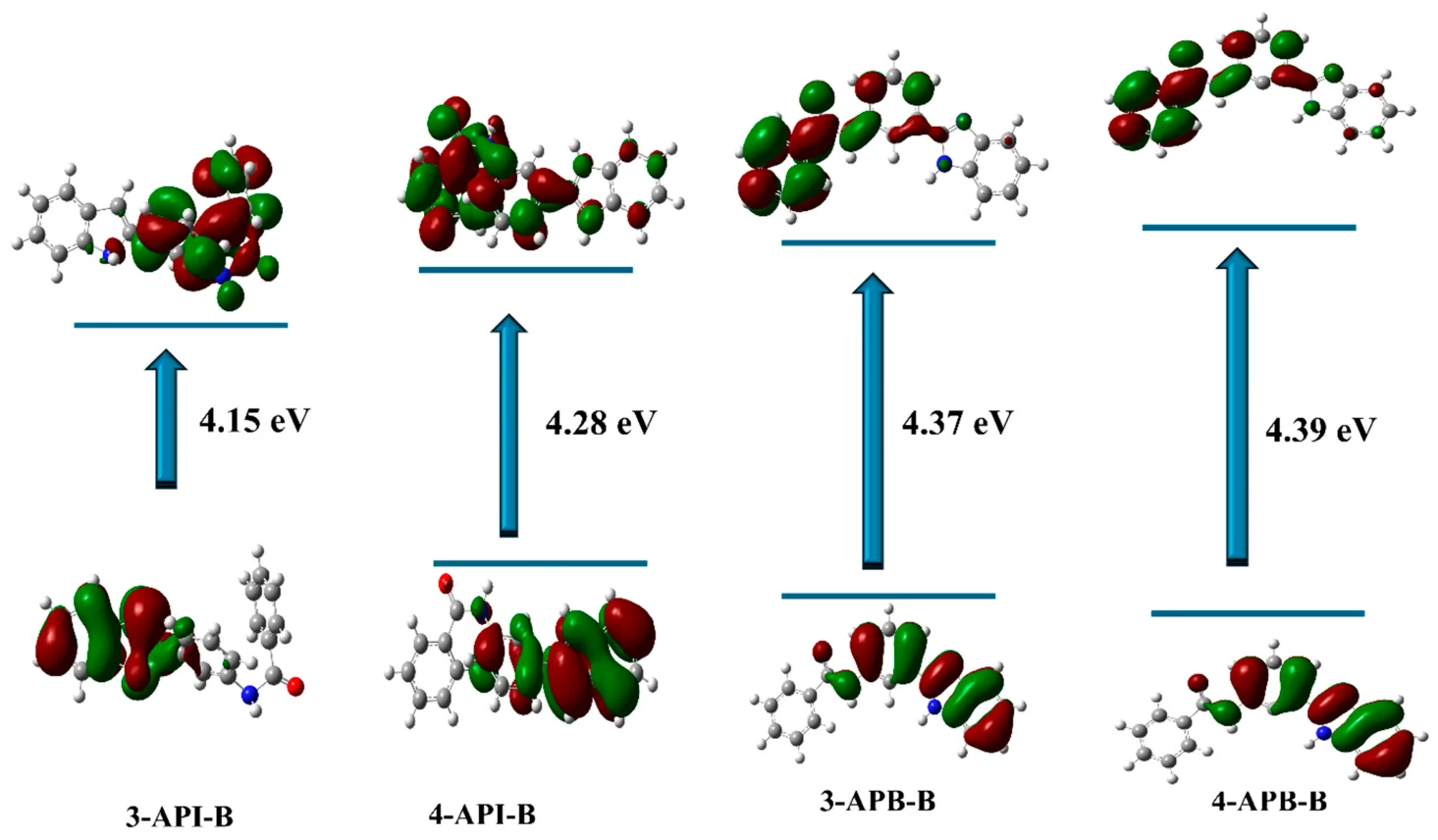
HOMO and LUMO orbital density distributions and their associated energy gaps.

The quantum chemically optimized 3-API-B, 4-API-B, 3-APB-B, and 4-APB-B compounds were subjected to electronic structure analysis to determine the distribution of their frontier molecular orbitals (FMOs). Frontier molecular orbital calculations provide a comprehensive understanding of orbital characteristics, including spatial distribution, nodal features, and the contribution of individual atoms to the electronic structure. This approach is widely employed for predicting molecular electronic transitions, charge transfer behavior and the reactivity of a molecule. As represented in Figure 6, for the 3-API-B, 4-API-B, 3-APB-B, and 4-APB-B compounds, the calculated energies of the HOMO were −4.88 eV (paired), −5.50 eV (paired), −5.77 eV (paired), and −5.85 eV (paired) eV, respectively, whereas the calculated energies of the LUMO were −0.725 eV, −1.220 eV, −1.398 eV and −1.461 eV, respectively.

*E_HOMO_* and *E_LUMO_* values give an estimate of the electron-donating or electron-accepting character of a given compound. Consequently, a compound is considered more electron-donating as the value of its *E_HOMO_* increases and is considered more electron-accepting as the value of its *E_LUMO_* decreases. Hence, the electron-donating abilities of the compounds vary as 3-API-B > 4-API-B > 3-APB-B > 4-APB-B, while the electron-accepting characteristics vary as 3-API-B < 4-API-B < 3-APB-B < 4-APB-B. Furthermore, the electron density distributions of the HOMO are predominantly localized over the highly electronegative oxygen and nitrogen atoms, highlighting their significant role in the electronic transitions and charge transfer characteristics of the complex, while the electron density distributions of the LUMO are concentrated on atoms other than oxygen and nitrogen.

The corresponding HOMO–LUMO energy gaps (**ΔE**) are presented in Table 8, which were found to increase in the order 3-API-B < 4-API-B < 3-APB-B < 4-APB-B, indicating that π-electron excitation can readily occur for 3-API-B, thereby facilitating efficient electron transfer processes and rendering the highest activity of 3-API-B in electron transfer reactions.

#### 2.6.2. Molecular Electrostatic Potential Analysis

**Figure 7:**
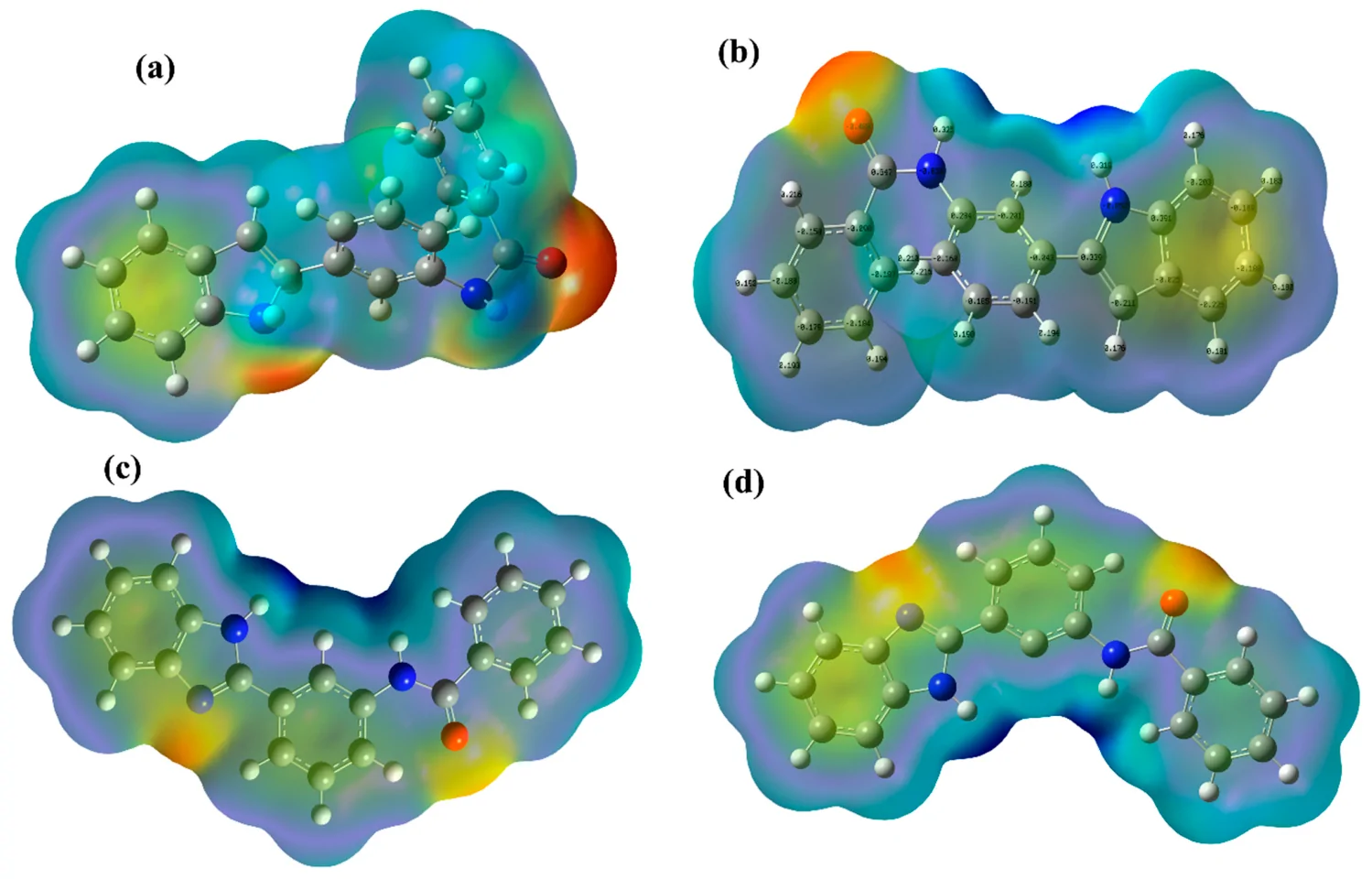
Molecular electrostatic potentials of (**a**) 3-API-B, (**b**) 4-API-B, (**c**) 3-APB-B and (**d**) 4-APB-B simulated using DFT approach B3LYP/6-311(++) level of theory.

Another significantly important electronic parameter is the molecular electrostatic potential surface (MESP), which provides information on the electrophilic and nucleophilic characteristics of the molecule. Molecular electrostatic potential surfaces (MESPs) optimized at the B3LYP/6-311(++) level of theory were implemented to map the nucleophilic and electrophilic moieties of 3-API-B, 4-API-B, 3-APB-B and 4-APB-B, as shown in Figure 7.

It is evident from Figure 7 that negative potential is concentrated on nitrogen and oxygen atoms (reddish yellow), hence imparting a nucleophilic character to O and N atoms, whereas positive potential is located on C and H atoms (green and blue, respectively), indicating their electrophilic character in chemical reactions with electron-rich enzymes in the anticancer and antibacterial assay. These positive- and negative-potential regions provided information about the sites involved in intermolecular interactions, implying that O and N atoms have an electron-donating character in all four compounds. From Figure 7, it is evident that the nitrogens of the benzimidazole and indole rings do not have that much electron density compared to the nitrogen atoms of amide linkage. This finding is in line with experimental findings indicating that these nitrogens do not easily participate in nucleophilic reactions.

### 2.7. Docking

**Figure 8:**
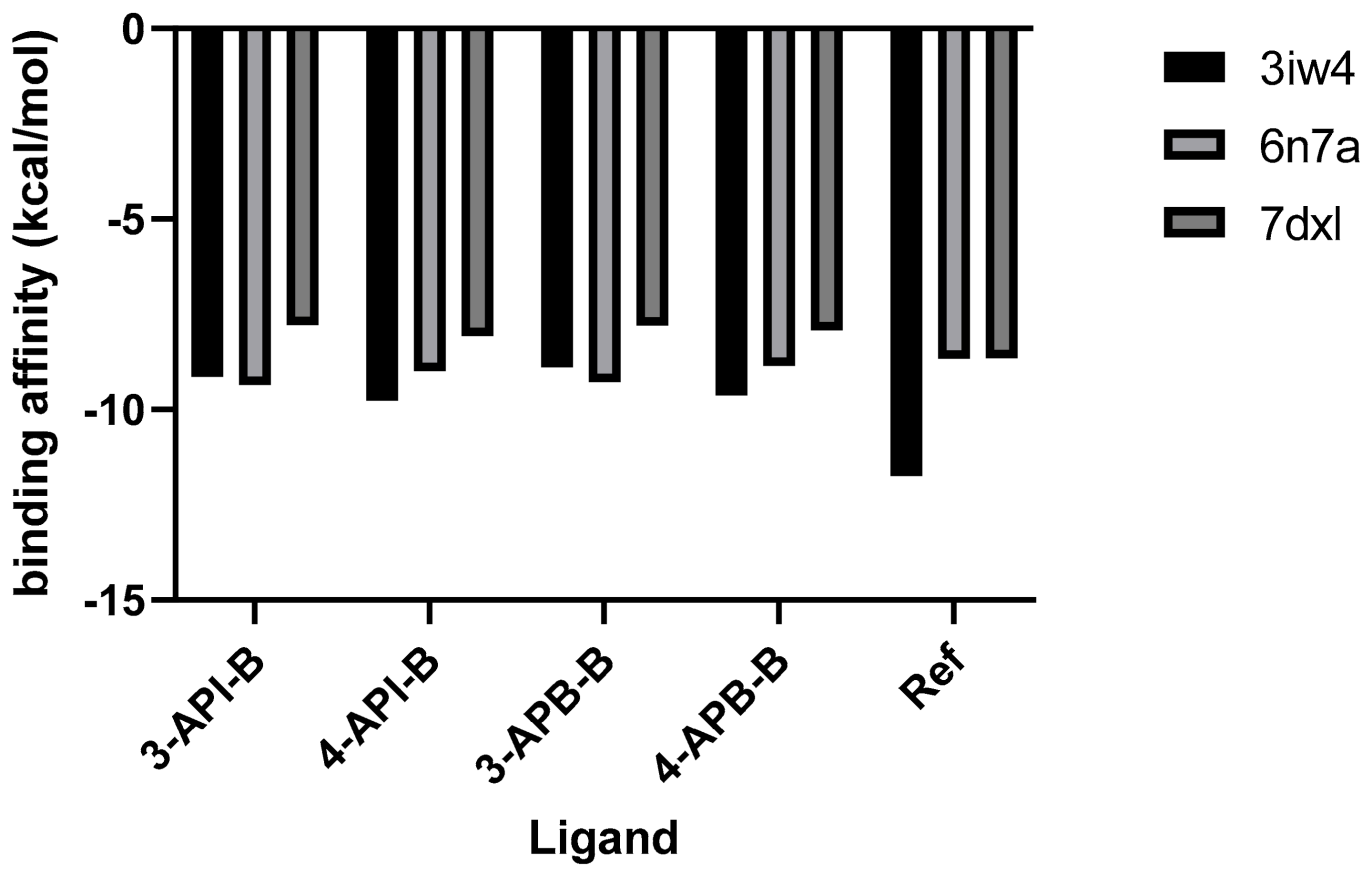
Maximum binding affinities of synthesized compounds and reference ligands against kinases present in HeLa, PC3 and 3T3 cells.

In the literature, the most abundant kinase found in HeLa cells that has been proven to be targeted by already-approved kinase inhibitors is JAK-1 (6N7A) [17]. Furthermore, AXL inhibitors have been reported to have significant effects on prostate cancer cells, and due to this, the AXL kinase (7DXL) was selected as a target for PC3 cell lines [18]. In 3T3 cells, PKC-alpha is abundantly expressed; for this reason, PKC-alpha (3IW4) was also selected for docking [19]. The co-crystallized ligand with each protein structure was used as a reference to compare the docking score and pose. The docking results are summarized in Figure 8.

The docking results indicated that all the compounds should have almost equally good cytotoxic effects against each cell line, but the in vitro results are different from this. This may be due to physical factors, such as the solubility of the molecule, or any physiologic effect, such as the inability of the cells to absorb the specific compound, because while the analysis of the compounds was performed, it was observed that these compounds have very poor solubility in both organic and aqueous solvents. The docking poses of all the ligands were almost identical for all three proteins and were comparable to those of the co-crystallized ligands. The docking results are also summarized in Table 9.

### 2.8. MD Simulations

The structural stability, conformational flexibility, and binding interactions of three distinct protein–ligand complexes (the 3IW4-4API-B complex, referred to as Complex A, the 6N7A-3API-B complex, referred as Complex B, and the 7DXL-4API-B complex, referred as Complex C) were evaluated using a 100 ns molecular dynamic simulation trajectory.

#### 2.8.1. Structural Stability (RMSD and Radius of Gyration)

**Figure 9:**
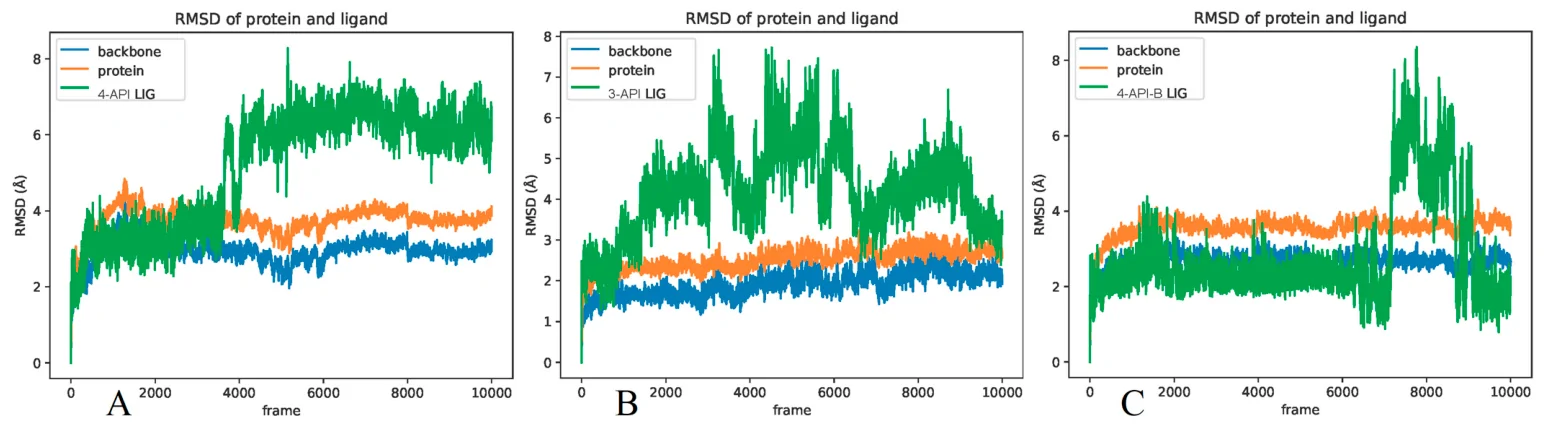
Root-Mean-Square Deviation (RMSD, in Å) profiles calculated for protein backbone (blue), total protein (orange), and specific bound ligand structures (green) across 10,000 frames for (**A**) 3IW4-4API-B complex, (**B**) 6N7A-3API-B complex, and (**C**) 7DXL-4API-B complex.

The structural equilibrium was evaluated via backbone, protein, and ligand RMSD profiles (Figure 9).

Complexes **A** and **C** displayed stable protein backbones, maintaining an average value around 2.5–3.0 Å; however, their respective ligand (4-API-B) experienced prominent structural shifts (jumping up to ~6–8 Å) after frame 4000 (Complex A) and frame 7000 (Complex C). This signifies a significant conformational rearrangement or pocket reorientation within the binding site. In contrast, Complex B showed continuous, dynamic ligand fluctuations throughout the entire simulation, reaching spikes up to 7.5 Å, indicating a more flexible binding mode.

**Figure 10:**
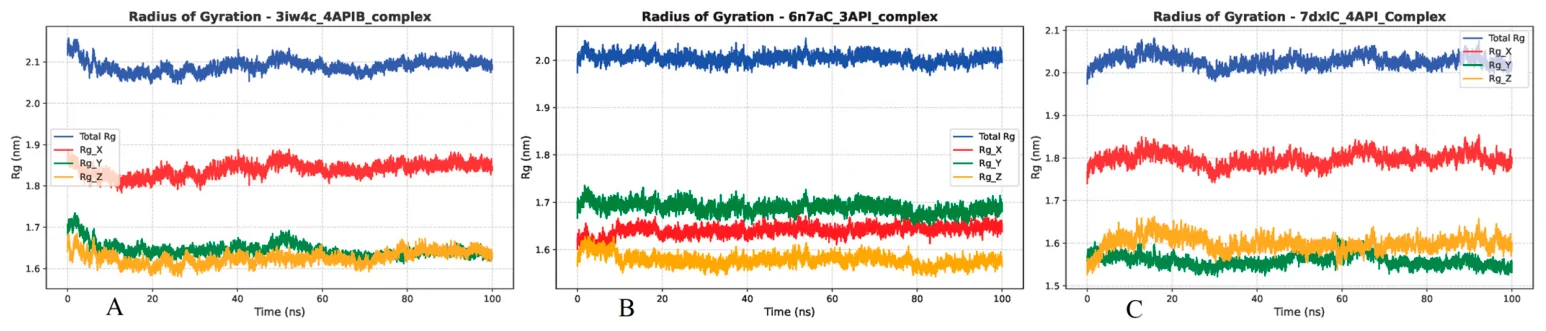
Radius-of-gyration profiles of three complexes over 100 ns molecular dynamic trajectory. (**A**) 3IW4-4API-B complex, (**B**) 6N7A-3API-B complex, and (**C**) 7DXL-4API-B complex.

The compactness of the structures remained constant across all systems and was evaluated by the radius of gyration (Figure 10). Complex A stabilized at 2.1 nm, Complex B at 2.0 nm, and Complex C at 2.02 nm. The lack of sharp drops or continuous upward trends confirms that none of the proteins underwent major structural changes or unfolding during the whole 100 ns simulation.

#### 2.8.2. Local Residue Flexibility (RMSF)

**Figure 11:**
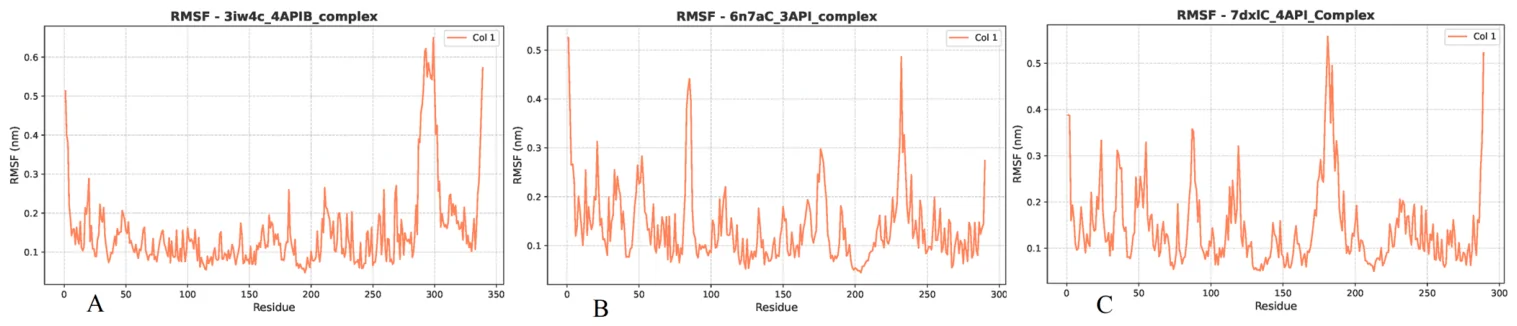
Root-Mean-Square Fluctuation (RMSF, nm) values plotted against amino acid residue positions to show local residue flexibilities within (**A**) 3IW4-4API-B complex, (**B**) 6N7A-3API-B complex, and (**C**) 7DXL-4API-B complex.

The Root-Mean-Square Fluctuation (RMSF) profiles (Figure 11) represent the localized flexibility of individual amino acid residues. Complex A exhibited highly rigid core regions (RMSF <0.2 nm) but displayed a flexible domain region between residues 280 and 310, peaking above 0.6 nm. Complex B showed a more distributed flexibility profile, with three distinct loop regions fluctuating around 0.3–0.5 nm (near residues 60 and 230). Complex C showed a localized flexible loop region near residue 180, peaking close to 0.6 nm, while the rest of the protein remained rigid.

#### 2.8.3. Solvent-Accessible Surface Area (SASA)

**Figure 12:**
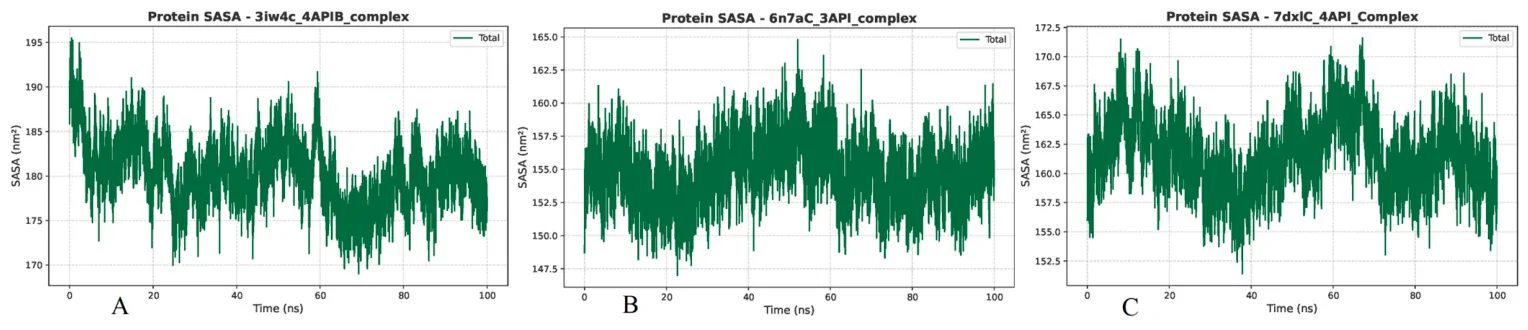
Total Solvent-Accessible Surface Area (SASA) profiles monitoring global surface accessibility variations of target proteins for (**A**) 3IW4-4API-B complex, (**B**) 6N7A -3API-B complex, and (**C**) 7DXL-4API-B complex.

The surface exposure and structural expansions were evaluated via SASA metrics for both ligands and proteins. The solvent-accessible surfaces for all three ligands remained steady between 5.25 nm^2^ and 6.50 nm^2^. This suggests that despite the significant ligand movements seen in the RMSD plots, the ligands remained buried within the protein’s binding pockets (Figure 12).

**Figure 13:**
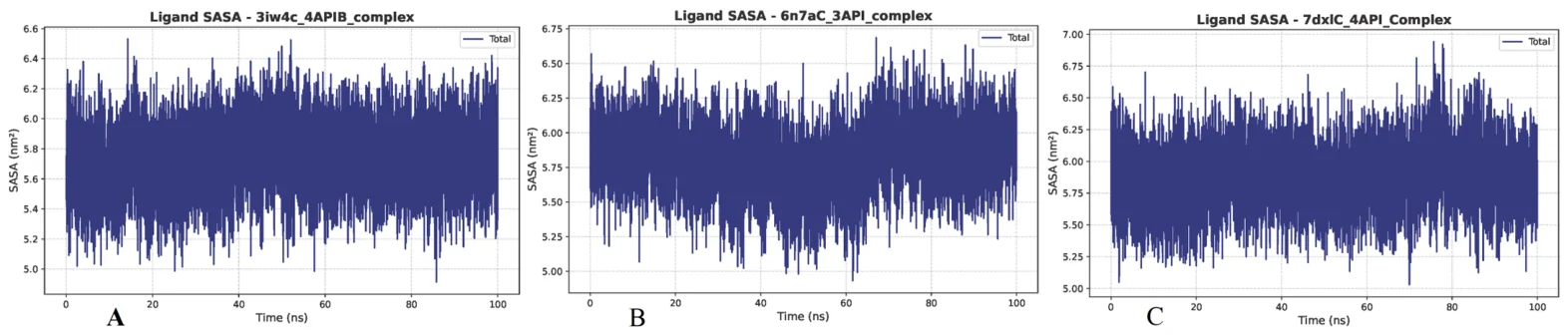
Solvent-Accessible Surface Area (SASA) fluctuations tracking structural exposure profiles of bound ligand molecules within (**A**) 3IW4-4API-B complex, (**B**) 6N7A-3API-B complex, and (**C**) 7DXL-4API-B complex over 100 ns.

The overall protein surface exposure remained constant for the whole simulation (Figure 13). Complex A showed a steady decrease in the SASA from 190 nm^2^ to ~175 nm^2^, reflecting a structural shrinkage or pocket closure. Complexes B and C remained stable around 155 nm^2^ and 162.5 nm^2^, respectively.

#### 2.8.4. Hydrogen-Bonding Network and Secondary Structure (DSSP)

**Figure 14:**
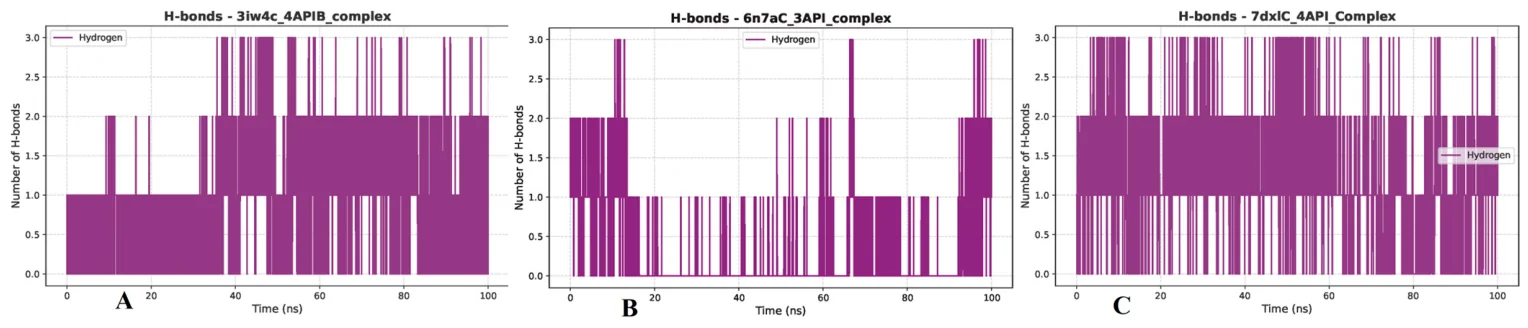
Number of active hydrogen bonds formed between respective host protein and localized ligand frames across (**A**) 3IW4-4API-B complex, (**B**) 6N7A-3API-B complex, and (**C**) 7DXL-4API-B complex.

The complex stability is highly dependent on hydrogen bonding between ligands and proteins (Figure 14). Complex A maintained active continuous 1 to 3 H-bonds, with the enhanced occurrence of H-bonding in the latter half of the simulation. Complex B suffered from a loss of direct H-bonding between 15 ns and 90 ns (dropping to 0–1 bonds), which explains the high ligand RMSD fluctuations noted earlier. Complex C showed a highly dense, continuous occurrence of 1 to 3 H-bonds throughout the entire 100 ns window, suggesting strong 3-D binding.

**Figure 15:**
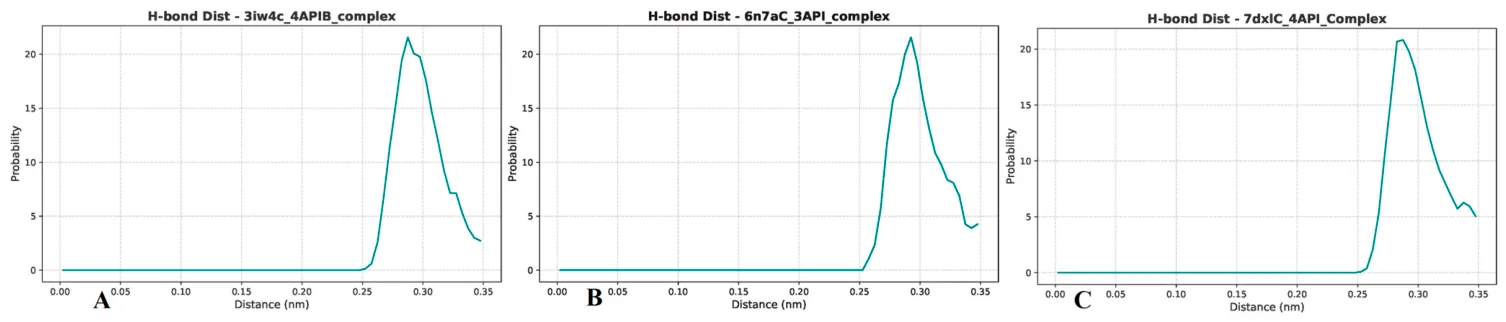
Structural probability distribution mapping of intermolecular hydrogen bond distances (nm) emphasizing localized bonding constraints for (**A**) 3iw4c-4API-B complex, (**B**) 6N7A-3API-B complex, and (**C**) 7DXL-4API-B complex.

The hydrogen bond distance profiles were found to be identical across all three complexes, exhibiting a donor-acceptor distribution peak at 0.29 nm (within the classic strong electrostatic distance threshold of <0.35 nm) (Figure 15).

**Figure 16:**
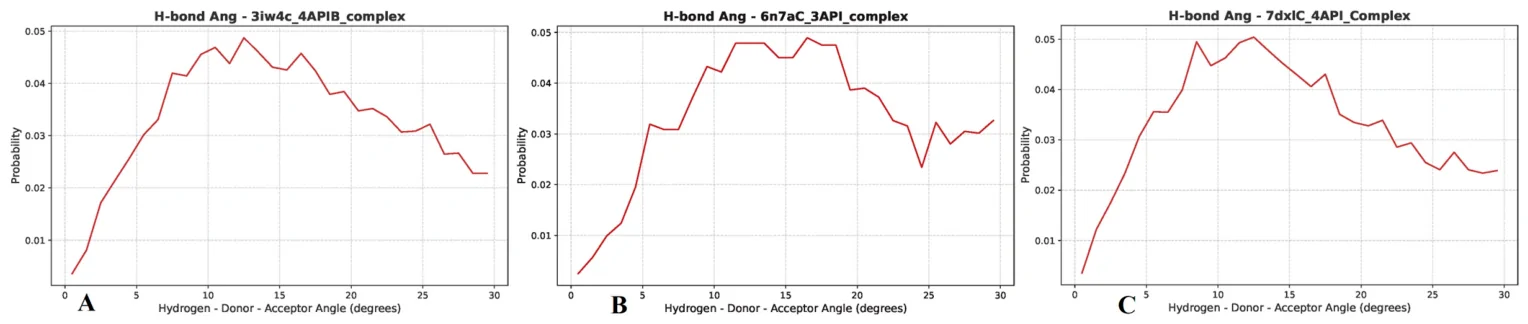
Probability distribution functions of hydrogen bond donor–acceptor angles (degrees) recorded over simulation history for (**A**) 3IW4C-4API-B complex, (**B**) 6N7AC-3API-B complex, and (**C**) 7DXLC-4API-B complex.

The donor-acceptor angles show wide peaking between 8° and 15°, confirming geometrically ideal hydrogen bonds across all systems (Figure 16).

**Figure 17:**
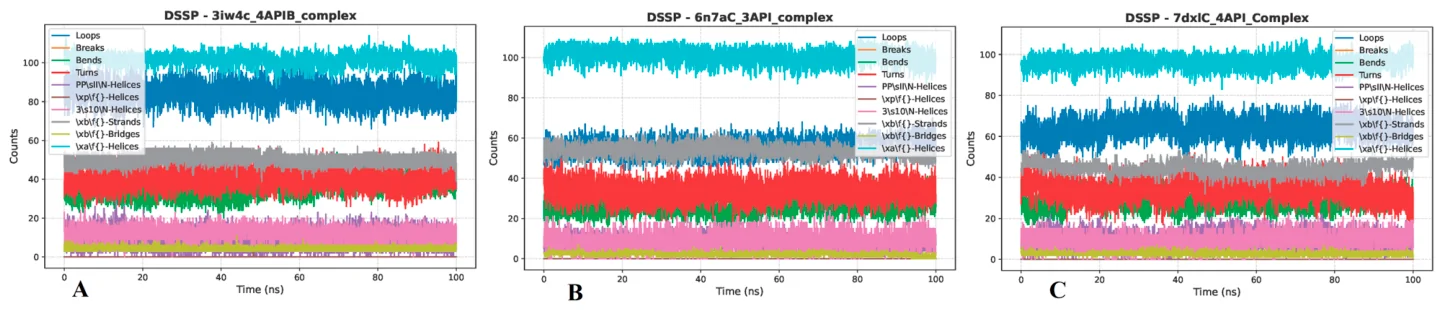
Secondary-structure composition tracking as function of simulation time (100 ns) using DSSP algorithm for (**A**) 3IW4-4API-B complex, (**B**) 6N7A-3API-B complex, and (**C**) 7DXL-4API-B complex. Color-coded regions quantify total counts of loops, breaks, bends, turns, and varying helical/strand structural elements.

The secondary-structure counts (loops, turns, alpha-helices, beta-strands) remained constant across the full 100 ns time period for all three systems (Figure 17). The structural integrity of the protein matrix remained preserved, indicating that ligand conformational shifts do not disrupt or deform the protein’s core secondary elements.

### 2.9. In Vitro Assays

#### 2.9.1. In Vitro Cytotoxic Activity

The in vitro cytotoxic and anticancer activities of the benzamide derivatives were assessed in the HeLa, PC3 and 3T3 cell lines via the MTT assay [20]. In all the assays, doxorubicin was used as a positive control. The IC_50_ values are given in Table 10.

In the case of in vitro activity against the HeLa cell line, the concentrations of the positive control (doxorubicin) and test compounds used were 1 µM, 10 µM and 100 µM. At the maximum concentration, 3-API-B inhibited 71.59%, 4-API-B inhibited 31.41%, 3-APB-B inhibited 84.44%, 4-APB-B inhibited 93.94%, and the positive control inhibited 93.66%.

**Figure 18:**
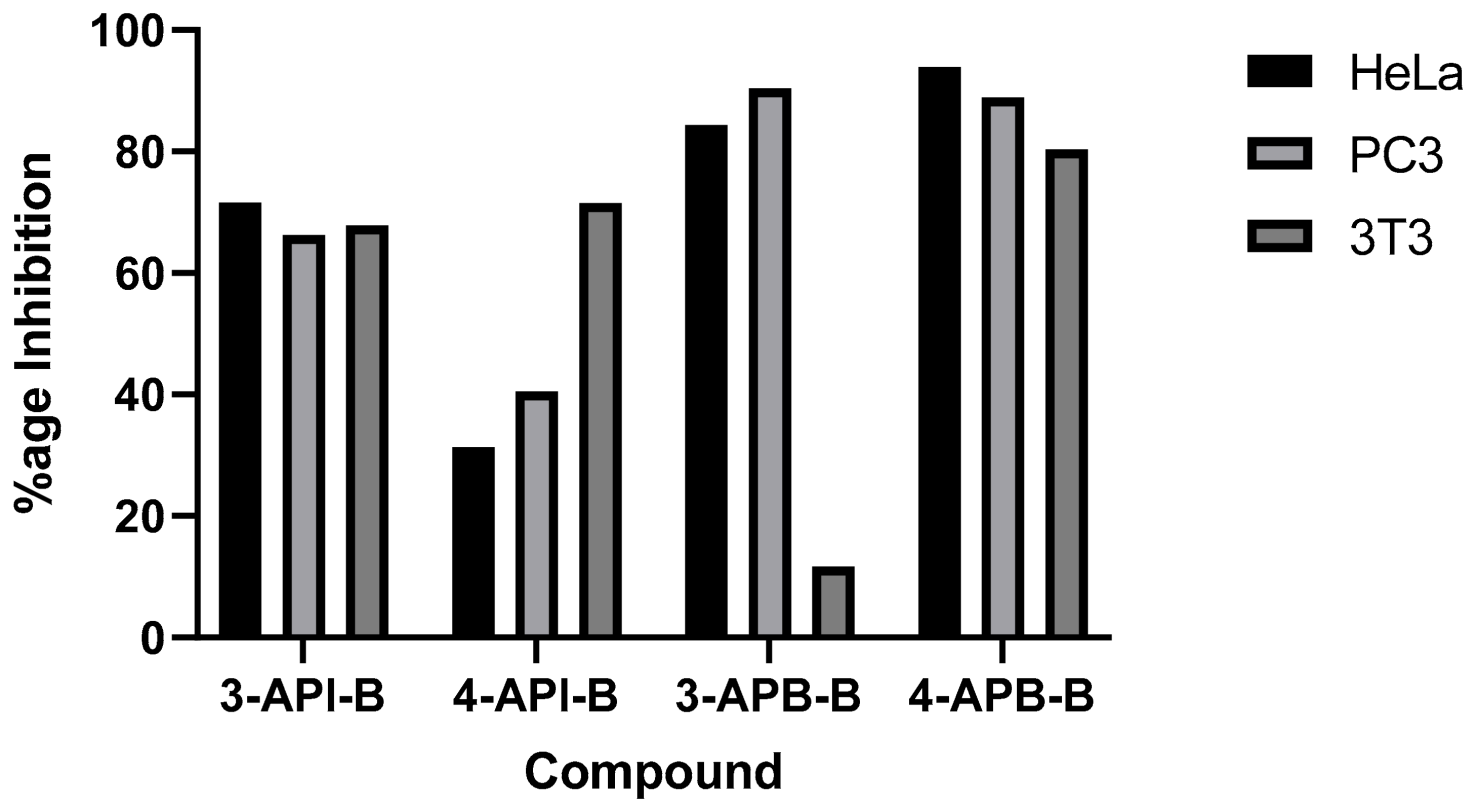
Percent inhibitions of HeLa, PC3 and 3T3 cell lines at 100 μM.

In terms of activity against the PC3 and 3T3 cell lines, the concentrations of the test compounds used were the same as those used for activity against the HeLa cell lines, but the maximum concentrations of doxorubicin used were 10 µM and 30 µM, respectively. In the MTT assay against PC3 cell lines at maximum concentrations, 3-API-B inhibited 66.28%, 4-API-B inhibited 40.52%, 3-APB-B inhibited 90.47%, 4-APB-B inhibited 88.94%, and doxorubicin inhibited 58.01%. The maximum inhibition rates observed against the 3T3 cell line were 67.86% for 3-API-B, 71.51% for 4-API-B, 11.71% for 3-APB-B, 80.39% for 4-APB-B and 95.8% for doxorubicin. A comparison of the IC50 values of the synthesized compounds with those of doxorubicin revealed that 4-APB-B has considerable activity against the HeLa cell line and is not highly toxic to 3T3 cells. A graphical comparison of the percentage inhibitions shown by all compounds at a concentration of 100 µM against the abovementioned cell lines is given in Figure 18.

In cases where the maximum inhibition observed was less than 50%, the IC_50_ values could not be calculated, and that compound was considered inactive against that specific cell line.

3-API-B had mild inhibitory effects on all three cell lines. 4-API-B exhibited selective toxicity exclusively toward the noncancerous 3T3 fibroblast cell line (IC_50_ = 71.91µM) while showing very little therapeutic efficacy (at only higher concentrations) against both target cancer lines. Conversely, 3-APB-B displayed a highly favorable selective profile, demonstrating moderate anticancer potency against the HeLa (23.97 µM) and PC3 (33.81 µM) lines while remaining almost nontoxic towards healthy 3T3 cells. 4-APB-B showed moderate-to-good activity against all the cell lines. The IC_50_ values indicate that 4-APB-B is active against HeLa cell lines (5.48 µM) and that 3-APB-B has moderate activity but only against cancer cell lines.

#### 2.9.2. Antibacterial Activity

All the synthesized compounds were screened against *Escherichia coli* (ATCC 25922), *Bacillus subtilis* (ATCC 23857), *Staphylococcus aureus* (NCTC 13277), *Pseudomonas aeruginosa* (ATCC 10145), and *Salmonella typhi* (ATCC 14028) at a concentration of 200 mcg/mL and were found to be completely inactive against these strains. These results indicated that further antibacterial screening or activities might not be beneficial.

## 3. Discussion

### 3.1. Synthesis

On the basis of the available literature, it was hypothesized that if benzoyl chloride reacts with 2-(4-aminophenyl)indole and 2-(3-aminophenyl)indole for the synthesis of the respective benzamides, then there would be the probability of acylation at position 1 and 3 also [21]. In view of this, it was initially planned to first synthesize 2-(4-nitrophenyl)indole, methylate it with DMS, reduce it to methylated 2-(4-aminophenyl)indole, and then react it with benzoyl chloride.

To execute the above scheme, 2-(4-Nitrophenyl)indole synthesis was attempted via Fischer indole synthesis. 4-nitroacetophenone and phenylhydrazine were heated in ethanol till boiling, and then a few drops of HCl were added. The addition of HCl immediately resulted in the formation of bright-red crystals of 4-nitroacetophenone phenylhydrazone, which were filtered and washed with distilled water and then with 1% HCl solution to remove any unreacted phenylhydrazine. The conversion of the 4-nitroacetophenone phenylhydrazone to the respective indole was attempted by using multiple catalysts reported in the literature, but none of them worked, as they usually work in the case of simple acetophenone phenylhydrazone. Refluxing with HCl or sulfuric acid did not result in any change. Refluxing with zinc chloride in ethanol resulted in the formation of zinc oxide, which was difficult to separate from the rest of the reaction mixture because of the solubility issues associated with zinc oxide. The addition of polyphosphoric acid resulted in the ignition of the reaction mixture and carbonization. The same phenomenon was observed with 3-nitroacetophenone phenylhydrazone, which was prepared in a similar way to that of 4-nitroacetophenone phenylhydrazone. 3-Nitrophenylacetophenone hydrazone was bright orange in color and crystalline in nature. Another observation was that 3-nitroacetophenone phenylhydrazone and 4-nitroacetophenone phenylhydrazone were more chemically stable than other phenylhydrazones. Phenylhydrazones of other acetophenones, such as 3-aminoacetophenone, 4-aminoacetophenone and unsubstituted acetophenone, would turn into black-tar-like materials if they were placed in open air or in water overnight, but 3-nitroacetophenone and 4-nitroacetophenone phenylhydrazones remained intact in open air during the course of this study, i.e., 3 years. After multiple attempts, the reaction was attempted with iron metal dust, as, in some studies, indole synthesis is reported in the presence of FeCl_3_ [22]. We refluxed the 3-nitro- and 4-nitroacetophenone phenylhydrazones with iron dust and 37% HCl in ethanol for 8 h. After 8 h, the ethanol was removed with a rotary evaporator. HCl was neutralized with a sodium carbonate solution to a pH of 7 to 8, which resulted in black-to-brown precipitates. The precipitates obtained from the reaction of 4-nitroacetphenone phenylhydrazone were sent for NMR analysis. The proton NMR spectrum obtained had very broad peaks due to shim issues caused by iron present in the samples, so it could not be used for any structural analysis. The products were subsequently sent for atomic absorption analysis to determine the iron content. The results revealed that the 4-nitroacetophenone phenylhydrazone product and 3-nitroacetophenone phenylhydrazone product contained elemental iron at concentrations of 54% and 52%, respectively. This indicated the formation of iron–metal complexes of 3-nitro- and 4-nitroacetophenone phenylhydrazones.

When we were unsuccessful at synthesizing 2-(3-nitrophenyl)indole and 2-(4-nitrophenyl)indole, we switched over to the synthesis of 2-(3-nitrophenyl)benzimidazole and 2-(4-nitrophenyl)benzimidazoles. In the literature, there are multiple methods for the synthesis of substituted 2-phenylbenzimidazoles from o-phenylenediamine and substituted benzaldehydes. In some methods, DMSO is used as the solvent, and in some methods, hydrogen peroxide is used [13,23]. We successfully synthesized 2-(3-nitrophenyl)benzimidazole and 2-(4-nitrophenyl)benzimidazole in good yields by first generating a bisulfite adduct of aldehyde in distilled water and then cooling it to room temperature, adding o-phenylenediamine to the mixture and irradiating it with microwaves for 10–20 min in a household microwave oven [24]. 2-(4-Nitrophenyl)benzimidazole was also synthesized by boiling a 4-nitrobenzaldehyde bisulfate adduct solution in water and adding o-phenylenediamine while the mixture was boiling, and the product formed immediately. This approach did not work in the case of 3-nitrobenzaldehyde, which was synthesized only by microwave irradiation. In the case of aldehydes, the synthesis of the bisulfite adduct is necessary for product formation; if the bisulfite adduct is not made and the aldehyde reacts directly with o-phenylenediamine by either heating to high temperatures or by microwave irradiation, other purplish-to-pink-colored products are obtained. Water as a solvent was found to be the best for these reactions. We used sodium metabisulfite to make a bisulfite solution. Higher concentrations of sodium metabisulfite resulted in better yields. 2-(4-nitrophenyl)benzimidazole (4-NPB) was highly fluorescent under 366 nm UV light, while 2-(3-nitrophenyl)benzimidazole (3-NPB) did not show any fluorescence.

On the basis of the same assumption that we would have to first methylate nitrogen at position 1 and then reduce the nitro group to the amino group for the synthesis of benzamides, 3-NPB and 4-NPB were methylated via DMS and a biphasic solvent system to add a methyl group at position 1 [25]. In both cases, dull-yellow products with very poor solubility in most solvents, including DMF and acetonitrile, were obtained. Both products were soluble in water at extremely basic and acidic pH values. Furthermore, the fluorescence of 4-NPB decreased under 366 nm UV light after the methylation reaction. Owing to the poor solubility of the resulting products, it was not feasible to use them further in our reactions, so we tried to proceed without methylating them.

3-NPB and 4-NPB were reduced to their respective amines [2-(3-aminophenyl)benzimidazole (3-APB) and 2-(4-aminophenyl)benzimidazole (4-APB)] by tin/HCl [25]. After reduction, the fluorescence of 4-APB under 366 nm UV light was much lower than that of 4-NPB; 3-APB was highly fluorescent when precipitated as HCl salt and weakly fluorescent if it was treated with a base. 2-(4-Aminophenyl)benzimidazole (4-APB) was also synthesized from para-aminobenzoic acid (PABA); in this case, a few methods reported in the literature, such as the use of hydrogen peroxide or reactions at high temperatures in DMSO or DMF, have been reported [26,27], but all these methods resulted in the formation of red products that did not have the same properties as the 4-APB obtained from the reduction of 4-NPB. Direct synthesis from PABA was successfully carried out by mixing it with o-phenylenediamine in polyphosphoric acid and heating it to 180 °C on a hotplate for 30 min. The thick mixture was then poured into ice-cold distilled water, the pH was neutralized with any appropriate base, and the precipitates were collected. The 2-phenylbenzimidazole derivatives, especially amino derivatives, were found to be water-soluble in both the highly acidic and basic pH ranges. This sometimes makes them difficult to isolate from reaction mixtures, especially after reduction with tin/HCl. In the case of the reaction of PABA with o-phenylenediamine in polyphosphoric acid, if the product did not precipitate after the addition of distilled water (in the case where excess water was used), the pH was first neutralized by the slow addition of a sodium carbonate solution (which causes violent effervescence if added too quickly) or an ammonia solution, NaCl was added to saturate the solution, the mixture was kept overnight, and the next day, the precipitates were filtered and dried.

3-APB and 4-APB, which were obtained by reducing their respective nitro compounds, were also difficult to isolate because of their water solubility. The standard method of reducing aromatic nitro compounds to their respective amines is the strong basification of the reaction mixture with sodium hydroxide so that tin-II chloride is converted to sodium stannate, i.e., made water-soluble [28], but in our case, our product was also water-soluble at those pH values where the sodium stannate was formed. At lower pH values, such as approximately 7, hydrated tin oxide also precipitated out with the product. Both are soluble in DMF and insoluble in other organic solvents and water, making it very difficult to separate them. The best approach identified was to simply evaporate ethanol/methanol from the reducing reaction mixture and then cool it down, resulting in the formation of precipitates of 3-APB and 4-APB hydrochloride salts. These samples were filtered and dried. The hydrochloride salts were water-soluble and could not be washed with distilled water. They had a brighter-yellow color than their respective base forms. They can be dissolved in water and then basified to obtain precipitates of the respective bases.

Both 3-APB and 4-APB were reacted with excess benzoyl chloride in DMF as the solvent. DMAP was used as the catalyst and TEA was used as the base to assess whether acylation is possible with 3-APB and 4-APB simultaneously on nitrogen at position 1 and at the amino group present at the third and fourth positions of the 2-phenyl ring. The reaction was initially started in an ice bath, and the temperature was gradually allowed to rise up to room temperature. The reaction mixture was stirred continuously. After 24 h, the reaction mixture was poured into distilled water and stirred for another 24 h to degrade excess benzoyl chloride to benzoic acid. After 24 h, the pH of the reaction mixture was increased to 8 by the addition of a sodium carbonate solution or an ammonia solution to make a water-soluble salt of benzoic acid, after which the precipitates were filtered and air-dried. Upon the proton and carbon NMR analysis of both products, it was observed that acylation had occurred only once, and it was most likely at the amino group present at the third and fourth positions of the 2-phenyl ring. The monoacylation was further verified by ESI mass spectrometry.

The results of the acylation of 3-APB and 4-APB led us to hypothesize that we might observe similar behavior in the case of 2-phenylindoles, in which positions 1 and 3 could be resonance-stabilized and are less reactive when a phenyl ring is present at position 2, suggesting that we could synthesize 2-(3-aminophenyl)indole (3-API) and 2-(4-aminophenyl)indole (4-API) from meta- and para-amino acetophenones in polyphosphoric acid and then react them with excess benzoyl chloride in the same way that we did with 3-APB and 4-APB. We proceeded with this, and the NMR spectra revealed the same results: only monoacylation occurred. These observations indicate that the presence of a 2-phenyl group reduces the reactivity at positions 1 and 2 in the benzimidazoles and indoles, respectively.

Furthermore, we observed that temperature control is essential for Fischer indole synthesis, and the reaction does not take much time to complete. A specific temperature was required to start the process of the indolization of phenylhydrazone; if the reaction temperature was not met, the reaction would not start. Once the reaction has started, it is exothermic and tends to overheat and carbonize. In the case of 3- and 4-nitroacetophenone phenylhydrazones, the starting temperature was much higher and had a much greater tendency for carbonization or autoignition because of the presence of nitro groups than in the case of 3 and 4 aminoacetophenones. Owing to this autoignition and carbonization, we were unable to synthesize indoles from 3- and 4-nitroacetophenone phenylhydrazones, while 3- and 4-aminoacetophenone phenylhydrazones were converted to their respective indoles within 30 min when heated with polyphosphoric acid in a water bath at 80 °C to 95 °C. Using a direct source of heat, such as a hotplate, results in tar-like side products and sometimes red-colored side products, which can also turn into black tar upon air drying. Heating the reaction mixture in a water bath for a prolonged time also resulted in the degradation of the product, and if it was heated with distilled water without isolation and properly washed to remove acid and unreacted phenylhydrazine, the product would still degrade. It was necessary to wash the product multiple times with distilled water to remove any remaining polyphosphoric acid and then air dry it.

From this we can infer that the best way to synthesize 2-phenylbenzimidazoles from aromatic aldehydes is through microwave-assisted synthesis. Other methods reported in the literature might result in the formation of products, but the yields would not be significant. Furthermore, DMSO and DMF are not good options for the high-temperature synthesis of 2-phenylbenimidazoles and 2-phenylindoles. It can be concluded that cyclization of the benzimidazole ring specifically requires very high temperatures, and it is very difficult to reproduce most of the previously reported methods describing its synthesis at low temperatures and in mild acidic conditions. In the case of Fischer indole synthesis, cyclization/indolization initiates at temperatures of 90 °C and above, but it is exothermic in nature and can easily degrade due to excessive heat if the temperature is not maintained. Further, the reaction mixture can also self-ignite if nitro-substituted reactants are used.

### 3.2. In Silico Studies

#### 3.2.1. ADMET Profiling

The predicted ADME profiling of 3-API-B, 4-API-B, 3-APB-B, and 4-APB-B provides useful insight into their pharmacokinetic behavior and helps identify structural features that may influence their development potential. Overall, the compounds were predicted to exhibit a favorable gastrointestinal absorption profile, although this was accompanied by poor aqueous solubility, extensive plasma protein binding, variable metabolic liabilities, and several toxicity issues.

As per the results of the Deep-PK server, it is predicted that indole derivatives have better oral bioavailability than benzimidazole derivatives. Overall, the distribution, metabolism and elimination profiles of all four synthesized compounds support their development as drug candidates.

##### Toxicological Considerations

The toxicity predictions represent the limitation of the current study. The compounds are predicted to have low carcinogenicity risk, no bioconcentration potential, safe biodegradation profiles, and low environmental toxicity, but some toxicity alerts were identified. One of the greatest concerns is that all four compounds are classified as potential hERG blockers by the Deep-PK server. The hERG inhibition is associated with QT-interval prolongation and serious cardiac arrhythmias.

All four compounds are also classified as toxic for liver injury-II, but only indole derivatives were flagged for liver injury-I, indicating that benzimidazole derivatives may prove to be less toxic than indole ones.

The AMES prediction is favorable for three compounds, but 3-APB-B is predicted to be mutagenic.

Although the ADMET profiling was done by in silico prediction, it still indicates that there is a need to assess these synthesized molecules for their toxicities, and during further derivatization of these scaffolds, special consideration should be given to factors that could reduce these concerns.

#### 3.2.2. DFT Studies

The DFT computational study at the B3LYP level successfully elucidated the structural, electronic, and reactive properties of 3-API-B, 4-API-B, 3-APB-B, and 4-APB-B. Mulliken charge analysis and molecular electrostatic potential (MESP) mapping consistently identified the highly electronegative nitrogen and oxygen atoms as the primary nucleophilic centers, showcasing their high electron-donating capacity and suitability for transition metal coordination. Frontier molecular orbital (FMO) analysis revealed that the electron-donating capability decreases in the order of 3-API-B > 4-API-B > 3-APB-B > 4-APB-B. Notably, 3-API-B exhibited the narrowest HOMO–LUMO energy gap (4.15 eV), indicating lower kinetic stability, an enhanced soft character, and superior intramolecular charge transfer efficiency. These combined quantum chemical insights confirm that while all four compounds possess strong potential for intermolecular interactions, 3-API-B stands out as the most reactive framework for targeted metal complexation and therapeutic applications against biological enzyme targets. Further, it was observed that the electronic structure parameters of our synthesized compounds do not directly translate to cellular cytotoxic potency.

#### 3.2.3. Docking

Docking was performed against kinases reported in these specific cell lines, and the results indicated that all the molecules had good binding affinities. The difference in activity observed in the case of 3-APB-B and the very low IC_50_ value of 4-APB-B was not represented in the docking results.

#### 3.2.4. MD Simulation

MD simulation and its analysis indicated that these ligands bind with the target enzymes in multiple conformations. All three protein complexes preserved their overall structural compactness, secondary-structure networks, and ideal hydrogen bond geometries throughout the 100 ns trajectories.

Complex C emerges as the most structurally robust system, sustained by a highly dense, uninterrupted intermolecular hydrogen-bonding network despite localized loop flexibility. Complex B exhibited the highest degree of conformational instability, characterized by severe ligand fluctuations and a notable absence of stable hydrogen bonding over major stretches of the simulation. Complex A presents a dynamic profile wherein significant ligand reorientation occurred mid-simulation, ultimately leading to a more compact protein state and enhanced hydrogen-bonding efficiency in the final stages.

### 3.3. In Vitro Activity

All synthesized compounds were inactive against bacteria but had good cytotoxic profiles. According to the results of the in vitro cytotoxicity assay, 4-API-B was the least active agent against cancer cells but had high cytotoxicity against 3T3 cell lines. Compound 3-APB-B did show cytotoxic activity against cancer cell lines (PC3 and HeLa) but did not show much cytotoxicity against a noncancerous cell line (3T3). 4-APB-B showed a good IC_50_ value of 5.48 µM against the HeLa cell line and very weak activity against 3T3 cell lines, and its selectivity index between HeLa and 3T3 was calculated to be 10.7, indicating that 2-(4-aminophenyl)benzimidazole benzamide is a very good scaffold to further explore for its potential anticancer derivatives.

## 4. Materials and Methods

### 4.1. Synthesis and Characterization

#### 4.1.1. General Details

In the present work, analytical-grade chemicals mostly manufactured by Macklin (Shanghai, China) and Sigma Aldrich (St. Louis, MO, USA) were used. Melting points were checked via a Stuart SMP-10 melting-point apparatus (Stuart Scientific Co., Ltd., Stone, Staffordshire, UK). HF-254 TLC plates (Merck KGaA, Darmstadt, Germany) were used to check the progress of the reactions. Proton and carbon NMRs were obtained via Bruker NMR spectrophotometers of different resolutions ranging from 300 MHz to 500 MHz at 3 different facilities. In all cases, d6-DMSO was used as the solvent. Mass spectra were obtained via the ESI triple-Q technique; the instrument used was made by Agilent and was present at the ICCBS HEJ Karachi facility. The carrier used was a mixture of acetic acid and methanol, and the compounds were dissolved in acetonitrile. In vitro cytotoxicity assays and antibacterial assays were also performed by the ICCBS HEJ Karachi.

#### 4.1.2. General Procedure for Synthesis of 2-(3&4-aminophenyl)indole

Aminoacetophenone and phenylhydrazine were added to a beaker in equimolar quantities, and 20 equivalents of polyphosphoric acid was added to the same beaker. All were mixed vigorously by a glass rod and simultaneously heated in a water bath for approximately 30 min. After 30 min, the mixture was poured into ice-cold water, after which the product precipitated. The precipitates were filtered and washed with distilled water multiple times. The final product was air-dried.

#### 4.1.3. General Procedure for Synthesis of 2-(3&4-nitrophenyl)benzimidazoles

A concentrated solution of sodium bisulfite was made by dissolving excess sodium metabisulfite in 250 mL of distilled water. In this bisulfite solution, 0.033 moles of the corresponding nitrobenzaldehyde was added, and the mixture was heated over a hotplate to dissolve it by making a bisulfite adduct of aldehyde. When a clear yellowish solution was obtained, it was cooled to room temperature, and an equimolar quantity of o-phenylenediamine was added to the mixture. The mixture was intermittently irradiated in a household microwave oven for a maximum of 10 min. The solid yellow product precipitated from the mixture, which was filtered and dried in a hot-air oven at 100 °C.

#### 4.1.4. General Procedure for Synthesis of 4-aminophenylbenzimidazole

Equimolar quantities of o-phenylenediamine and para-aminobenzoic acid were added to a beaker, and 20 equivalents of polyphosphoric acid was added. It was mixed with a glass rod and then placed on a preheated hotplate at 180 °C. The mixture was stirred while heating on the hotplate; first, the mixture became clear yellow, and then it started to darken. The mixture was heated for a maximum of 20 min. The mixture was subsequently poured into ice-cold water, and the pH was neutralized with a sodium hydroxide solution. At neutral pH, precipitates appeared, which were filtered and air-dried.

#### 4.1.5. General Procedure for Reduction of 2(3&4-nitrophenyl)benzimidazoles

An amount of 0.02 moles of the corresponding 2-(nitrophenyl)benzimidazole was added to a round-bottom flask, and 30 mL of methanol and 70 mL of 37% hydrochloric acid were added. Five equivalents of tin metal beads were taken and melted on a spatula over a spirit lamp, the molten tin was then poured into distilled water to make it porous, and the porous tin was filtered and added to the reaction mixture. The reaction mixture was refluxed for 8 h and then cooled over an ice bath. Cooling resulted in the precipitation of the hydrochloride salt of the corresponding amine. If precipitates did not form, then sodium chloride was added to the mixture to facilitate the precipitation of product.

#### 4.1.6. General Procedure for Synthesis of Benzamides of 2-(3&4-aminophenyl)benzimidazoles and Indoles

An amount of 0.02 moles of the corresponding amine was placed in a round-bottom flask and dissolved in 25 mL of DMF, 0.5 equivalents of DMAP were added, and the flask was stirred over an ice bath. Excess benzoyl chloride (5 to 8 equivalents) was slowly added to the reaction mixture. The mixture was stirred in an ice bath for 24 h. Over these 24 h, the ice bath was allowed to melt and the temperature to slowly rise up to room temperature. After 24 h, 25 mL of distilled water was added to the reaction mixture, and the mixture was again stirred for 24 h at room temperature to hydrolyze the unreacted benzoyl chloride to benzoic acid. After 24 h, the reaction mixture was poured into excess water, and the precipitates were collected and washed multiple times with dilute HCl to remove DMAP and with dilute sodium carbonate solution to remove benzoic acid. The products were air-dried and packed in glass vials.

#### 4.1.7. N-[3-(1H-indol-2-yl)phenyl]benzamide (3-API-B)

Light-orange to brown powder, which decomposes above 270 °C; yield (62.2%, 3.88 g) FTIR (ATR, cm^−1^) 3056.55 (N–H amide), 1684.01 (C=O), 1589.54 (C=C), 1448.66 (C–C), 1069.90 (C–N). 1H NMR: *δ* 7.24 (m, 2H, Ar-H), 7.35 (m, 1H, Ar-H), 7.43 (q, 2H Ar-H), 7.57 (dddd, 5H, Ar-H), 7.72 (t,1H, Ar-H), 7.81 (t, 1H, Ar-H), 7.88 (q, 1H, Ar-H). 7.95 (d, 1H, Ar-H), 8.07 (d, 1H, Ar-H). 13C NMR (DMSO-*d*6, 75 MHz, *δ* ppm): 113.33 (-CH-), Ar-C {119.41, 120.44, 121.00, 121.68, 123.13, 128.15, 128.90, 129.04, 129.16, 129.35, 129.60, 129.72, 129.82, 132.24, 133.22, 134.82, 135.13, 140.25, 142.94}, 151.34 (=C-), 169.36 (C=O). ESI-MS *m*/*z:* 178.1 [M+2Na]^2+^, 399.1 [M+formic acid+2Na]^+^. C_21_H_16_N_2_O, M.W: 312.372 g/mol; Anal. Calcd: C, 80.75; H, 5.16; N, 8.97; Found: C, 81.04; H, 4.95; N, 8.92.

#### 4.1.8. N-[4-(1H-indol-2-yl)phenyl]benzamide (4-API-B)

Light-orange to brown powder that decomposes above 270 °C; yield (65.6%, 4 g) FTIR (ATR, cm^−1^) 3342.81 (C-H aromatic), 1651.23 (C=O),1597.72 (C=C) 1449.14 (C-C), 1068.95 (C−N). 1H NMR: *δ* 7.00 (t, 2H, Ar-H), 7.17 (q, 1H, Ar-H), 7.35 (q, 2H Ar-H), 7.49(m, 2H, Ar-H), 7.67 (t,1H, Ar-H), 7.89 (d, 2H, Ar-H), 7.98 (d, 2H, Ar-H). 7.95 (d, 1H, Ar-H), 8.07 (d, 1H, Ar-H). 13C NMR (DMSO-*d*6, 75 MHz, *δ* ppm): 111.65 (-CH-), Ar-C {119.60, 119.89, 120.01, 120.99, 121.77, 125.75, 128.15, 128.87, 129.20, 129.65, 129.60, 132.707, 137.39, 137.52, 138.05, 138.75, 138.98}, 154.027 (=C-), 166.01 (C=O). ESI-MS *m*/*z:* 105.1 [M+3H]^3+^, 178.1 [M+2Na]^2+^. C_21_H_16_N_2_O, M.W: 312.372 g/mol; Anal. Calcd: C, 80.75; H, 5.16; N, 8.97; Found: C, 80.47; H, 5.23; N, 9.02.

#### 4.1.9. N-[3-(1H-Benzimidazol-2-yl)phenyl]benzamide (3-APB-B)

Light-orange to brown powder, which decomposes above 270 °C; yield (74.3%, 4.65 g) FTIR (ATR, cm^−1^) 3650.16 (C-H aromatic), 3372.43 (N-H amide), 1652.30 (C=O), 1579.39 (C=C), 1448.82 (C-C), and 1071.24 (C−N). 1H NMR: *δ* 7.35 (t, 2H, Ar-H), 7.42 (t, 3H, Ar-H), 7.51 (q, 3H Ar-H), 7.59(d, 2H, Ar-H), 7.71 (d,2H, Ar-H), 7.95 (d, 1H, Ar-H). 13C NMR (DMSO-*d*6, 75 MHz, *δ* ppm): Ar-C {121.58, 123.54, 124.87, 127.57, 128.94, 129.09, 129.17, 129.22, 129.51, 129.70, 130.31, 132.90, 133.08, 134.02, 134.57, 134.93, 138.17}, 167.76 (C=O), 173.3 (=C-). ESI-MS *m*/*z:* 105.1 [M+3H]^3+^, 314.2 [M+H]^+^. C_20_H_15_N_3_O, M.W: 313.36 g/mol; Anal. Calcd: C, 76.66; H, 4.83; N, 13.41; Found: C, 76.84; H, 5.12; N, 13.62.

#### 4.1.10. N-[4-(1H-Benzimidazol-2-yl)phenyl]benzamide (4-APB-B)

C_20_H_15_N_3_O, light-orange to brown powder, which decomposes above 270 °C; yield (78.7%, 4.92 g) FTIR (ATR, cm^−1^) 3220.67 (C-H aromatic), 3058.51(N-H amide),1651.23 (C=O), 1598.45 (C=C), 1449.45 (C-C), and 1068.95 (C−N). 1H NMR: *δ* 7.16 (d, 2H, Ar-H), 7.55 (dd, 6H, Ar-H), 8.02 (t, 4H, Ar-H), 8.19 (d,2H, Ar-H). 13C NMR (DMSO-*d*6, 75 MHz, *δ* ppm): Ar-C {119.04, 119.63, 121.94, 125.49, 127.45, 128.28, 128.87, 129.63, 132.18, 135.53, 141.15, 143.05}, 151.64 (=C-), 166.23 (C=O). ESI-MS *m*/*z:* 105.1 [M+3H]^3+^, 178.2 [M+ACN+2H]^2+^, 314.2 [M+H]^+^. M.W: 313.36 g/mol; Anal. Calcd: C, 76.66; H, 4.83; N, 13.41; Found: C, 76.54; H, 5.04; N, 13.42.

### 4.2. Antimicrobial Assay

An antimicrobial assay of the synthesized compounds was performed against *Escherichia coli* (ATCC 25922), *Bacillus subtilis* (ATCC 23857), *Staphylococcus aureus* (NCTC 13277) and *Salmonella typhi* (ATCC 14028). The method used was the Microplate Alamar Blue Assay (MABA) [29]. The concentration of the compounds used was 200 mcg/mL, and ofloxacin was used as a reference at a concentration of 50 mcg/mL. The activity was performed at the ICCBS (HEJ, University of Karachi) on a commercial basis. The resulting sheets are attached in the Supplementary Materials.

### 4.3. Cytotoxicity Assay

The cytotoxic effects of the synthesized compounds were assessed against HeLa (CCL-2), PC3 (CRL-1435) and 3T3 (CRL-1658) cell lines by the MTT assay [20,30,31]. Doxorubicin was used as a reference. The concentrations used were 1 micromolar, 10 micromolar and 100 micromolar for all test compounds and the reference. The activity was performed by the ICCBS (HEJ, University of Karachi) on a commercial basis. The result sheets are attached in the Supplementary Materials. The ICCBS Karachi maintains a Bio-Bank for culturing cell lines. The cell lines used in this research were all commercially available ATCC cell lines.

### 4.4. ADMET Profiling

To evaluate the pharmacokinetic and toxicity profiles of the synthesized compounds, the ADMET (Absorption, Distribution, Metabolism, Excretion, and Toxicity) properties were predicted using the Deep-PK online tool, which employs neural networks and graph-based signatures to predict a wide array of parameters that predict ADMET and drug-likeliness properties. The tool assesses crucial descriptors, such as intestinal absorption, blood–brain barrier permeability, CYP450 enzyme interactions, total clearance, and various toxicity end points, including AMES toxicity, hepatotoxicity, and skin sensitization [32].

### 4.5. Hierarchical Clustering

Hierarchical clustering was done to assess the structural similarity among the synthesized compounds using a cheminformatics-based approach. The molecular structures were first drawn and optimized using ACD/ChemSketch and saved in an SDF file. The SDF file was used as input to Chemine tools, a web-based platform for chemical informatics analysis that utilizes JOELib molecular descriptors for feature extraction. Multiple descriptors, including topological, constitutional, and electronic properties, were predicted. The clustering was performed using the complete linkage method, and a dendrogram was generated by the server, which visually grouped compounds based on their structural similarities. This enabled the identification of compound families with potential structure–activity relationships, supporting the further interpretation of biological data [33].

### 4.6. DFT Studies

All the DFT calculations were performed using the Gaussian 09 program. Visualization of the output files, charge distribution analysis, frontier orbital distribution and potential energy surfaces (PESs) of 3-API-B, 4-API-B, 3-APB-B and 4-APB-B was carried out by Gauss view 09 [34]. The equilibrium geometries of all compounds were fully relaxed without any symmetry constraints by DFT methods at the B3LYP level of theory [35,36], with the correlation function [36] implementing 6-311(++) basis sets. The B3LYP level of theory is reliable for the structural, electronic and optical properties of synthetic and natural products with low computational cost and high accuracy [37]. The frontier molecular orbitals (HOMO, LUMO), band gap and molecular electrostatic potential (MEP) were also computed at the B3LYP/6-31G (d,p) level of theory to determine the charge transfer behavior of the molecule.

### 4.7. Docking Studies

The structures of the synthesized compounds were drawn in Discovery Studio Visualizer 2025 and saved in PDB format; then, they were converted into PDBQT format by MGL/AutoDock tools v.1.5.7 [38]. Protein files were downloaded from the protein data bank, cleaned in Discover Studio Visualizer 2025 and converted to PDBQT files via MGL/AutoDock tools v1.5.7. Docking was performed by AutoDock Vina version 1.2.7 [39,40]. Docking images were obtained via ChimeraX version 1.8, Discovery Studio Visualizer 2025 and PyMOLv.3.1.0 [41].

### 4.8. Molecular Dynamic Studies

Ligands that had shown the highest binding affinities in docking with three proteins (3-API-B-6N7A, 4-API-B-3IW4 and 4-API-B-7DXL) were subjected to 100 ns all-atom MD simulations for further assessment of the binding affinities. Charmm-GUI [42] was utilized for the preparation of the MD simulation files. The system energy was computed, and the water box size was set using a point water model (TIP3P) with an edge distance of 10.0Å. Ions were placed via the Monte Carlo method. Sodium chloride (NaCl) was chosen as the basic ion type. The system size and dimensions were determined using the solvator module of CHARMM-GUI. The simulation box was defined by a rectangular shape, cubic crystal type, and crystal angles. Periodic boundary conditions were applied to facilitate the generation of grid data for the Particle Mesh Ewald (PME) and Fast Fourier Transform (FFT) algorithms [43] using the CHARMM36m force field [44].

Equilibration and production run input files were generated using the NVT (constant number of particles, volume, and temperature) and NPT (constant number of particles, pressure, and temperature) ensembles, respectively, with the temperature set to a default value of 303.15 K. Subsequently, a 100 ns all-atom molecular dynamic (MD) simulation was carried out using GROMACS 5.1 [45]. Snapshots were saved at every 10 ps time point. Trajectory analysis was performed using GROMACS built-in tools to compute the Root-Mean-Square Deviation (RMSD) and Root-Mean-Square Fluctuation (RMSF). Additionally, the system’s potential energy and radius of gyration were calculated. GRACE 5.1.25 was used to plot the graphs, while VMD and PyMOL 2.5.4 [41] were employed for trajectory visualization and complex figure generation, respectively.

Hydrogen bonds between ligand–protein complexes were evaluated using the gmx hbond tool. Standard pre-set values of the hydrogen bond angle and distance cutoff were used. Plots of the number of hydrogen bonds as a function time, of the angular distribution of all hydrogen bonds and of the distance distribution of all hydrogen bonds were generated using the gmx hbond tool.

## 5. Conclusions

In this study, we explored ways to synthesize 2-(4&3-aminophenyl)-indoles and benzimidazoles, and based on the results of in vitro cytotoxic assays, it can be concluded that 4-APB-B and 3-APB-B are very good scaffolds for potential kinase inhibitors. Compound 2-(4-aminophenyl)benzimidazole benzamide (4-APB-B) especially needs to be evaluated for its safety in animal models due its predicted toxicity and high potential as a scaffold for future drug development.

Regarding the synthesis of indoles, we suggest that further studies are still needed to explore Fischer indole synthesis, the effect of hydrazone stability on indolization, and, specifically, the synthesis of 2-(nitrophenyl)indoles directly from nitroacetophenones.

## Figures and Tables

**Table 1:** Synthesized benzamides of 2-(aminophenyl)indoles and benzimidazoles.

S. No	Code	Structure	Yield (%)
1	3-API-B	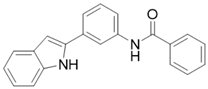	62.2%
2	4-API-B	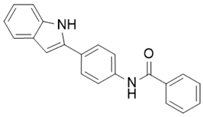	65.6%
3	3-APB-B	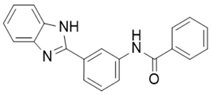	74.3%
4	4-APB-B	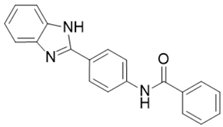	78.7%

**Table 2:** Predicted general physicochemical properties of synthesized compounds, including boiling points (BPs) (°C), hydration free energies (kJ/mol), Log (D) values at pH 7.4, Log (P) values, aqueous solubilities (Log (S)), melting points (MPs) (°C), and predicted pKa values (acidic and basic).

Compound	BP (°C)	Hydration Free Energy	Log (D) at pH = 7.4	Log (P)	Log (S)	MP (°C)	pKa Acid	pKa Basic
3-APB-B	504.81	−9.06	3.25	4.06	−5.78	253.92	13.48	4.67
3-API-B	474.36	−9.92	4.42	4.53	−6.82	240.74	10.33	4.53
4-APB-B	504.39	−9.02	3.36	3.97	−5.62	265.43	13.34	5.16
4-API-B	475.22	−9.92	4.43	4.5	−6.86	256.74	10.53	4.66

**Table 3:** Predicted absorption-related pharmacokinetic parameters of synthesized compounds using Deep-PK model.

Compound	Caco-2 (log Paap)	Human Oral Bioavailability 20% (Confidence)	Human Intestinal Absorption (Confidence)	Madin–Darby Canine Kidney	Human Oral Bioavailability 50% (Confidence)	P-Glycoprotein Inhibitor	P-Glycoprotein Substrate (Confidence)	Skin Permeability (log K_p_)
3-APB-B	−5.12	0.551 (Low)	0.957 (High)	−4.69	0.573 (Low)	Inhibitor	No (0.44) (Low)	−0.29
3-API-B	−4.89	0.767 (Medium)	0.998 (High)	−4.68	0.499 (Low)	Non-Inhibitor	No (0.204) (Medium)	−1.63
4-APB-B	−5.07	0.595 (Low)	0.963 (High)	−4.68	0.595 (Low)	Inhibitor	No (0.422) (Low)	−0.76
4-API-B	−4.9	0.778 (Medium)	0.998 (High)	−4.64	0.503 (Low)	Non-Inhibitor	No (0.201) (Medium)	−1.67

**Table 4:** Predicted distribution properties of synthesized compounds.

Compound	Blood–Brain Barrier	Unbound Fraction (Human)	Plasma Protein Binding	Steady-State Volume of Distribution (L/kg)
3-APB-B	Non-Penetrable (High Confidence)	None	91.14	1.92
3-API-B	Penetrable (Medium Confidence)	None	85.73	1.66
4-APB-B	Non-Penetrable (High Confidence)	None	89.35	2.02
4-API-B	Penetrable (High Confidence)	None	84.34	1.73

**Table 5:** Predicted metabolic profiles of synthesized compounds by Deep-PK server.

Compound		3-APB-B	3-API-B	4-APB-B	4-API-B
Breast Cancer Resistance Protein		Non-Inhibitor (High Confidence)	Inhibitor (High Confidence)	Non-Inhibitor (High Confidence)	Inhibitor (High Confidence)
CYP 1A2	Inhibitor	Inhibitor (High Confidence)	Inhibitor (High Confidence)	Non-Inhibitor (Low Confidence)	Inhibitor (Medium Confidence)
	Substrate	Non-Substrate (Low Confidence)	Substrate (Low Confidence)	Non-Substrate (Low Confidence)	Substrate (Low Confidence)
CYP 2C19	Inhibitor	Inhibitor (High Confidence)	Inhibitor (High Confidence)	Inhibitor (High Confidence)	Inhibitor (High Confidence)
	Substrate	Non-Substrate (Medium Confidence)	Substrate (Low Confidence)	Non-Substrate (Medium Confidence)	Substrate (Low Confidence)
CYP 2C9	Inhibitor	Inhibitor (High Confidence)	Inhibitor (Low Confidence)	Inhibitor (Medium Confidence)	Non-Inhibitor (High Confidence)
	Substrate	Non-Substrate (High Confidence)	Substrate (Low Confidence)	Non-Substrate (High Confidence)	Substrate (Low Confidence)
CYP 2D6	Inhibitor	Non-Inhibitor (High Confidence)	Inhibitor (Medium Confidence)	Non-Inhibitor (High Confidence)	Inhibitor (Medium Confidence)
	Substrate	Non-Substrate (Low Confidence)	Substrate (Low Confidence)	Non-Substrate (Low Confidence)	Substrate (Low Confidence)
CYP 3A4	Inhibitor	Inhibitor (Medium Confidence)	Inhibitor (Medium Confidence)	Non-Inhibitor (Low Confidence)	Inhibitor (Low Confidence)
	Substrate	Non-Substrate (Medium Confidence)	Non-Substrate (Medium Confidence)	Non-Substrate (Medium Confidence)	Non-Substrate (Medium Confidence)
OATP1B1		Non-Inhibitor (High Confidence)	Non-Inhibitor (Medium Confidence)	Non-Inhibitor (High Confidence)	Non-Inhibitor (Medium Confidence)
OATP1B3		Non-Inhibitor (Medium Confidence)	Non-Inhibitor (High Confidence)	Non-Inhibitor (Medium Confidence)	Non-Inhibitor (High Confidence)

CYP—Cytochrome P450; OATP—Organic Anion Transporting Polypeptide.

**Table 6:** Predicted elimination profiles of synthesized compounds.

Compound	Predicted Clearance (mL/min/kg)	Organic Cation Transporter 2	Predicted Half-Life
3-APB-B	3.85	Non-Inhibitor	<3 hs
3-API-B	6.83	Non-Inhibitor	<3 hs
4-APB-B	3.83	Non-Inhibitor	<3 hs
4-API-B	6.82	Non-Inhibitor	<3 hs

**Table 7:** Toxicological predictions for synthesized compounds.

Parameter	Compound			
	3-APB-B	3-API-B	4-APB-B	4-API-B
AMES Mutagenesis	Toxic	Safe	Safe	Safe
Avian	Safe	Safe	Safe	Safe
Bee	Safe	Safe	Safe	Safe
Bioconcentration Factor	None	None	None	None
Biodegradation	Safe	Safe	Safe	Safe
Carcinogenesis	Safe	Safe	Safe	Safe
Crustacean	Toxic	Toxic	Toxic	Toxic
Liver Injury-I (DILI)	Safe	Toxic	Safe	Toxic
Eye Corrosion	Safe	Safe	Safe	Safe
Eye Irritation	Safe	Toxic	Safe	Toxic
Liver Injury-II	Toxic	Toxic	Toxic	Toxic
hERG Blockers	Toxic	Toxic	Toxic	Toxic
*Daphnia magna*	None	None	None	None
Micronucleus	Toxic	Toxic	Toxic	Toxic
NR-AhR	Toxic	Toxic	Toxic	Toxic
NR-AR	Safe	Safe	Safe	Safe
NR-AR-LBD	Safe	Safe	Safe	Safe
NR-Aromatase	Safe	Safe	Safe	Safe
NR-ER	Safe	Toxic	Safe	Toxic
NR-ER-LBD	Safe	Safe	Safe	Safe
NR-GR	Toxic	Safe	Toxic	Safe
NR-PPAR-gamma	Safe	Safe	Safe	Safe
NR-TR	Toxic	Safe	Toxic	Safe
T. Pyriformis	−10.95	−0.52	−9.39	−0.44
Rat (Acute)	2.59	2.13	2.57	2.16
Rat (Chronic Oral)	2.15	1.95	2.27	1.92
Fathead Minnow	6.07	4.86	5.88	4.84
Respiratory Disease	Safe	Safe	Safe	Safe
Skin Sensitization	Toxic	Toxic	Toxic	Toxic
SR-ARE	Safe	Toxic	Safe	Toxic
SR-ATAD5	Safe	Safe	Safe	Safe
SR-HSE	Safe	Safe	Safe	Safe
SR-MMP	Toxic	Toxic	Toxic	Toxic
SR-p53	Safe	Safe	Safe	Safe

**Table 8:** Frontier molecular orbital energies calculated using DFT. Respective energies of highest occupied molecular orbital (*E_HOMO_*), lowest unoccupied molecular orbital (*E_LUMO_*) and gap (Δ*E*) in between.

	*E*_HOMO_ (eV)	*E*_LUMO_ (eV)	Δ*E* (eV)
3-API-B	−4.88 (paired)	−0.725	4.15
4-API-B	−5.50 (paired)	−1.220	4.28
3-APB-B	−5.77 (paired)	−1.398	4.37
4-APB-B	−5.85 (paired)	−1.461	4.39

**Table 9:** Binding affinity of highest ranking pose of each synthesized and co-crystallized ligand against kinases in Kcal/mol.

	3-API-B	4-API-B	3-APB-B	4-APB-B	Co-Crystalized Ligand
PKC-α PDB ID 3IW4	−9.142	−9.763	−8.891	−9.63	−11.74
JAK1 Kinase PDB ID 6N7A	−9.352	−8.989	−9.28	−8.859	−8.671
AXL Kinase PDB ID 7DXL	−7.784	−8.074	−7.791	−7.916	−8.661

**Table 10:** Cytotoxic effects (IC_50_ in µM with SD) of synthesized compounds against HeLa, PC3 and 3T3 cell lines.

	HeLa	PC3	3T3	Selectivity Index (SI)
3-API-B	61.07 ± 0.27	45.52 ±1.2	65.21 ± 0.06	1.4 (PC3 vs. 3T3)
4-API-B	--	--	71.91 ± 0.01	--
3-APB-B	23.97 ± 0.17	33.81 ± 0.33	--	--
4-APB-B	5.48 ± 0.01	17.7 ± 0.22	58.64 ± 0.03	10.7 (HeLa vs. 3T3)
Doxorubicin	0.86 ± 0.12	2.12 ± 0.2	0.05 ± 0.04	0.058 (HeLa vs. 3T3)

## Data Availability

The original contributions presented in this study are included in the article/Supplementary Materials. Further inquiries can be directed to the corresponding author.

## Supplementary Materials

The following supporting information can be downloaded at: https://www.mdpi.com/article/10.3390/ph19091485/s1, FTIR Specs; HEJ assay Results 1st 4 compounds; MASS SPECS 4 COMPOUNDS; NMR of metal complex; Raw NMR spectra; Spectra with structures.

## Abbreviations

The following abbreviations are used in this manuscript:

PABA	Para-amino benzoic acid
3-API	2-(3-aminophenyl)indole
4-API	2-(4-aminophenyl)indole
3-APB	2-(3-aminophenyl)benzimidazole
4-APB	2-(4-aminophenyl)benzimidazole
3-API-B	Benzamide of 3-API
4-API-B	Benzamide of 4-API
3-APB-B	Benzamide of 3-APB
4-APB-B	Benzamide of 4-APB

## References

[1] Anderson B., Rosston P., Ong H.W., Hossain M.A., Davis-Gilbert Z.W., Drewry D.H. (2023). How many kinases are druggable? A review of our current understanding. Biochem. J..

[2] Drăgoi C.M., Nicolae A.-C., Dumitrescu I.-B. (2026). The indole scaffold in biochemistry and therapeutics: A privileged structure with diverse chemical, biological, and clinical significance. Targets.

[3] Zhang H., He F., Gao G., Lu S., Wei Q., Hu H., Wu Z., Fang M., Wang X. (2023). Approved small-molecule ATP-competitive kinases drugs containing indole/azaindole/oxindole scaffolds: R&D and binding patterns profiling. Molecules.

[4] Aziz H.A., El-Saghier A.M., Badr M., Elsadek B.E.M., Abuo-Rahma G.E.-D.A., Shoman M.E. (2024). Design, synthesis and mechanistic study of N-4-Piperazinyl Butyryl Thiazolidinedione derivatives of ciprofloxacin with Anticancer Activity via Topoisomerase I/II inhibition. Sci. Rep..

[5] Mahurkar N.D., Gawhale N.D., Lokhande M.N., Uke S.J., Kodape M.M. (2023). Benzimidazole: A versatile scaffold for drug discovery and beyond—A comprehensive review of synthetic approaches and recent advancements in medicinal chemistry. Results Chem..

[6] Barot K.P., Nikolova S., Ivanov I., Ghate M.D. (2013). Novel research strategies of benzimidazole derivatives: A review. Mini Rev. Med. Chem..

[7] Lee Y.T., Tan Y.J., Oon C.E. (2023). Benzimidazole and its derivatives as cancer therapeutics: The potential role from traditional to precision medicine. Acta Pharm. Sin. B.

[8] Robinson B. (1969). Studies on the Fischer indole synthesis. Chem. Rev..

[9] Humphrey G.R., Kuethe J.T. (2006). Practical methodologies for the synthesis of indoles. Chem. Rev..

[10] Gaikwad R., Bobde Y., Ganesh R., Patel T., Rathore A., Ghosh B., Das K., Gayen S. (2019). 2-Phenylindole derivatives as anticancer agents: Synthesis and screening against murine melanoma, human lung and breast cancer cell lines. Synth. Commun..

[11] Joule J.A., Mills K. (2008). Heterocyclic Chemistry.

[12] Sajjadifar S., Vahedi H., Massoudi A., Louie O. (2010). New 3 H-Indole Synthesis by Fischer’s Method. Part I. Molecules.

[13] Panda S.S., Malik R., Jain S.C. (2012). Synthetic approaches to 2-arylbenzimidazoles: A review. Curr. Org. Chem..

[14] Alheety N.F., Awad S.A., Alheety M.A., Darwesh M.Y., Abbas J.A., Besbes R. (2026). Benzimidazole Derivatives: A Review of Advances in Synthesis, Biological Potential, Computational Modelling, and Specialized Material Functions. Chemistry.

[15] Abdul Rahim A.S., Muhamad Salhimi S., Arumugam N., Chung Pin L., Shy Yee N., Muttiah N.N., Boon Keat W., Hamid S.A., Osman H., Mat I.B. (2013). Microwave-assisted synthesis of sec/tert-butyl 2-arylbenzimidazoles and their unexpected antiproliferative activity towards ER negative breast cancer cells. J. Enzym. Inhib. Med. Chem..

[16] Furniss B.S. (2004). Practical Organic Chemistry.

[17] Yang T., Shi X., Kang Y., Zhu M., Fan M., Zhang D., Zhang Y. (2018). Novel compounds TAD-1822-7-F2 and F5 inhibited HeLa cells growth through the JAK/Stat signaling pathway. Biomed. Pharmacother..

[18] Chiu C.-L., Zhang D., Zhao H., Wei Y., Polasko A.L., Thomsen M.T., Yang V., Yang K.K., Hauck S., Peterson E.E. (2025). Targeting AXL inhibits the growth and metastasis of prostate cancer in bone. Clin. Cancer Res..

[19] Überall F., Giselbrecht S., Hellbert K., Fresser F., Bauer B., Gschwendt M., Grunicke H.H., Baier G. (1997). Conventional PKC-α, novel PKC-ε and PKC-θ, but not atypical PKC-λ are MARCKS kinases in intact NIH 3T3 fibroblasts. J. Biol. Chem..

[20] Supino R. (1995). MTT assays. In Vitro Toxicity Testing Protocols.

[21] Ottoni O., Neder A.d.V., Dias A.K., Cruz R.P., Aquino L.B. (2001). Acylation of indole under friedel–crafts conditions an improved method to obtain 3-acylindoles regioselectively. Org. Lett..

[22] Inman M., Moody C.J. (2013). Indole synthesis–something old, something new. Chem. Sci..

[23] Lan X., Wang C., Tian F., Qiu M., Bai G. (2015). Room temperature synthesis of 2-benzimidazoles with hydrogen peroxide as oxidant and supported ammonium molybdate as catalyst. Res. Chem. Intermed..

[24] Surpur M.P., Singh P.R., Patil S.B., Samant S.D. (2007). One-pot synthesis of benzimidazoles from o-nitroanilines under microwaves via a reductive cyclization. Synth. Commun..

[25] Vogel A.I., Furniss B.S. (1989). Vogel’s Textbook of Practical Organic Chemistry.

[26] Ponomarev I., Rybkin Y.Y., Volkova Y.A., Razorenov D.Y., Skupov K., Ponomarev I.I., Senchukova A., Lezov A., Tsvetkov N. (2020). New possibilities for the synthesis of high-molecular weight poly (2,5(6)-benzimidazole) and studies of its solutions in DMSO-based complex organic solvent. Russ. Chem. Bull..

[27] Shinde R.B., Pansare D.N., Shelke R.N., Sarkate A.P., Tiwari S.V., Bangal M.N., Bhagat D.S., Zine A.M. (2023). A facile synthesis and characterization of some novel benzimidazole derivatives. Results Chem..

[28] Hothersall A., Clarke S., Macnaughtan D.J. (1934). The electrodeposition of tin from sodium stannate solutions with the use of insoluble anodes. Trans. IMF.

[29] Pettit R.K., Weber C.A., Kean M.J., Hoffmann H., Pettit G.R., Tan R., Franks K.S., Horton M.L. (2005). Microplate Alamar blue assay for Staphylococcus epidermidis biofilm susceptibility testing. Antimicrob. Agents Chemother..

[30] El-Saghier A.M., Hamed A.M., Abosella L., Bräse S., Aziz H.A. (2026). Novel N-4-(5-Amino-7-substituted-triazolotriazinepiperazin-1-yl) Norfloxacin Analogues Exhibit Potent and Selective Anticancer Activity via Topoisomerase Inhibition, Cell-Cycle Arrest, and Apoptosis in A 431 Skin Carcinoma Cells. Pharmaceuticals.

[31] Abd El-hameed A.I., Salem I.M., Allam R.M., Arafat M.M., Aziz H.A., Awan Z.A., Ibrahim T.S., Alshazly O., Beshr E.A.M., Bräse S. (2026). Exploration of novel hydroxamic acid-based candidates integrating chalcone scaffold cap as multitarget HDAC inhibitors: Design, anti-prostatic cancer assessment, in silico docking and molecular dynamic simulation. J. Enzym. Inhib. Med. Chem..

[32] Myung Y., de Sá A.G., Ascher D.B. (2024). Deep-PK: Deep learning for small molecule pharmacokinetic and toxicity prediction. Nucleic Acids Res..

[33] Backman T.W., Cao Y., Girke T. (2011). ChemMine tools: An online service for analyzing and clustering small molecules. Nucleic Acids Res..

[34] Frisch M., Trucks G., Schlegel H., Scuseria G., Robb M., Cheeseman J., Scalmani G., Barone V., Mennucci B., Petersson G. (2009). Gaussian 09.

[35] Becke A.D. (1993). Density-functional thermochemistry. III. The role of exact exchange. J. Chem. Phys..

[36] Lee C., Yang W., Parr R.G. (1988). Development of the Colle-Salvetti correlation-energy formula into a functional of the electron density. Phys. Rev. B.

[37] Ahmed M.N., Yasin K.A., Ayub K., Mahmood T., Tahir M.N., Khan B.A., Hafeez M., Ahmed M. (2016). Click one pot synthesis, spectral analyses, crystal structures, DFT studies and brine shrimp cytotoxicity assay of two newly synthesized 1,4,5-trisubstituted 1,2,3-triazoles. J. Mol. Struct..

[38] Pacheco A.B., Hpc L. (2012). Introduction to AutoDock and AutoDock Tools.

[39] Eberhardt J., Santos-Martins D., Tillack A.F., Forli S. (2021). AutoDock Vina 1.2. 0: New docking methods, expanded force field, and python bindings. J. Chem. Inf. Model..

[40] Trott O., Olson A. (2009). Software news and update AutoDock Vina: Improving the speed and accuracy of docking with a new scoring function. Effic. Optim. Multithreading.

[41] DeLano W.L. (2002). Pymol: An open-source molecular graphics tool. CCP4 Newsl. Protein Crystallogr..

[42] Jo S., Kim T., Iyer V.G., Im W. (2008). CHARMM-GUI: A web-based graphical user interface for CHARMM. J. Comput. Chem..

[43] Ross G.A., Rustenburg A.S., Grinaway P.B., Fass J., Chodera J.D. (2018). Biomolecular simulations under realistic macroscopic salt conditions. J. Phys. Chem. B.

[44] Brooks B.R., Brooks C.L., Mackerell A.D., Nilsson L., Petrella R.J., Roux B., Won Y., Archontis G., Bartels C., Boresch S. (2009). CHARMM: The biomolecular simulation program. J. Comput. Chem..

[45] Lee J., Cheng X., Swails J.M., Yeom M.S., Eastman P.K., Lemkul J.A., Wei S., Buckner J., Jeong J.C., Qi Y. (2016). CHARMM-GUI input generator for NAMD, GROMACS, AMBER, OpenMM, and CHARMM/OpenMM simulations using the CHARMM36 additive force field. J. Chem. Theory Comput..

