# Supplementary material for: Benzamides of 2-(aminophenyl)benzimidazoles and 2-(aminophenyl)indoles as Anticancer Scaffolds: Synthesis, In Silico and In Vitro Evaluation
Source: Pharmaceuticals (Basel). 2026 Sep 17;19(9):1485. doi: 10.3390/ph19091485 (PMC13610037; doi:10.3390/ph19091485)
Supplement: Supplementary file 1 [file pharmaceuticals-19-01485-s001.zip › Spectra with structures.pdf]

Benzamides of 2-(aminophenyl)benzimidazoles and 2-(aminophenyl)indoles as anticancer scaffolds, Synthesis, *In Silico* and *In Vitro* Evaluation

Adil Saeed [1,*], Humaira Nadeem [2], Fouzia Parveen Malik [3], Rehan Zafar Paracha [4] and Sehrosh Naz Khan [5].

[1]Supplementary Data:

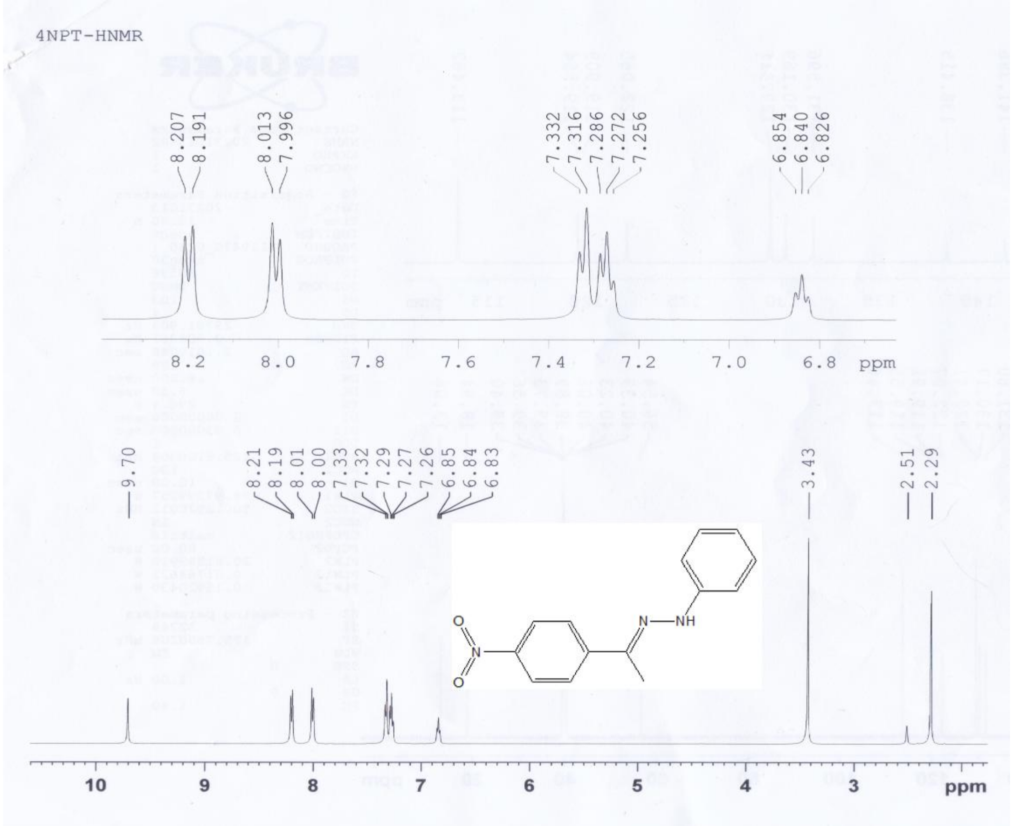

Figure S1. 1H-NMR of 4-nitroacetophenone phenylhydrazone

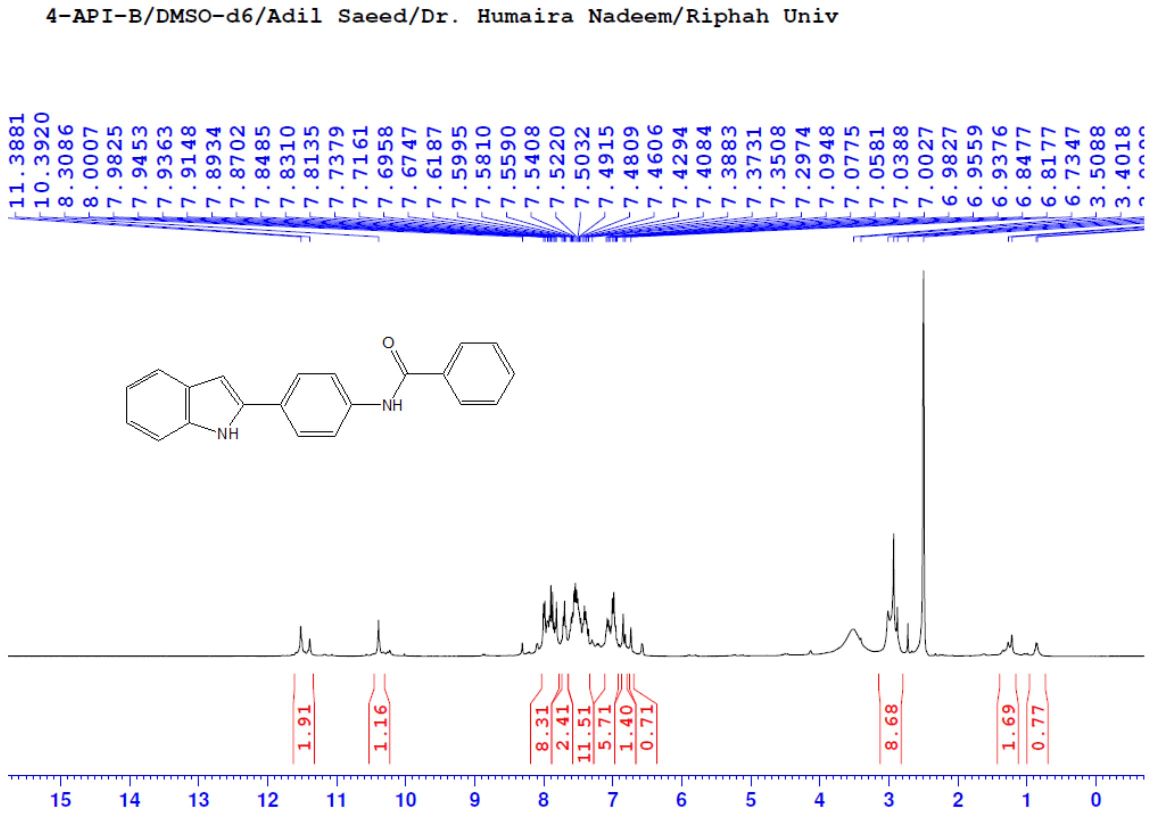

Figure S2. 1H NMR of 2-(4-aminophenyl)indole Benzamide

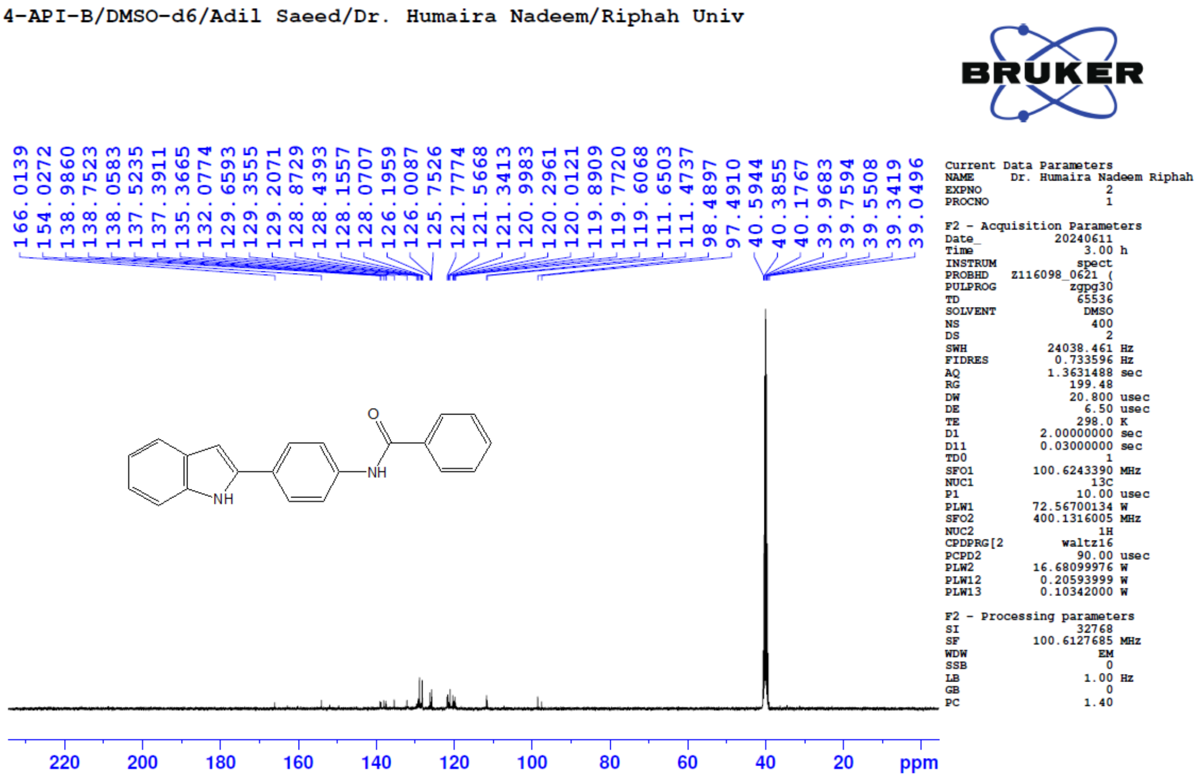

Figure S3. C13 NMR of 2-(4-aminophenyl)indole benzamide

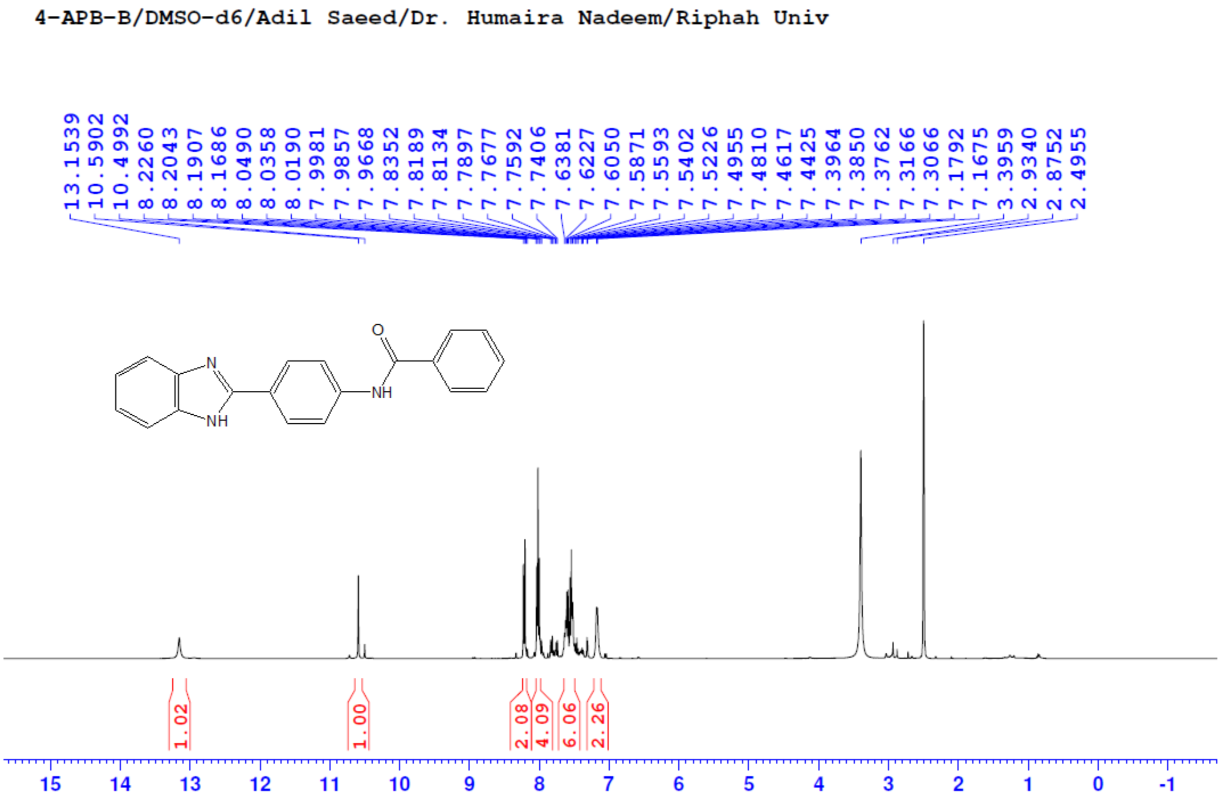

Figure S4. 1H NMR of 2-(4-aminophenyl)benzimidazole benzamide

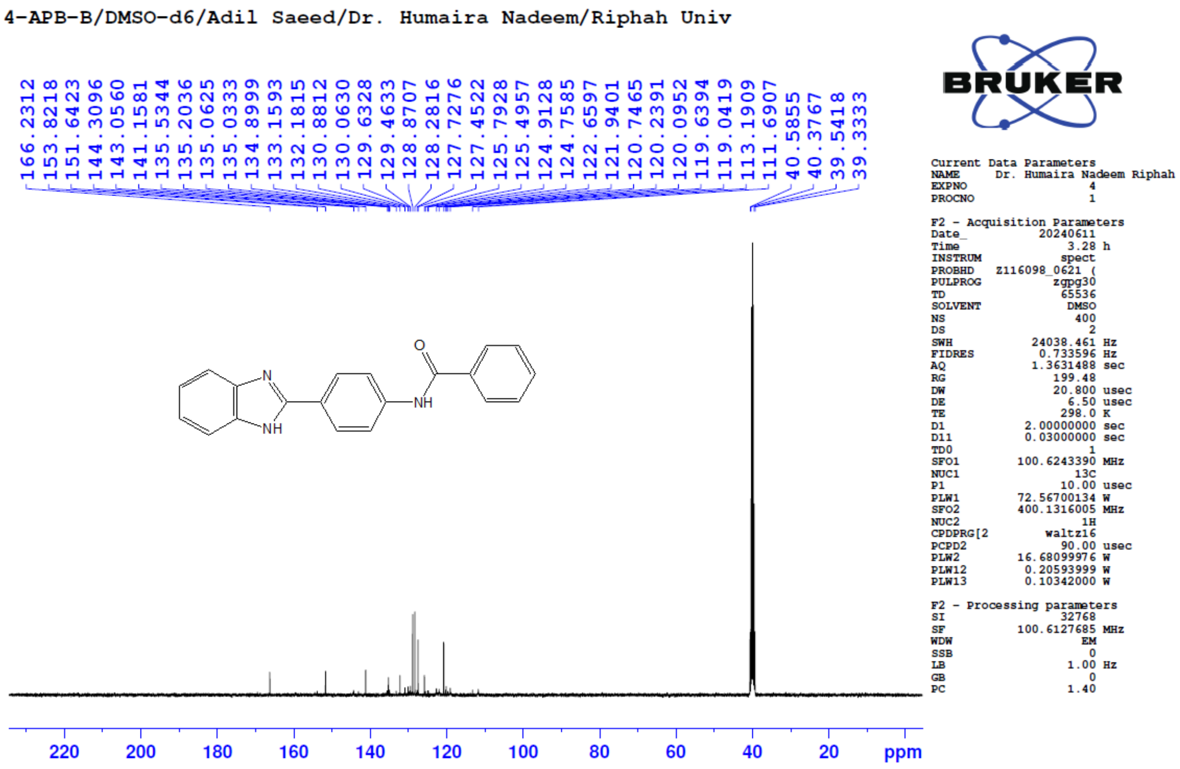

Figure S5. C13 NMR of 2-(4-aminophenyl)benzimidazole benzamide

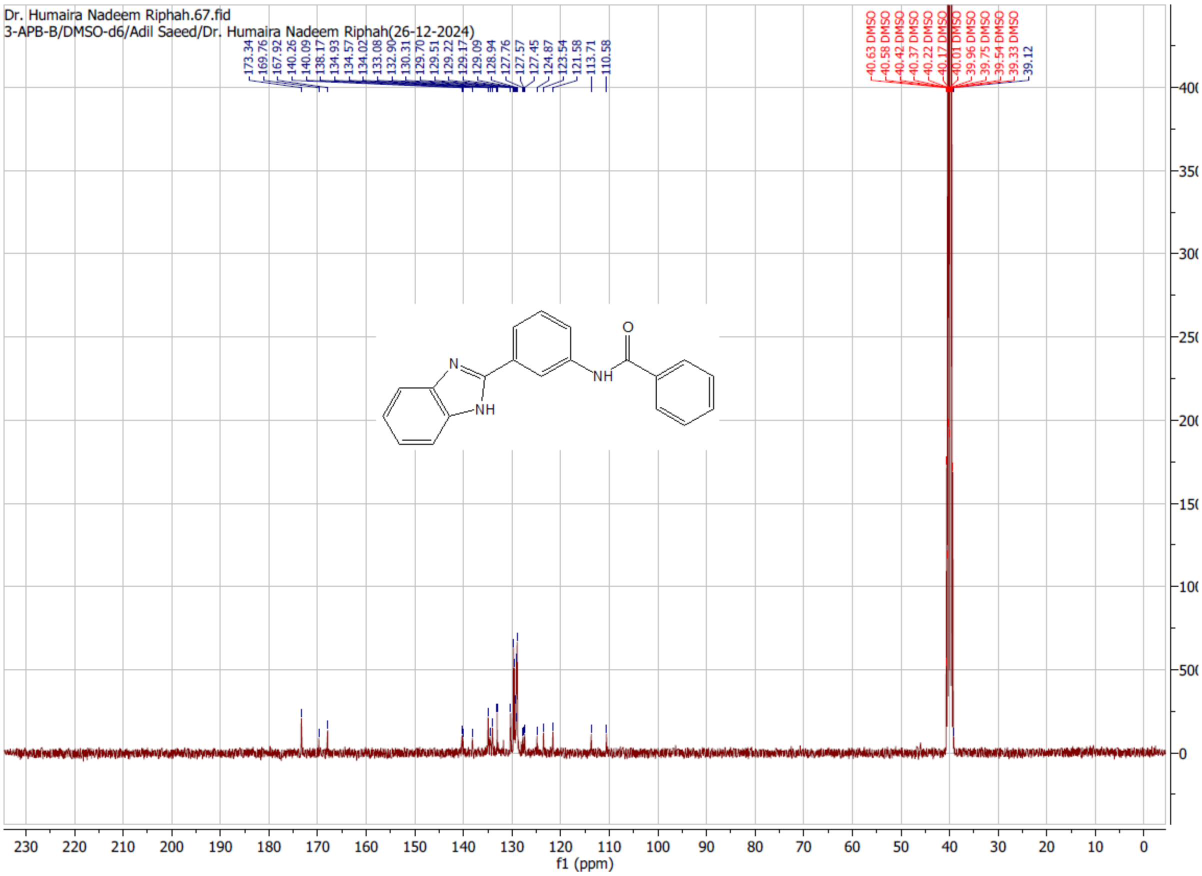

Figure S6. C13 NMR of 2-(3-aminophenyl)benzimidazole benzamide

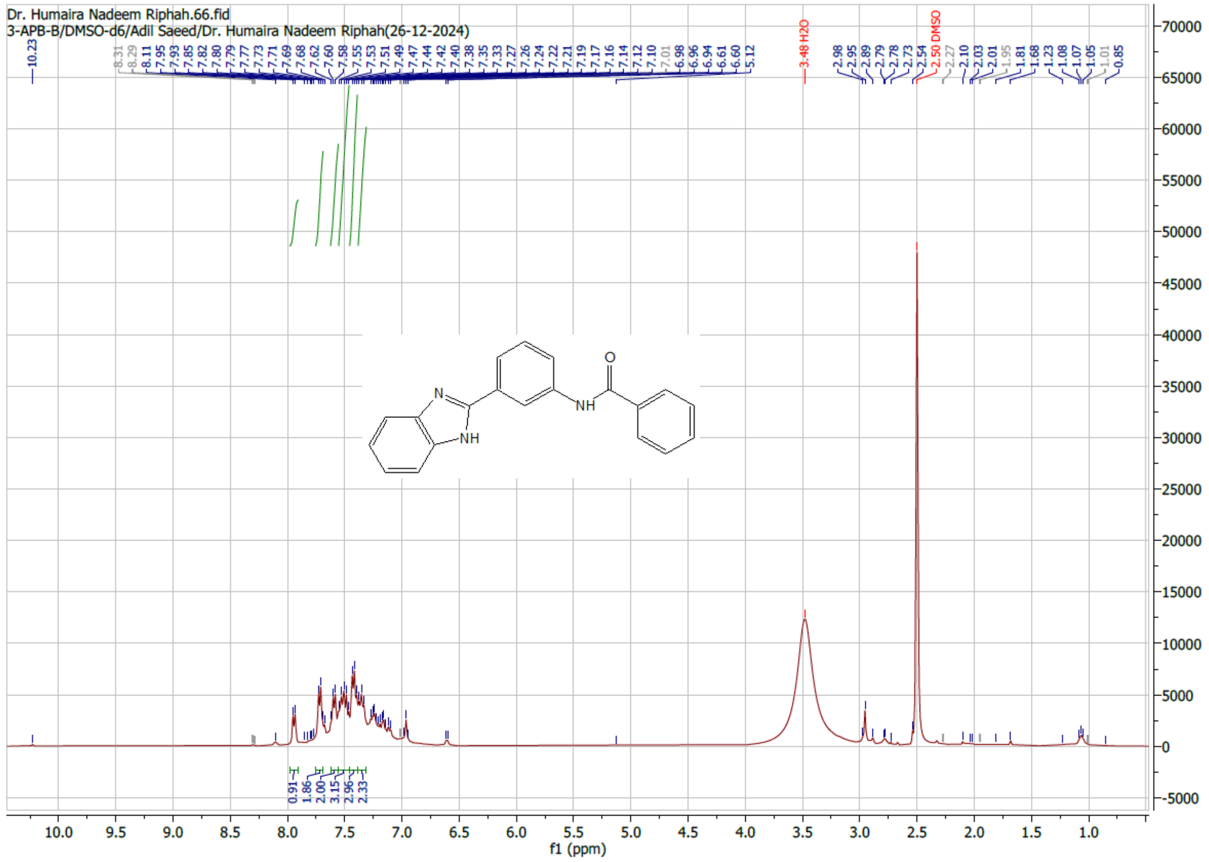

Figure S7. 1H NMR of of 2-(3-aminophenyl)benzimidazole benzamide

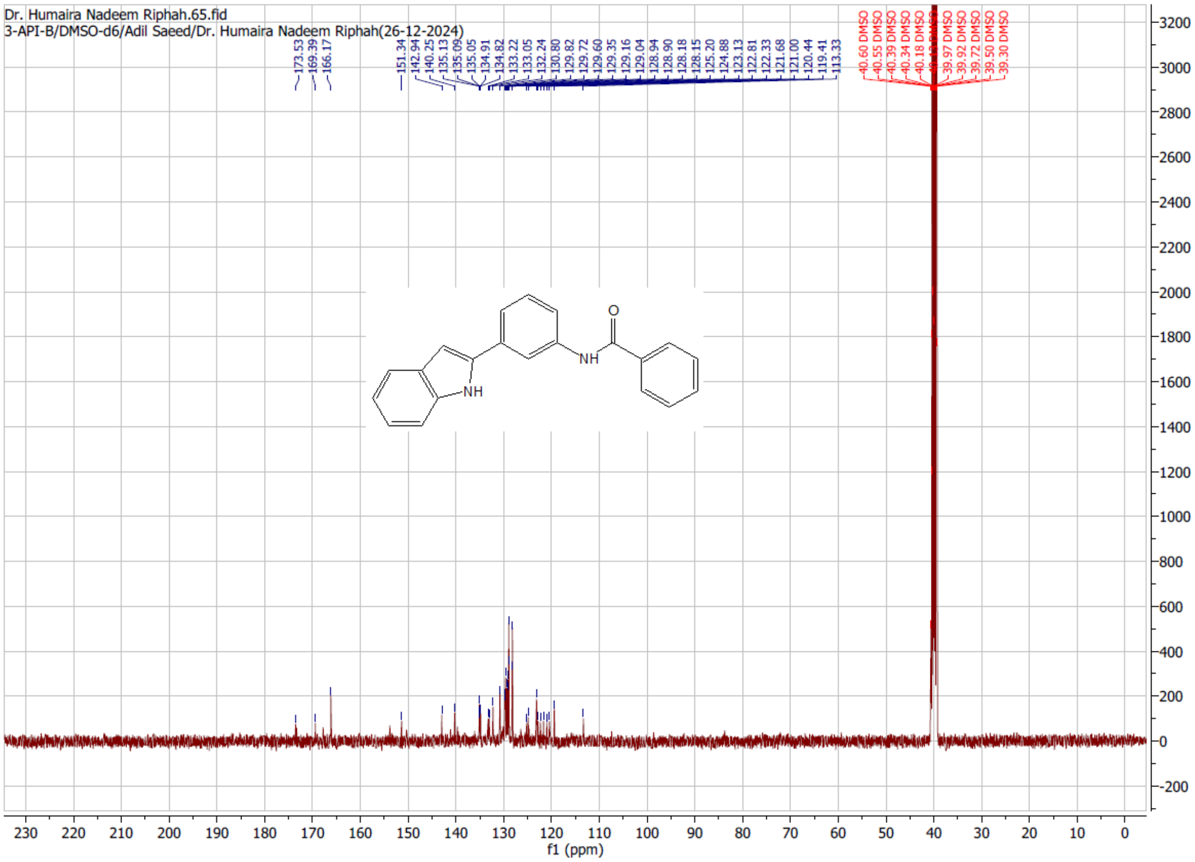

Figure S8. C13 NMR of 2-(3-aminophenyl)indole benzamide

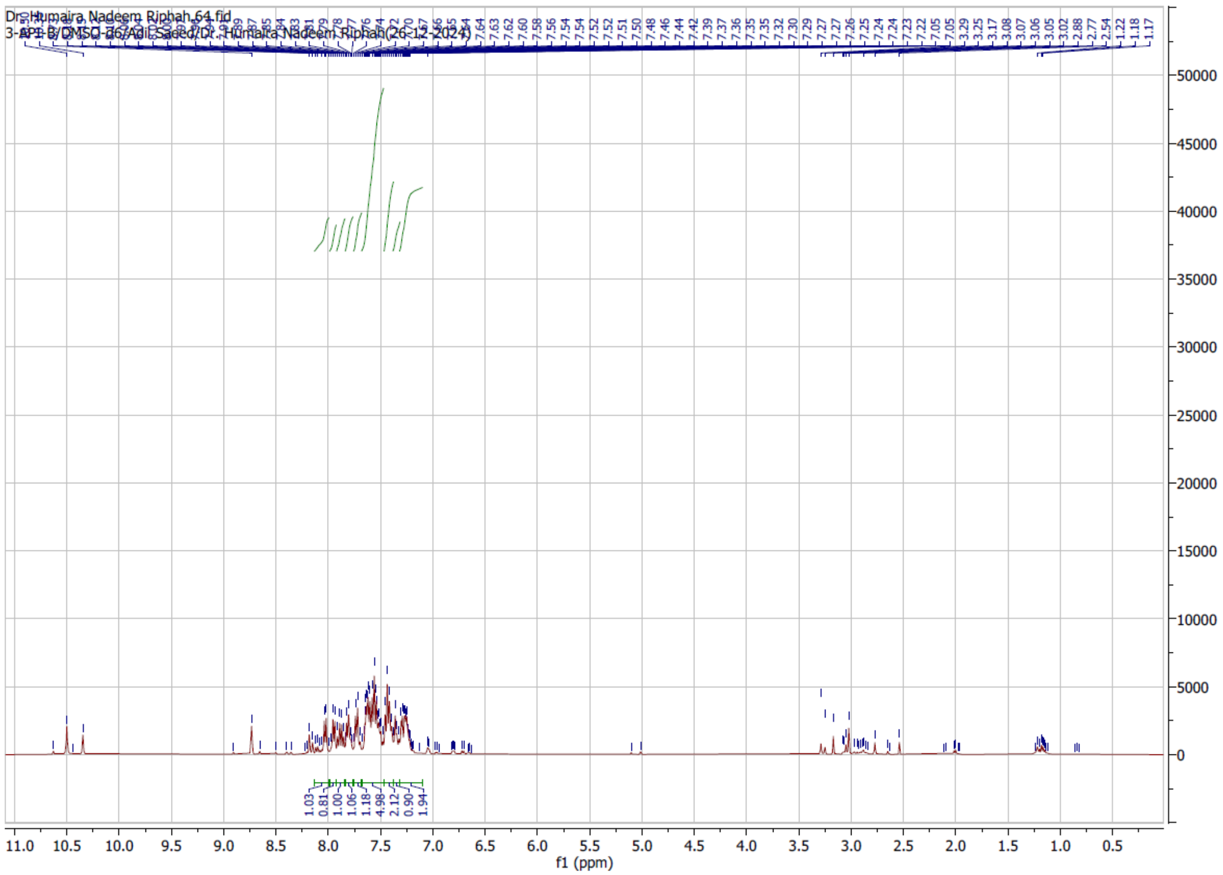

Figure S9. 1H NMR of 2-(3-aminophenyl)indole benzamide

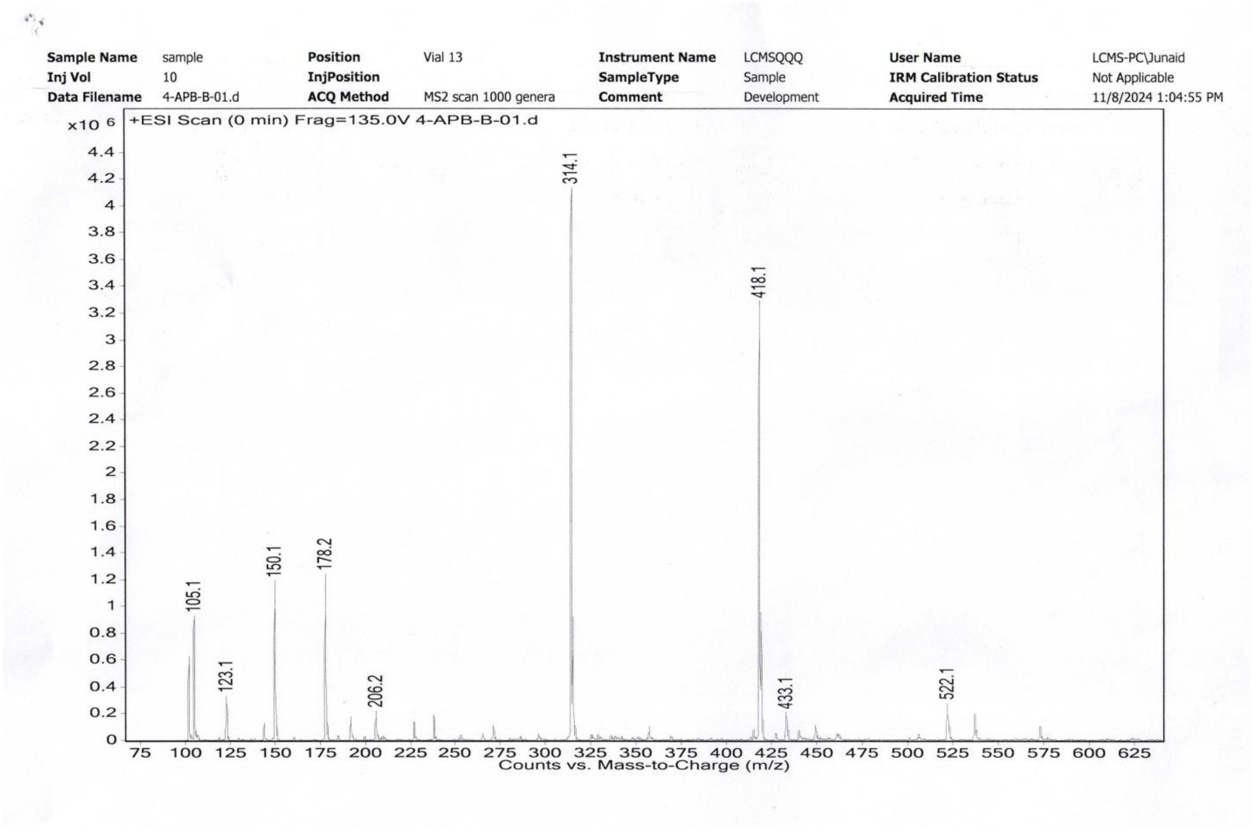

Figure S10. Mass Spec of 2-(4-aminophenyl)benzimidazole benzamide.
