# Supplementary material for: Benzamides of 2-(aminophenyl)benzimidazoles and 2-(aminophenyl)indoles as Anticancer Scaffolds: Synthesis, In Silico and In Vitro Evaluation
Source: Pharmaceuticals (Basel). 2026 Sep 17;19(9):1485. doi: 10.3390/ph19091485 (PMC13610037; doi:10.3390/ph19091485)
Supplement: Supplementary file 1 [file pharmaceuticals-19-01485-s001.zip › FTIR Specs.pdf]

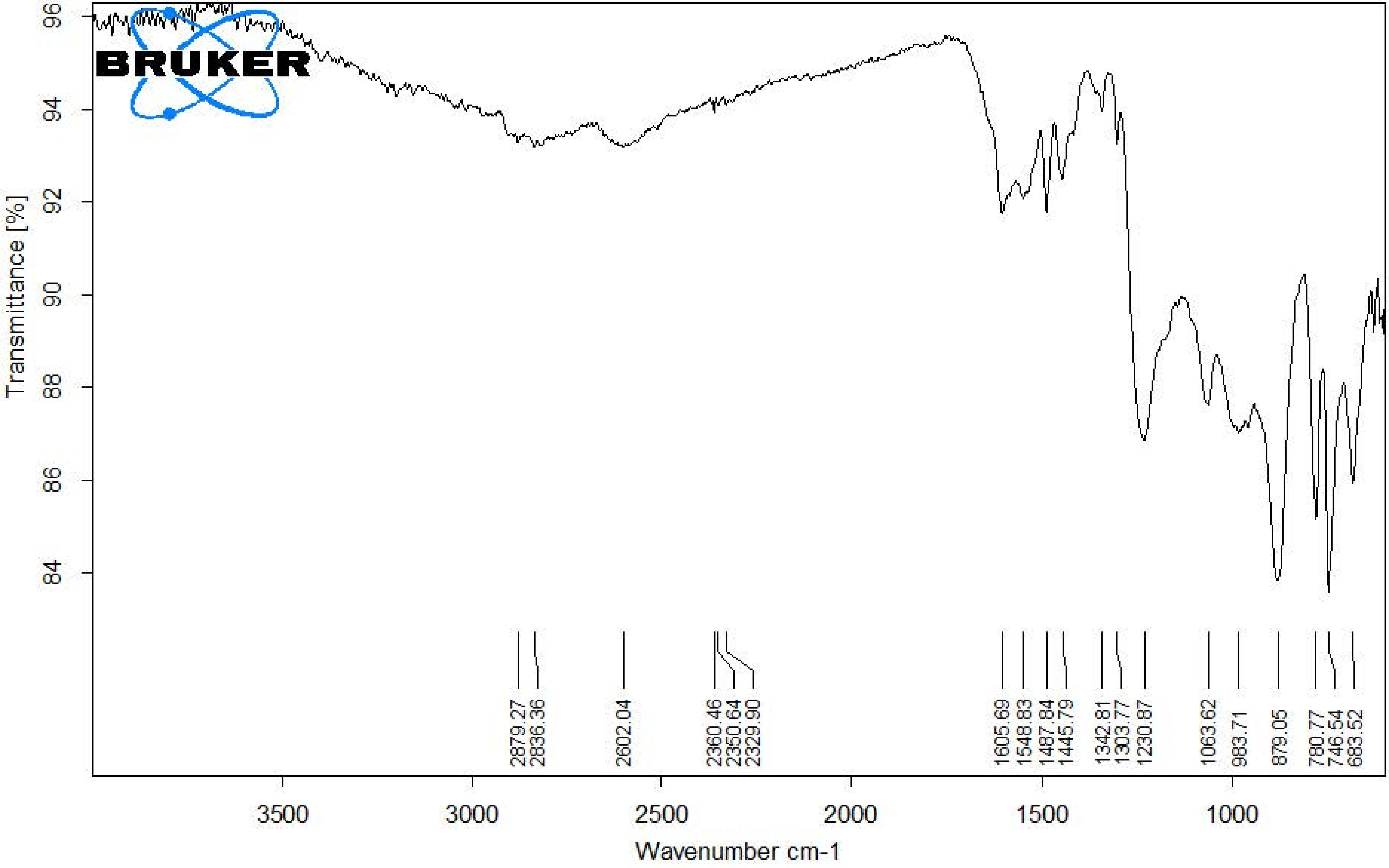

C:\Users\Riphah-Lab\Documents\Bruker\OPUS_7.5.18\DATA\MEAS\3-API-24.0	3-API-24	Instrument type and / or accessory	11/17/2024

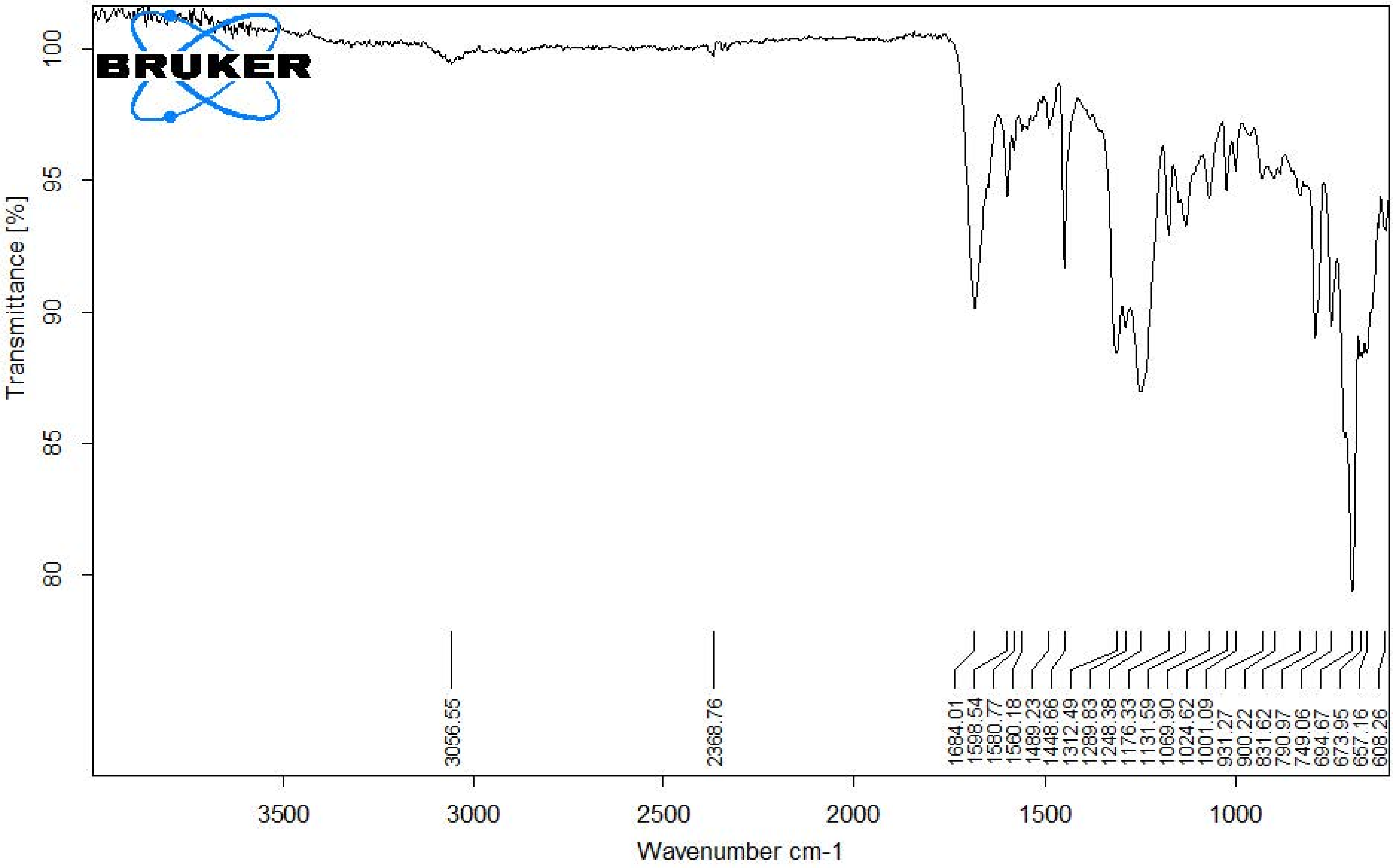

C:\Users\Riphah-Lab\Documents\Bruker\OPUS_7.5.18\DATA\MEAS\3-API-BEN-24.0	3-API-BEN-24	Instrument type and / or accessory	11/17/2024

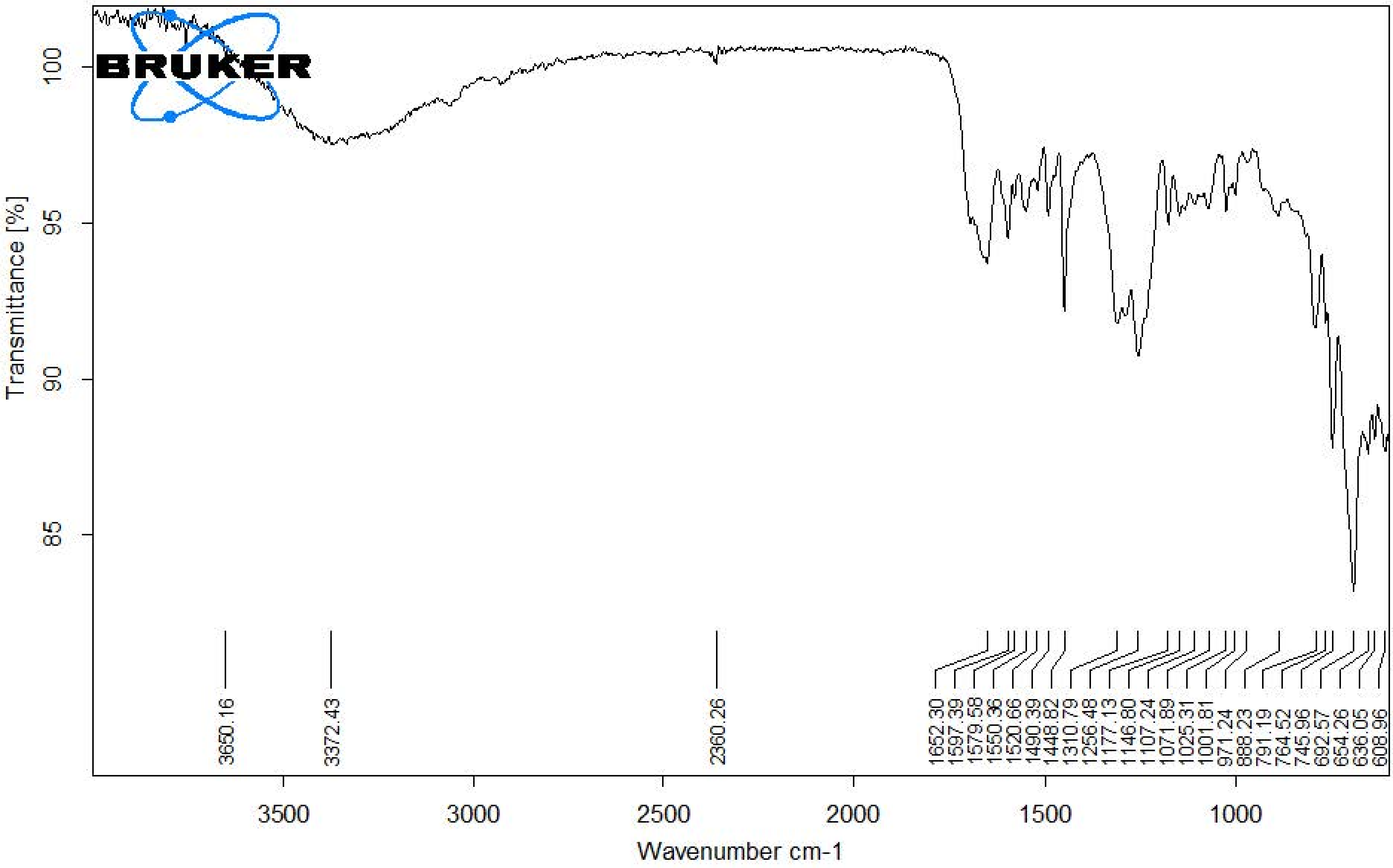

C:\Users\Riphah-Lab\Documents\Bruker\OPUS_7.5.18\DATA\MEAS\3-APB-BEN-24.0 3-APB-BEN-24	Instrument type and / or accesso	11/17/2024

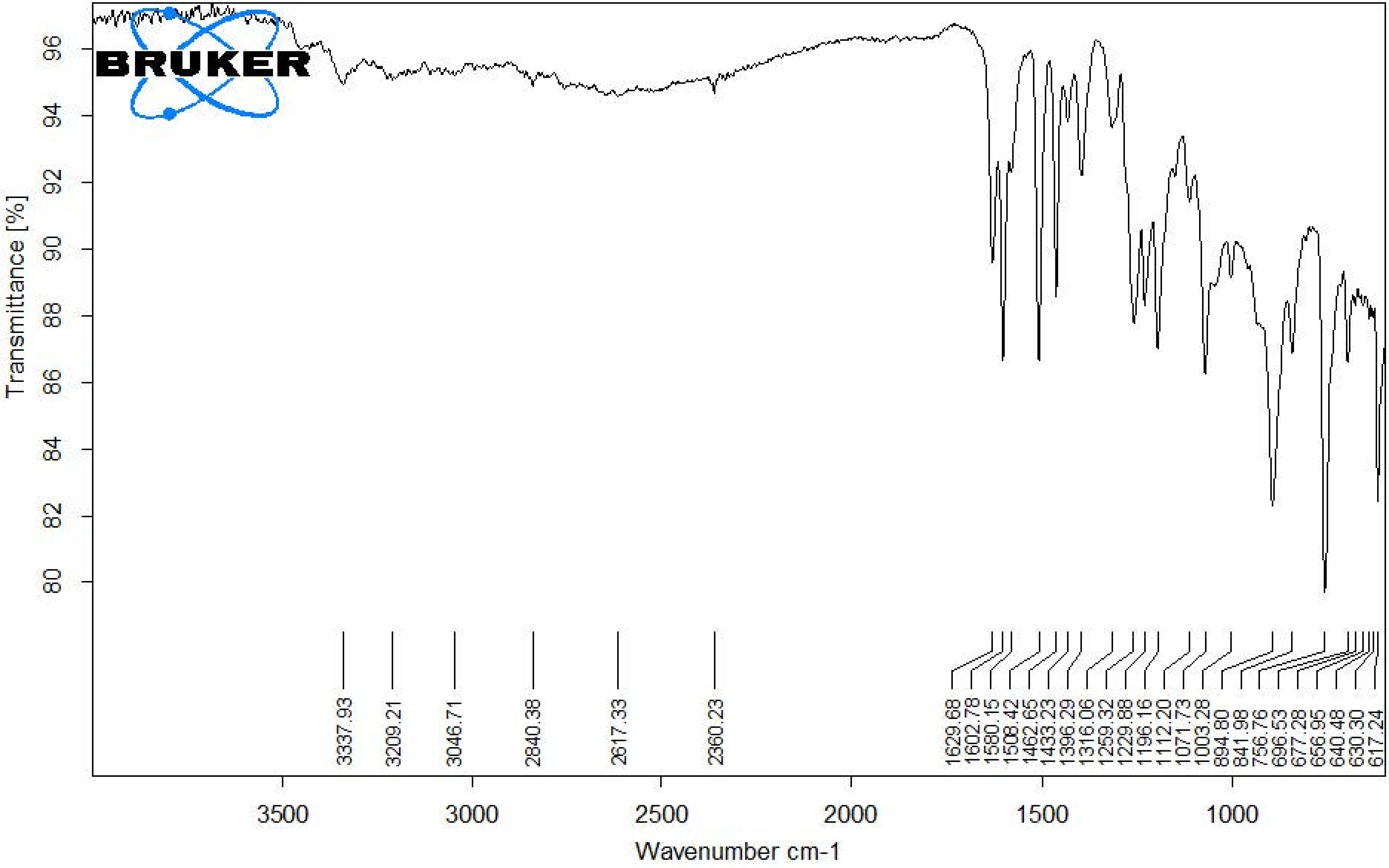

C:\Users\Riphah-Lab\Documents\Bruker\OPUS_7.5.18\DATA\MEAS\4-APB-24.0 4-APB-24 Instrument type and / or accessory	11/17/2024

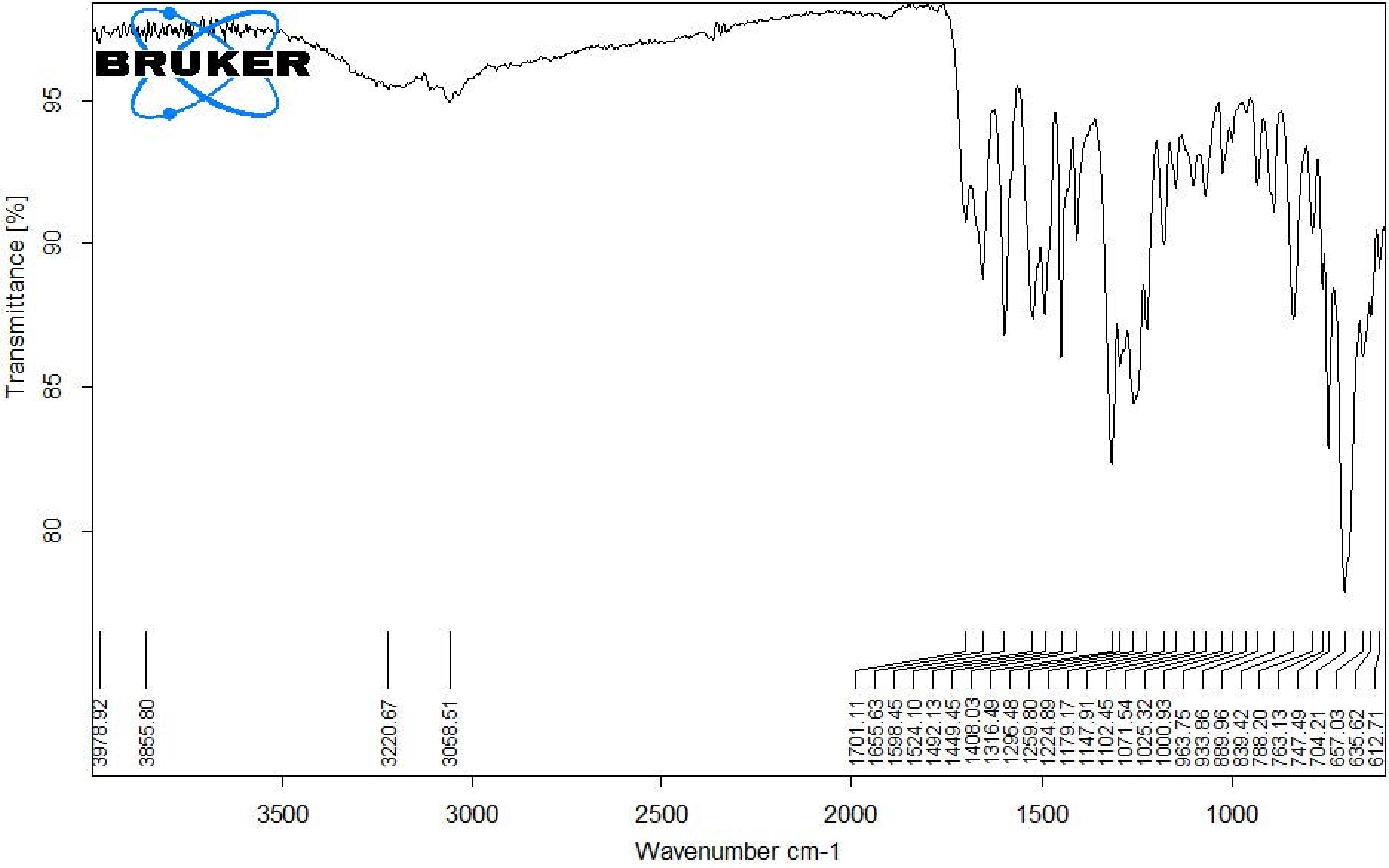

C:\Users\Riphah-Lab\Documents\Bruker\OPUS_7.5.18\DATA\MEAS\4-APB-BEN-24.0	4-APB-BEN-24	Instrument type and / or accesso	11/17/2024

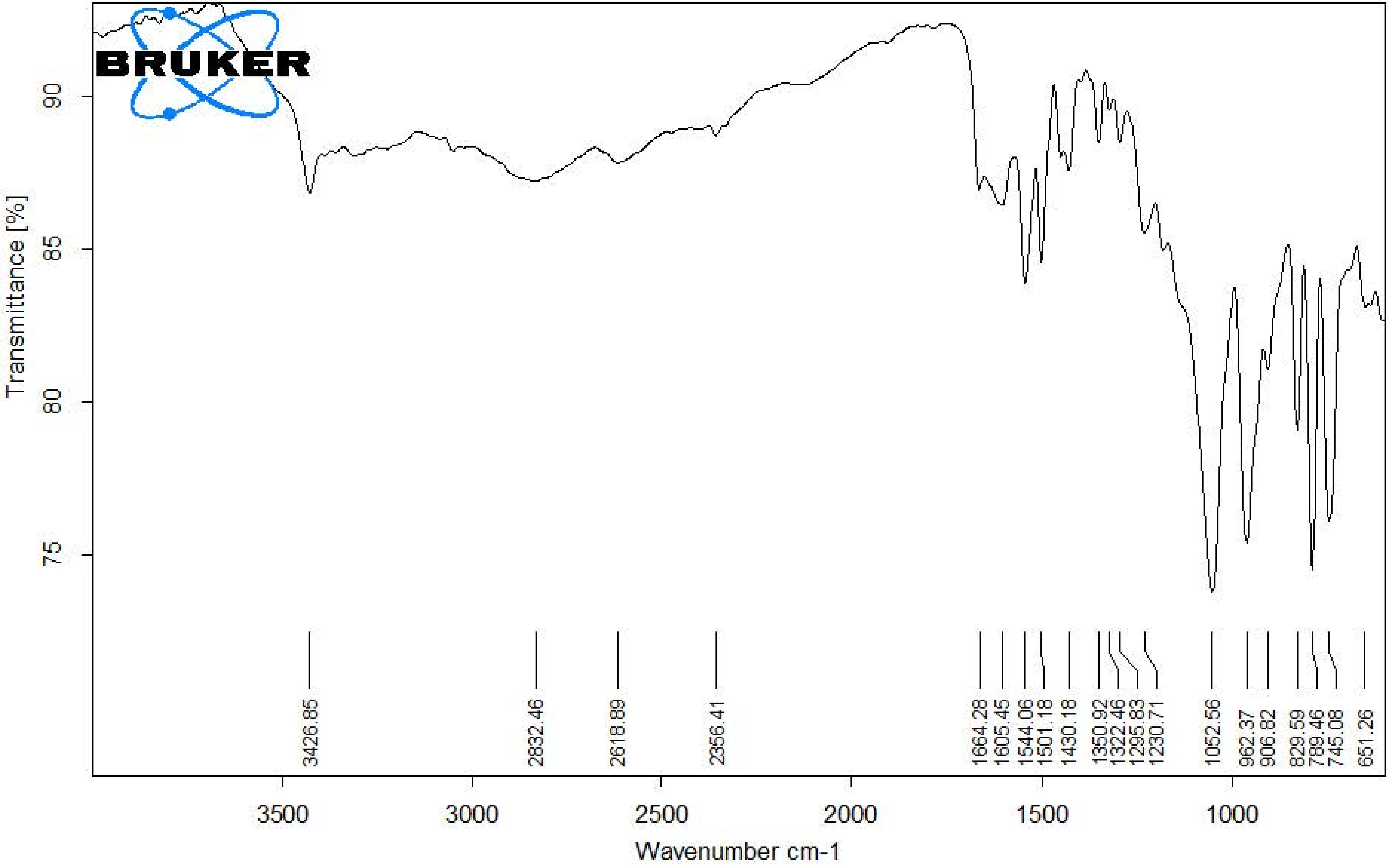

C:\Users\Riphah-Lab\Documents\Bruker\OPUS_7.5.18\DATA\MEAS\4-API-24.0	4-API-24	Instrument type and / or accessory	11/17/2024

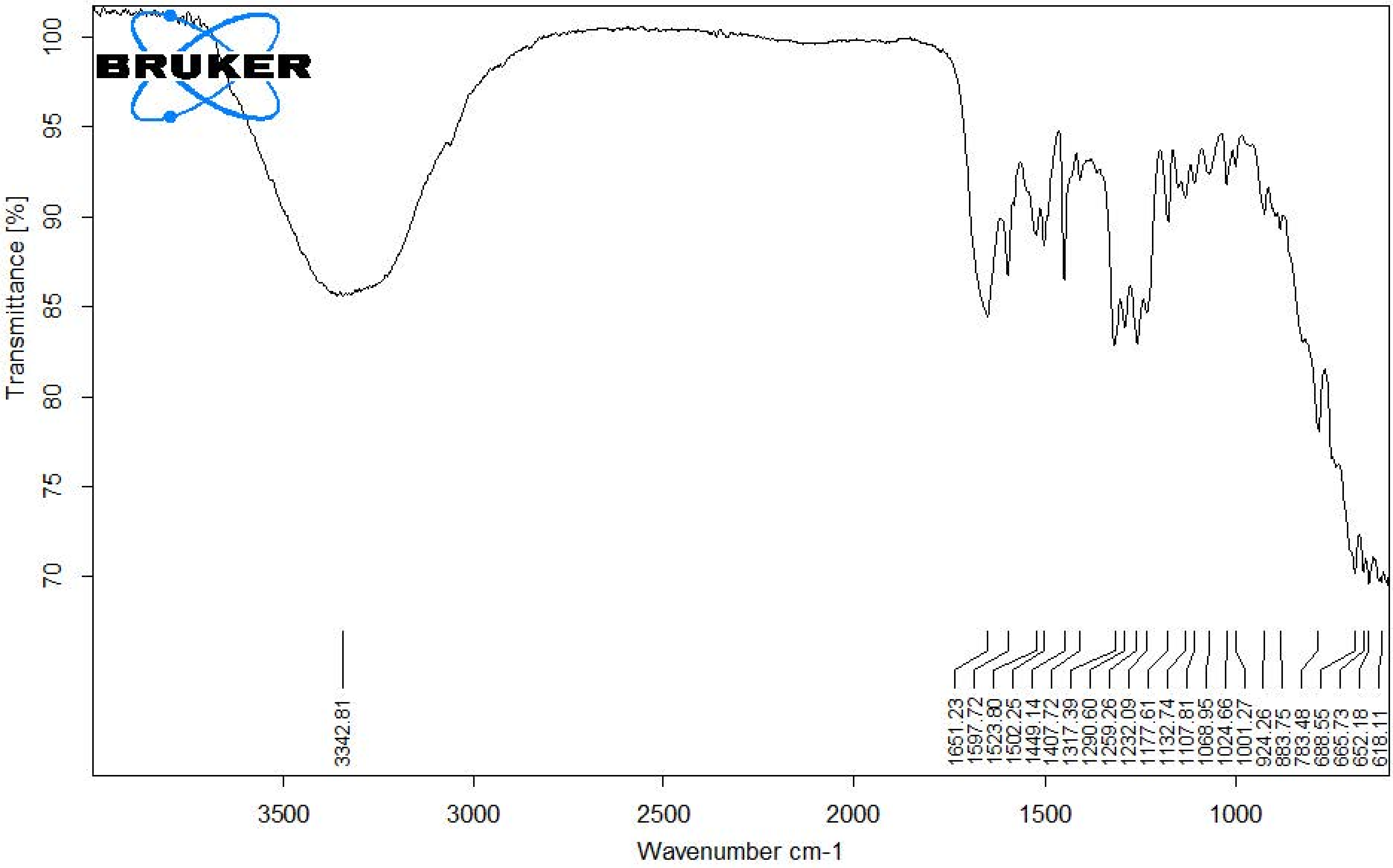

C:\Users\Riphah-Lab\Documents\Bruker\OPUS_7.5.18\DATA\MEAS\4-API-BEN-24.0	4-API-BEN-24	Instrument type and / or accessory	11/17/2024
