# Supplementary material for: Benzamides of 2-(aminophenyl)benzimidazoles and 2-(aminophenyl)indoles as Anticancer Scaffolds: Synthesis, In Silico and In Vitro Evaluation
Source: Pharmaceuticals (Basel). 2026 Sep 17;19(9):1485. doi: 10.3390/ph19091485 (PMC13610037; doi:10.3390/ph19091485)
Supplement: Supplementary file 1 [file pharmaceuticals-19-01485-s001.zip › HEJ assay Results 1st 4 compounds.pdf]

INTERNATIONAL CENTER FOR CHEMICAL AND BIOLOGICAL SCIENCES

H.E.J. RESEARCH INSTITUTE OF CHEMISTRY
AND
DR. PANJWANI CENTER FOR MOLECULAR MEDICINE AND DRUG RESEARCH
THIRD WORLD CENTER
UNIVERSITY OF KARACHI

RESULTS OF ANTI INFLAMMATORY/CYTOTOXICITY STUDIES

Date: 30-12-24

Submitted By: Adil Saeed **Lab. No/intercom No:** ....

Name of Supervisor: .... **Institution:** RIPAH

Name of Assay: MTT (Hela) **Amount Used (mg):** ....

Proposed Implications of the Anti inflammatory/Cytotoxicity Activity:

....

....

....

Sample Code	Conc. (~~µg/mL~~/µm)	% Inhibition/Stimulation	$IC_{50} \pm SD$ (100, 10, 1)
3 API-B	100	71.59	61.07 ± 0.27
3 APB-B	100	84:44	23.97 ± 0.17
4 API-B	100	31.41	-
4 APB-B	100	93.94	5.48 ± 0.01
STD. Doxorubicin	100	93.66	0.86 ± 0.12 µM

Remarks: 3 API-B showed low activity, 3 APB-B showed moderate activity, 4 API-B inhibit below fifty hence considered to be inactive while 4 APB-B is highly active against Hela cells.

[signature]
Evaluated By

[signature]
Section Incharge

Note: These studies are done on collaborative basis; both collaborators will share data for relevant publication.

INTERNATIONAL CENTER FOR CHEMICAL AND BIOLOGICAL SCIENCES

H.E.J. RESEARCH INSTITUTE OF CHEMISTRY
AND
DR. PANJWANI CENTER FOR MOLECULAR MEDICINE AND DRUG RESEARCH
THIRD WORLD CENTER
UNIVERSITY OF KARACHI

RESULTS OF ANTI INFLAMMATORY/CYTOTOXICITY STUDIES

Date: 3-01-2025

Submitted By: Adil Saeed **Lab. No/intercom No:**

Name of Supervisor: **Institution:** RIPAH Institute of Pharmaceutical Science Islamabad

Name of Assay: MTT Anticancer Activity (PC3 cell line) **Amount Used (mg):** 1 mg

Proposed Implications of the Anti inflammatory/Cytotoxicity Activity:

Sample Code	Conc. (µg/mL/µm)	% Inhibition/Stimulation	$IC_{50} \pm SD$
4 APB-B	100 µM	88.94	17.7 ± 0.22
4 API-B	100 µM	40.52	–
3 APB-B	100 µM	90.47	33.81 ± 0.33
3 API-B	100 µM	66.28	45.52 ± 1.2
STD Doxorubicin	10 µM	58.01	2.12 ± 0.3

Remarks: Sample 4APB-B and 3APB-B are highly active on PC3 cell line, Sample 3API-B showed moderate activity and Sample 4API.B showed ~~very~~ low activity.

Evaluated By — Section Incharge

Note: These studies are done on collaborative basis; both collaborators will share data for relevant publication.

INTERNATIONAL CENTER FOR CHEMICAL AND BIOLOGICAL SCIENCES

H.E.J. RESEARCH INSTITUTE OF CHEMISTRY
AND
DR. PANJWANI CENTER FOR MOLECULAR MEDICINE AND DRUG RESEARCH
THIRD WORLD CENTER
UNIVERSITY OF KARACHI

RESULTS OF ANTI INFLAMMATORY/CYTOTOXICITY STUDIES

Date: 2-2-25

Submitted By: Adil Saeed **Lab. No/intercom No:**

Name of Supervisor: **Institution:** RIPAH

Name of Assay: MTT (3T3) **Amount Used (mg):**

Proposed Implications of the Anti inflammatory/Cytotoxicity Activity:

Sample Code	Conc. (~~µg/mL~~/µm)	% Inhibition/Stimulation	$IC_{50} \pm SD$
3 API B	100	67.86	65.21 ± 0.06
3 APB B	100	11.71	–
4 API B	100	71.51	71.91 ± 0.01
4 APB B	100	80.39	58.54 ± 0.03
STD. Doxorubicin	30	95.8	0.05 ± 0.04

Remarks: from above pure samples, three showed low activity while one is inactive against 3T3 normal cells.

Evaluated By **Section Incharge**

Note: These studies are done on collaborative basis; both collaborators will share data for relevant publication.

Done

Sample Code No.: 3APB:B

INTERNATIONAL CENTER FOR CHEMICAL AND BIOLOGICAL SCIENCES

H.E.J. RESEARCH INSTITUTE OF CHEMISTRY
AND
DR. PANJWANI CENTER FOR MOLECULAR MEDICINE AND DRUG RESEARCH
UNIVERSITY OF KARACHI

Microplate Alamar Blue Assay (MABA)

(Preliminary Screening)

Date: 01/02/25

Submitted By: Adil Saeed **Lab. No:** .... **Tel. No.:** ....

Name of Supervisor: .... **Institution:** Riphah Institute Of Pharmaceutical Sciences

Name of Bacteria	Percent (%) Inhibition of Compound	Percent (%) Inhibition of Drug
Escherichia coli ATCC 25922	no inhibition	95.27
Bacillus subtilis ATCC 23857	no inhibition	96.83
Staphylococcus aureus NCTC ~~6571~~ 3277	no inhibition	94.40
Pseudomonas aeruginosa ATCC 10145	no inhibition	95.07
Salmonella typhi ATCC 14028	no inhibition	94.51

Keys: Amount of Compound .... **Amount of Drug** ....

Concentration of Compound 200 ug/ml **Concentration of Drug** 60 ug/ml

Remarks: Sample showed no inhibition

[signature]

Syeda Ramsha Zaki

Evaluated By

[signature]

Section Incharge

Sample Code No.: 4-API-B

INTERNATIONAL CENTER FOR CHEMICAL AND BIOLOGICAL SCIENCES

H.E.J. RESEARCH INSTITUTE OF CHEMISTRY
AND
DR. PANJWANI CENTER FOR MOLECULAR MEDICINE AND DRUG RESEARCH
UNIVERSITY OF KARACHI

Microplate Alamar Blue Assay (MABA)

(Preliminary Screening)

Date: 10-1-25

Submitted By: Adil Saeed **Lab. No:** .... **Tel. No.:** ....

Name of Supervisor: .... **Institution:** Riphah International Institute of Pharmaceutical Sciences

Name of Bacteria	Percent (%) Inhibition of Compound	Percent (%) Inhibition of Drug ofloxacin
Escherichia coli ATCC 25922	no inhibition	90.77
Bacillus subtilis ATCC 23857	no inhibition	91.52
Staphylococcus aureus NCTC ~~6571~~ 13277	no inhibition	83.82
Pseudomonas aeruginosa ATCC 10145	no inhibition	90.57
Salmonella typhi ATCC 14028	no inhibition	91.81

Keys: Amount of Compound........ Amount of Drug........

Concentration of Compound 200 ug/ml Concentration of Drug 50 ug/ml

Remarks: Sample showed no inhibition

[signature]

Syeda Ramsha Zaki

Evaluated By

Rukesh Maharjan

Section Incharge

Sample Code No.: 4-APB-B

INTERNATIONAL CENTER FOR CHEMICAL AND BIOLOGICAL SCIENCES

H.E.J. RESEARCH INSTITUTE OF CHEMISTRY
AND
DR. PANJWANI CENTER FOR MOLECULAR MEDICINE AND DRUG RESEARCH
UNIVERSITY OF KARACHI

Microplate Alamar Blue Assay (MABA)

(Preliminary Screening)

Date: 10-1-25

Submitted By: Adil Saeed **Lab. No:** .... **Tel. No.:** ....

Name of Supervisor: .... **Institution:** Riphah Institute of Pharmaceutical Sciences

Name of Bacteria	Percent (%) Inhibition of Compound	Percent (%) Inhibition of Drug
Escherichia coli ATCC 25922	no inhibition	90.77
Bacillus subtilis ATCC 23857	no inhibition	91.52
Staphylococcus aureus NCTC ~~6571~~ 13277	no inhibition	83.82
Pseudomonas aeruginosa ATCC 10145	no inhibition	90.57
Salmonella typhi ATCC 14028	no inhibition	91.81

Keys: Amount of Compound .... **Amount of Drug** ....

Concentration of Compound 200 ug/ml **Concentration of Drug** 50 ug/ml

Remarks: Sample showed no inhibition

Syeda Ramsha Zaki

Evaluated By

Section Incharge

Sample Code No.: [illegible] 3-API-B

INTERNATIONAL CENTER FOR CHEMICAL AND BIOLOGICAL SCIENCES

H.E.J. RESEARCH INSTITUTE OF CHEMISTRY
AND
DR. PANJWANI CENTER FOR MOLECULAR MEDICINE AND DRUG RESEARCH
UNIVERSITY OF KARACHI

Microplate Alamar Blue Assay (MABA)

(Preliminary Screening)

Date: 10-01-25

Submitted By: Adil Saeed Lab. No: .... Tel. No.: ....

Name of Supervisor: .... Institution: Riphah Institute of Pharmaceutical Sciences

Name of Bacteria	Percent (%) Inhibition of Compound	Percent (%) Inhibition of Drug Ofloxacin
Escherichia coli ATCC 25922	no inhibition	90.72
Bacillus subtilis ATCC 23857	no inhibition	91.52
Staphylococcus aureus NCTC ~~6571~~ 13277	no inhibition	83.82
Pseudomonas aeruginosa ATCC 10145	no inhibition	90.57
Salmonella typhi ATCC 14028	no inhibition	91.81

Keys: Amount of Compound........ Amount of Drug........

Concentration of Compound 200 ug/ml Concentration of Drug 50ug/ml

Remarks:

Sample showed no inhibition

Syeda Ramsha Zaki

Evaluated By

Rukesh Mahajan

Section Incharge
