# Supplementary material for: Benzamides of 2-(aminophenyl)benzimidazoles and 2-(aminophenyl)indoles as Anticancer Scaffolds: Synthesis, In Silico and In Vitro Evaluation
Source: Pharmaceuticals (Basel). 2026 Sep 17;19(9):1485. doi: 10.3390/ph19091485 (PMC13610037; doi:10.3390/ph19091485)

**Sample Name**	Blank	**Position**	Vial 1	**Instrument Name**	LCMSQQQ	**User Name**	LCMS-PC\Junaid
**Inj Vol**	10	**InjPosition**		**SampleType**	Sample	**IRM Calibration Status**	Not Applicable
**Data Filename**	Blank 16.d	**ACQ Method**	MS2 scan 1000 genera	**Comment**	Development	**Acquired Time**	11/8/2024 12:24:31 PM

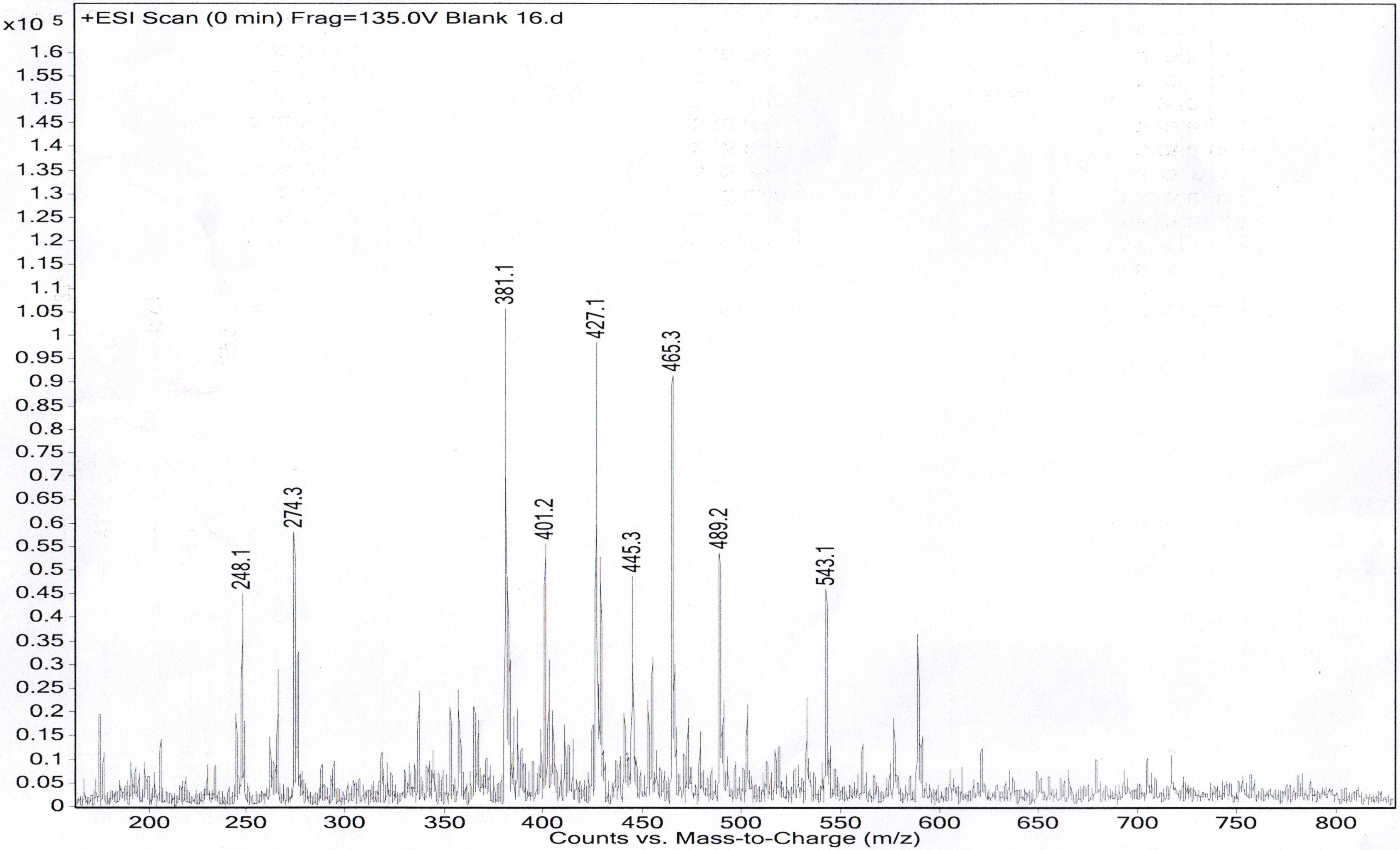

**Sample Name**	Blank	**Position**	Vial 1	**Instrument Name**	LCMSQQQ	**User Name**	LCMS-PC\Junaid
**Inj Vol**	10	**InjPosition**		**SampleType**	Sample	**IRM Calibration Status**	Not Applicable
**Data Filename**	Blank 02.d	**ACQ Method**	MS2 scan 1000 genera	**Comment**	Development	**Acquired Time**	11/8/2024 10:59:57 AM

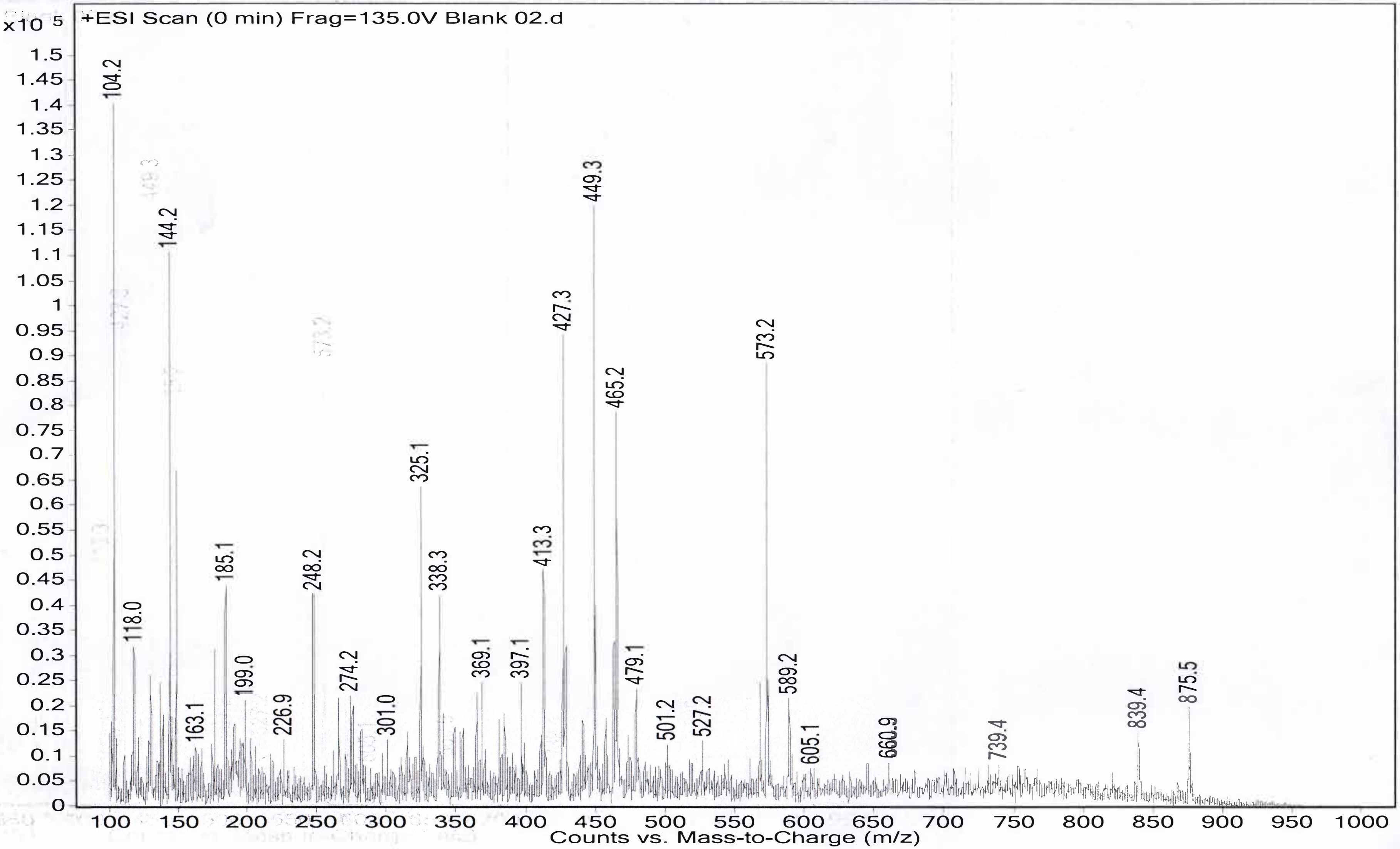

**Sample Name**	sample	**Position**	Vial 14	**Instrument Name**	LCMSQQQ	**User Name**	LCMS-PC\Junaid
**Inj Vol**	10	**InjPosition**		**SampleType**	Sample	**IRM Calibration Status**	Not Applicable
**Data Filename**	4-API-B-01.d	**ACQ Method**	MS2 scan 1000 genera	**Comment**	Development	**Acquired Time**	11/8/2024 1:17:00 PM

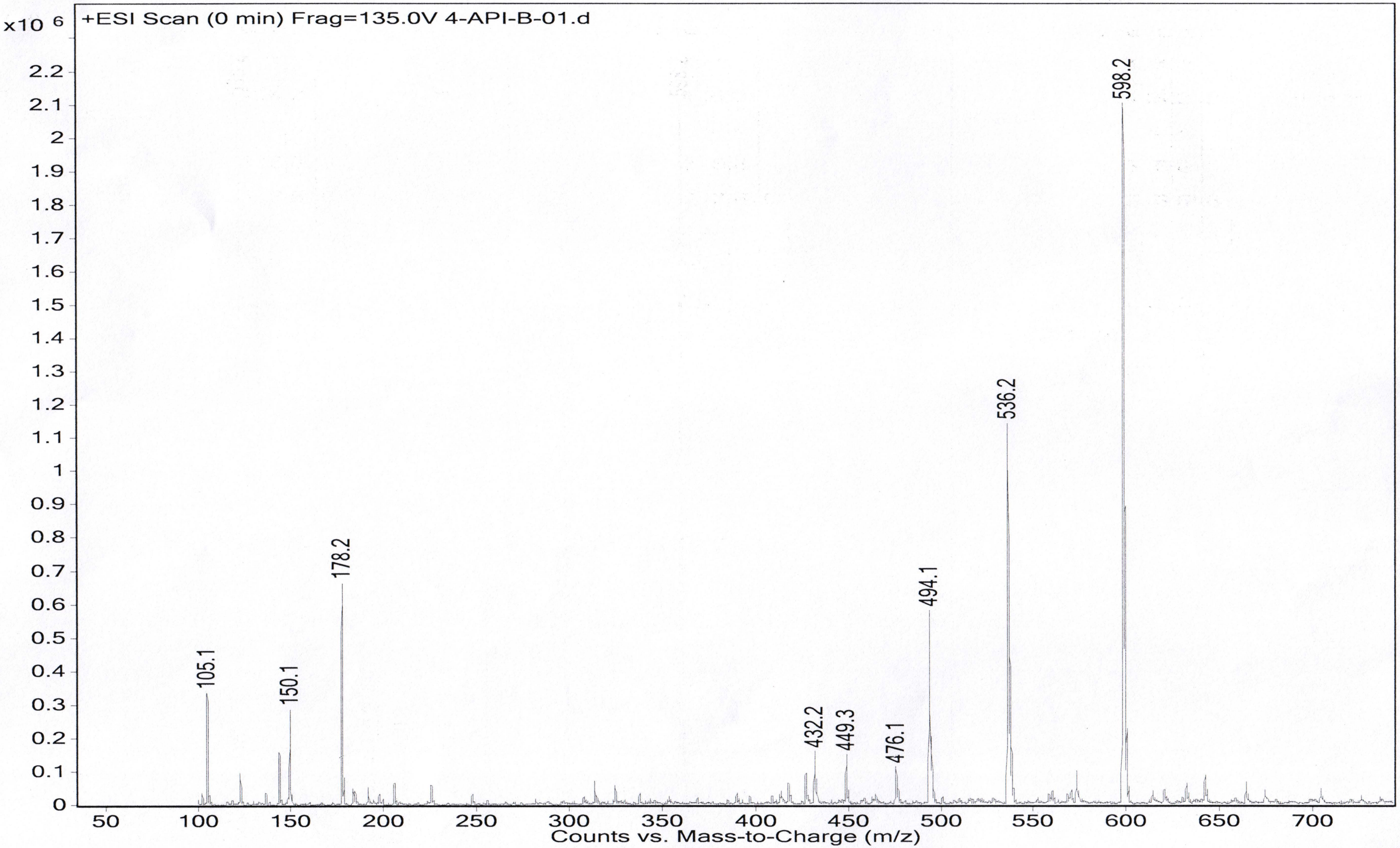

**Sample Name**	sample	**Position**	Vial 13	**Instrument Name**	LCMSQQQ	**User Name**	LCMS-PC\Junaid
**Inj Vol**	10	**InjPosition**		**SampleType**	Sample	**IRM Calibration Status**	Not Applicable
**Data Filename**	4-APB-B-01.d	**ACQ Method**	MS2 scan 1000 genera	**Comment**	Development	**Acquired Time**	11/8/2024 1:04:55 PM

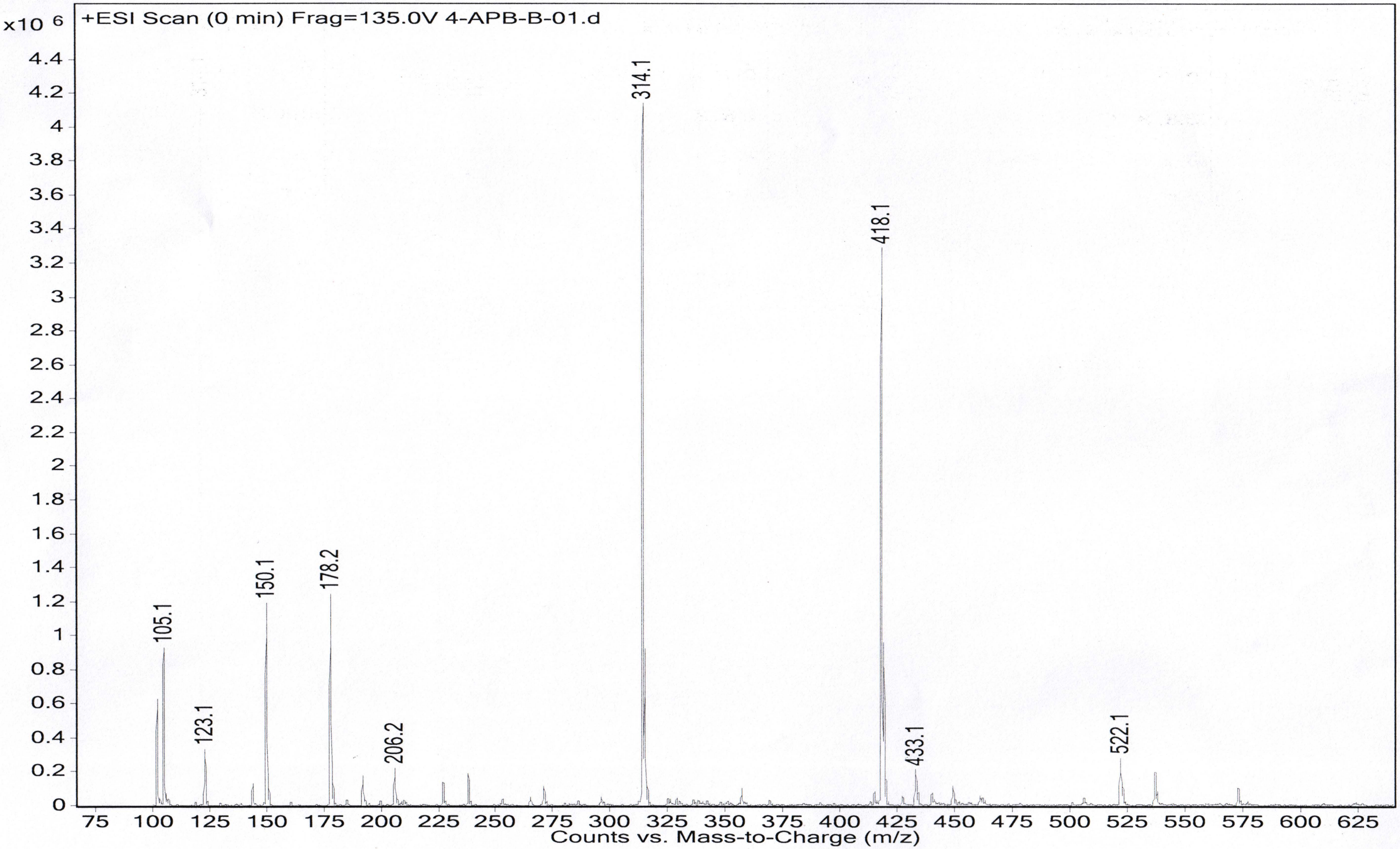

**Sample Name**	sample	**Position**	Vial 12	**Instrument Name**	LCMSQQQ	**User Name**	LCMS-PC\Junaid
**Inj Vol**	10	**InjPosition**		**SampleType**	Sample	**IRM Calibration Status**	Not Applicable
**Data Filename**	3-API-G-01.d	**ACQ Method**	MS2 scan 1000 genera	**Comment**	Development	**Acquired Time**	11/8/2024 12:52:51 PM

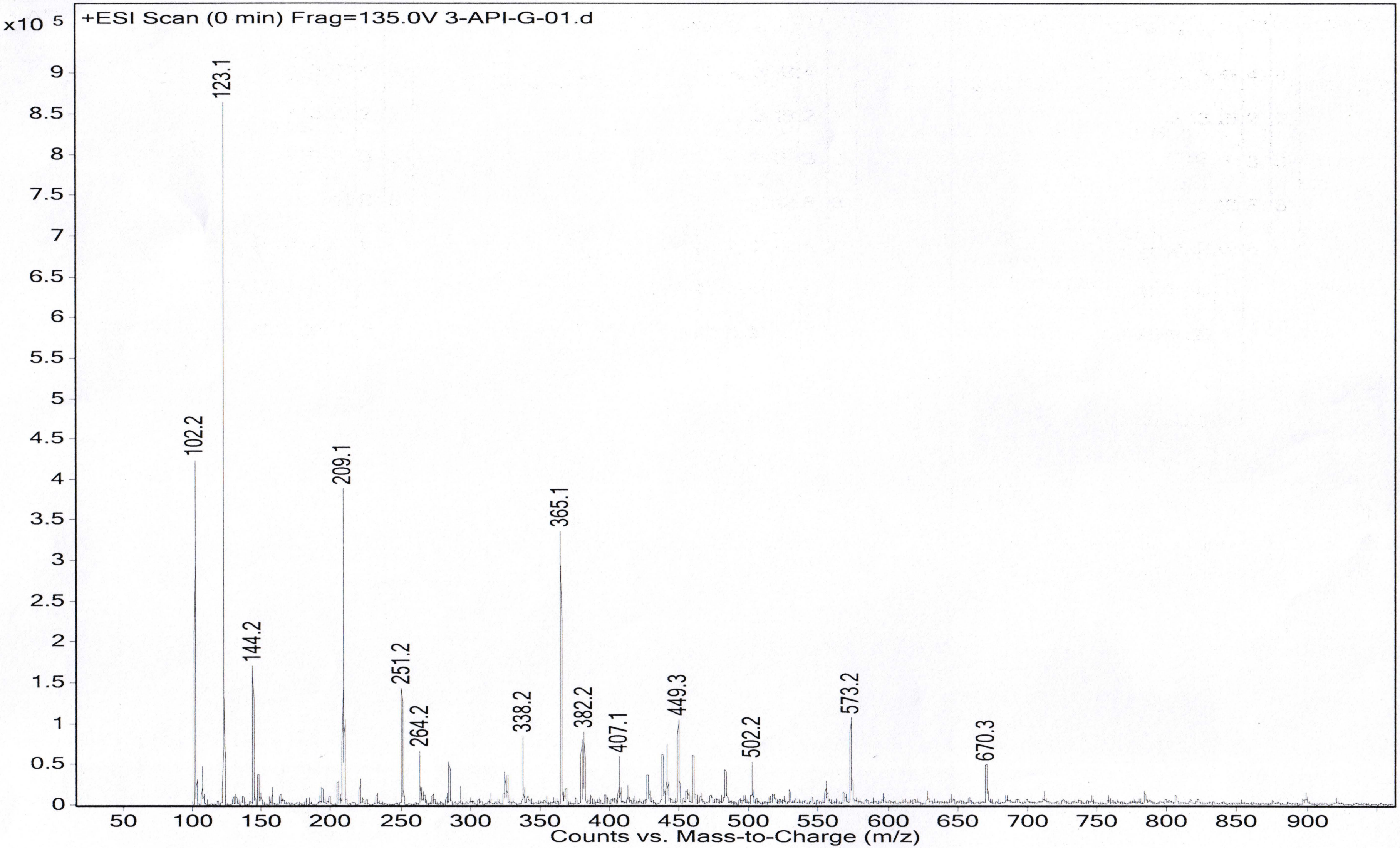

**Sample Name**	sample	**Position**	Vial 11	**Instrument Name**	LCMSQQQ	**User Name**	LCMS-PC\Junaid
**Inj Vol**	10	**InjPosition**		**SampleType**	Sample	**IRM Calibration Status**	Not Applicable
**Data Filename**	3-API-B-01.d	**ACQ Method**	MS2 scan 1000 genera	**Comment**	Development	**Acquired Time**	11/8/2024 12:40:44 PM

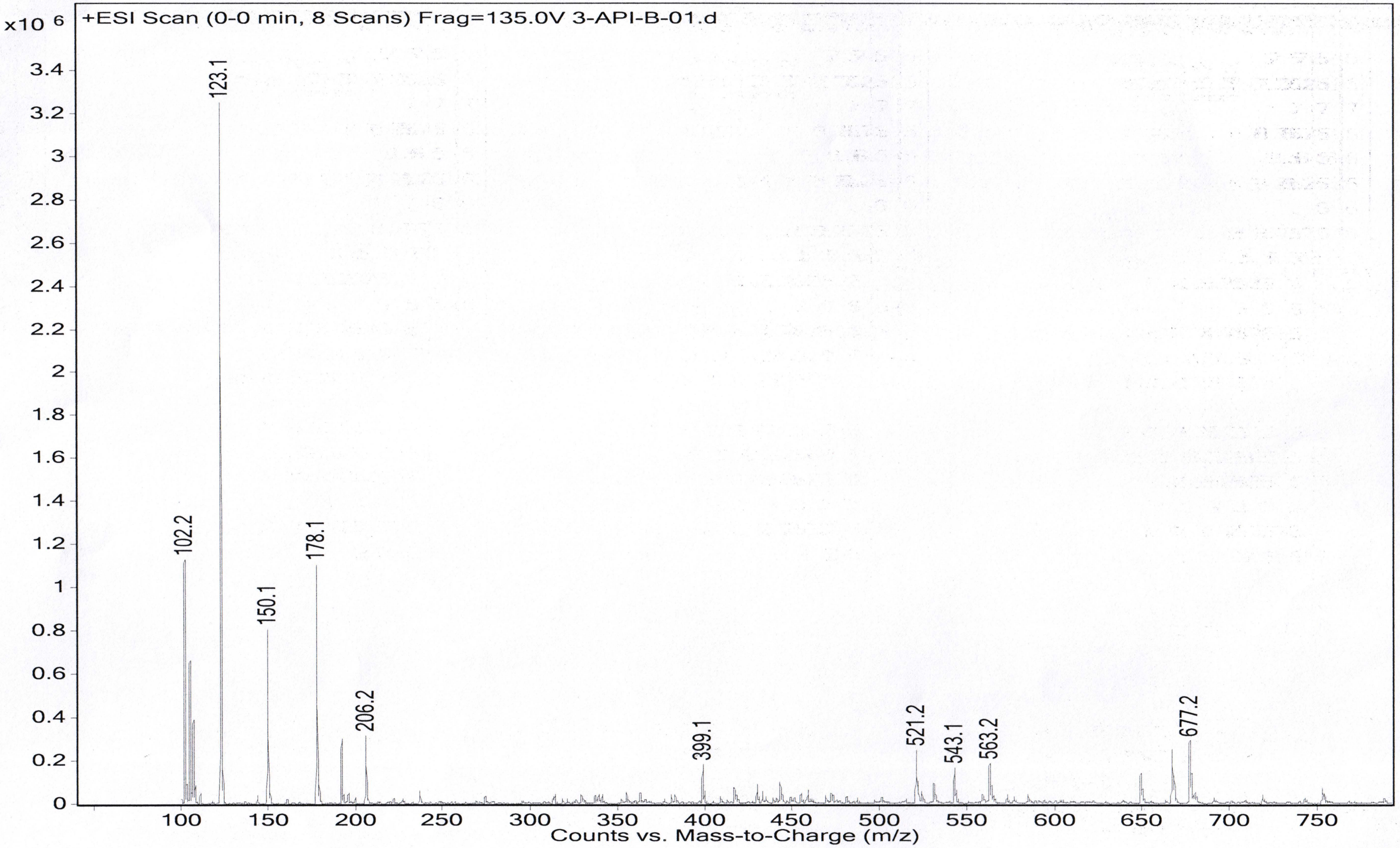

**Sample Name**	sample	**Position**	Vial 10	**Instrument Name**	LCMSQQQ	**User Name**	LCMS-PC\Junaid
**Inj Vol**	10	**InjPosition**		**SampleType**	Sample	**IRM Calibration Status**	Not Applicable
**Data Filename**	3-APB-B-01.d	**ACQ Method**	MS2 scan 1000 genera	**Comment**	Development	**Acquired Time**	11/8/2024 12:28:37 PM

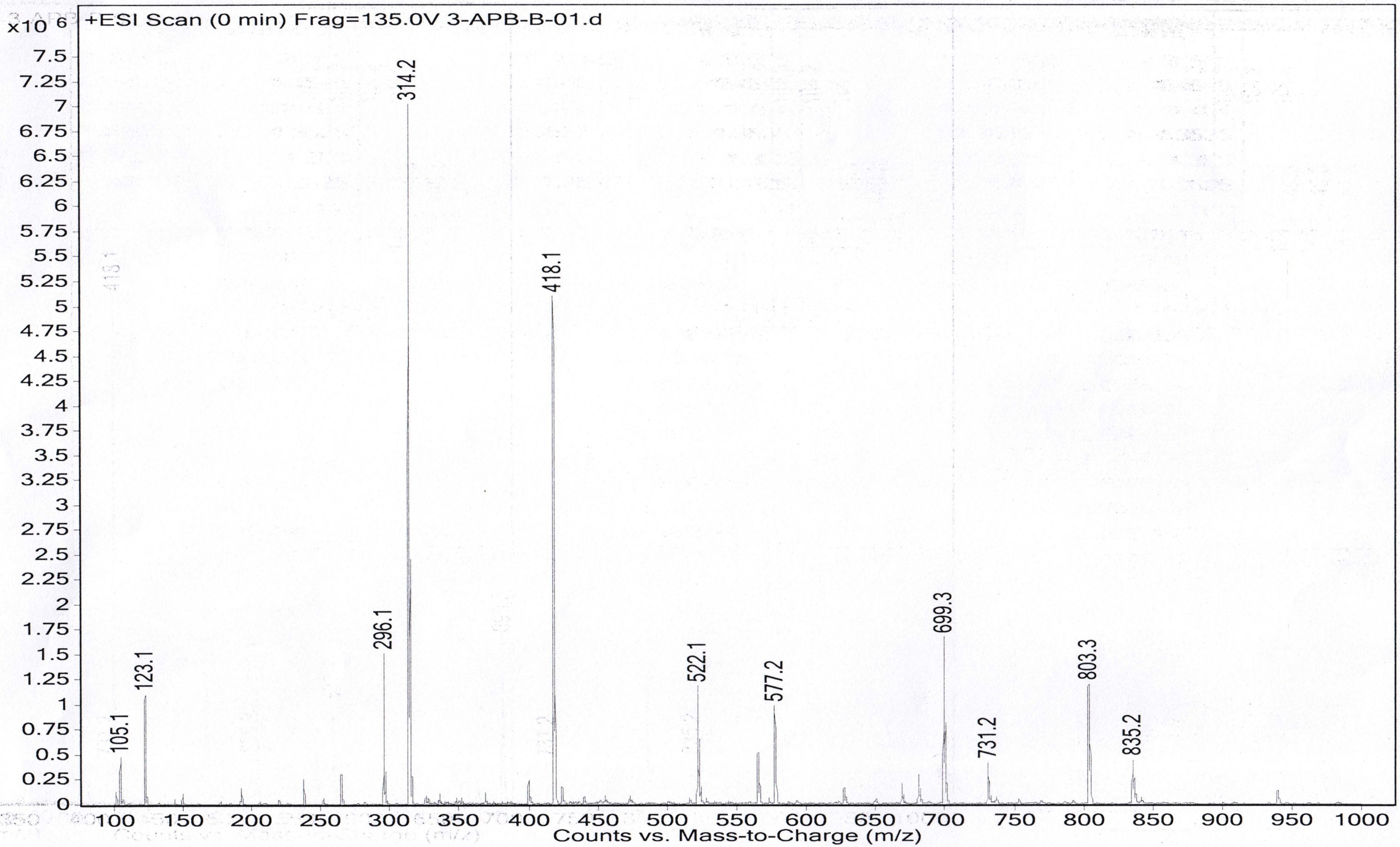

Supplement: Supplementary file 1 [file pharmaceuticals-19-01485-s001.zip › MASS SPECS 4 COMPOUNDS.pdf]
