# Supplementary material for: Benzamides of 2-(aminophenyl)benzimidazoles and 2-(aminophenyl)indoles as Anticancer Scaffolds: Synthesis, In Silico and In Vitro Evaluation
Source: Pharmaceuticals (Basel). 2026 Sep 17;19(9):1485. doi: 10.3390/ph19091485 (PMC13610037; doi:10.3390/ph19091485)

M4APIG_1HNMR_DMSO

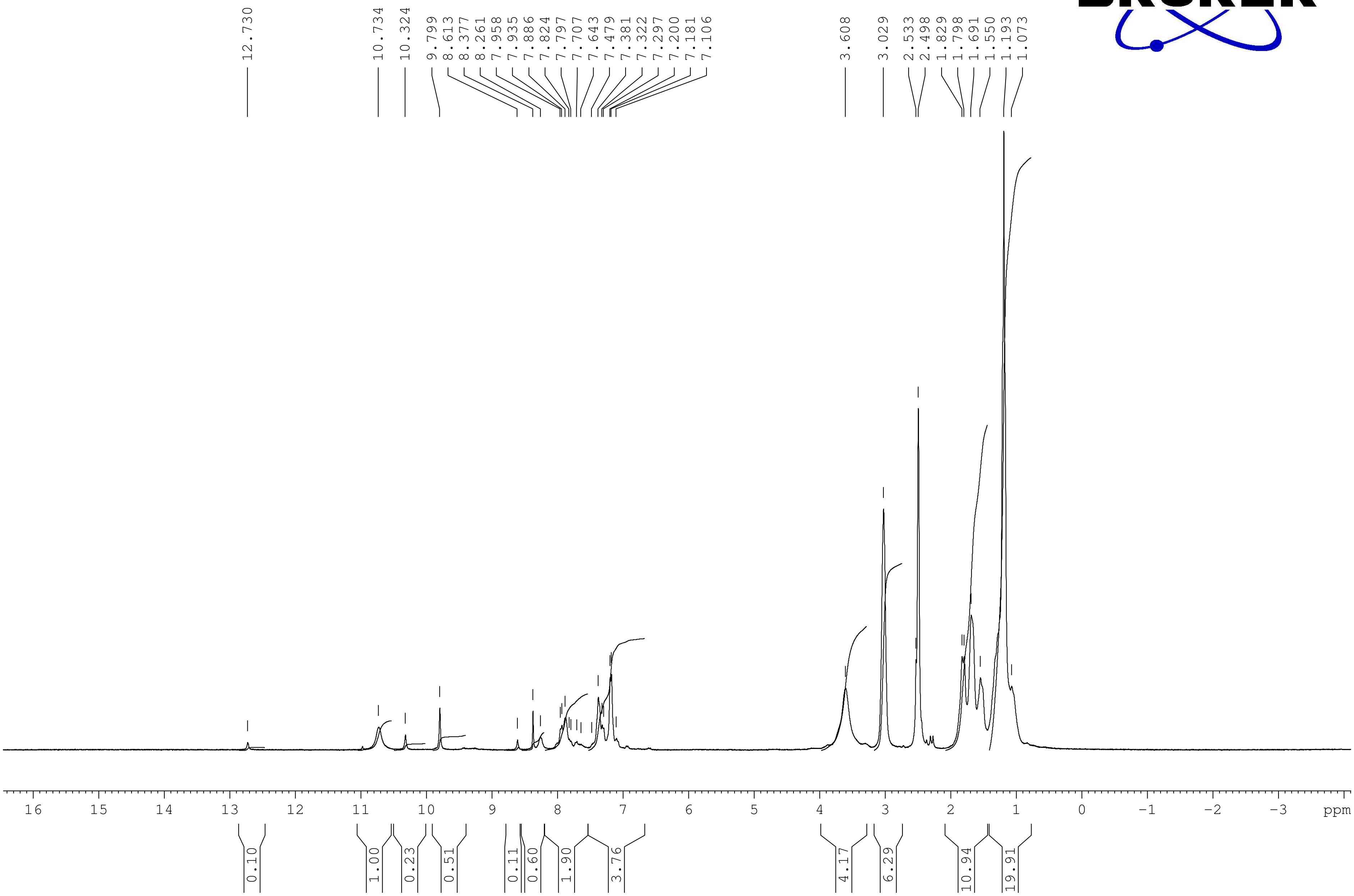

M4APIG_1HNMR_DMSO

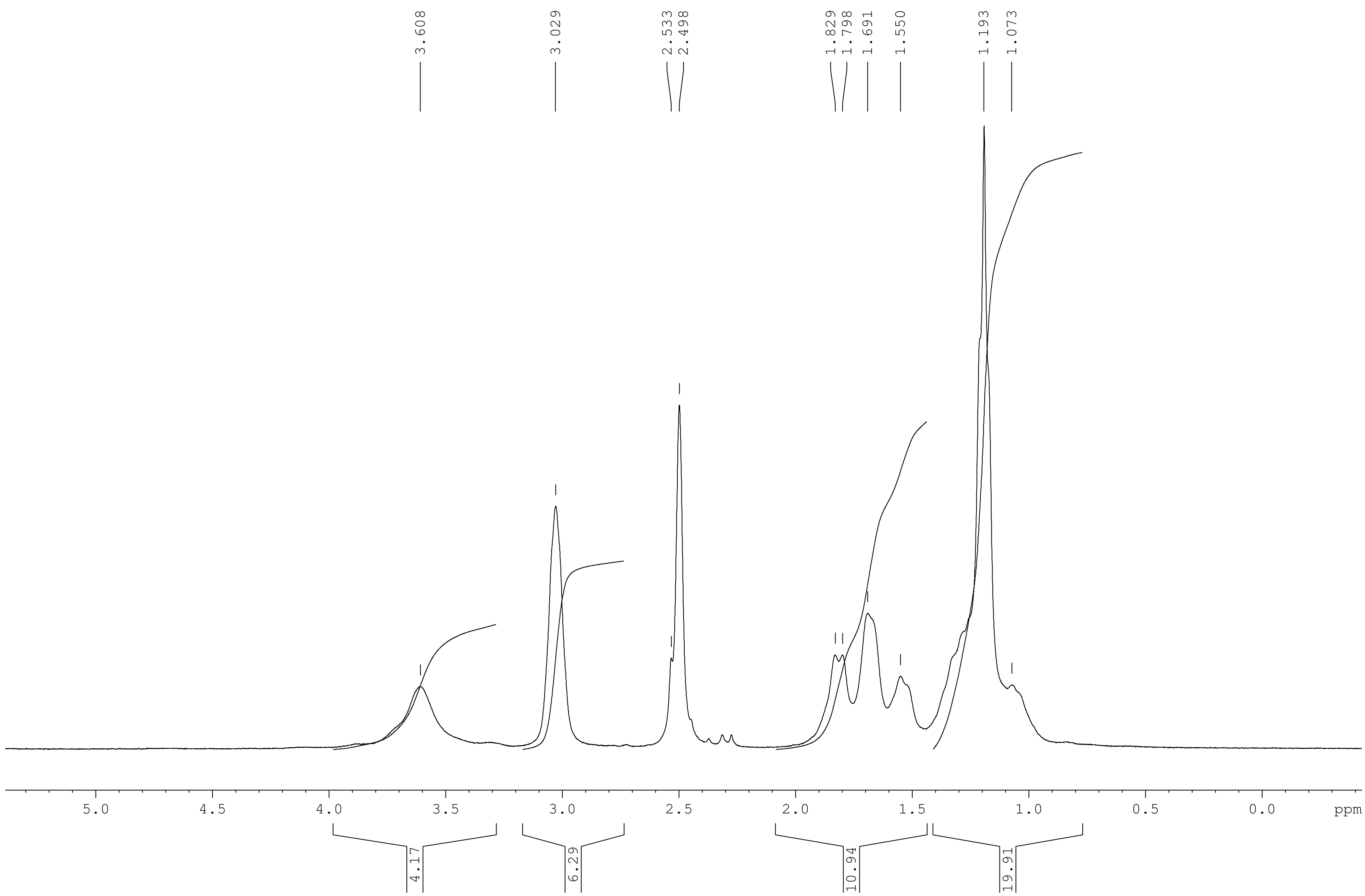

M4APIG_1HNMR_DMSO

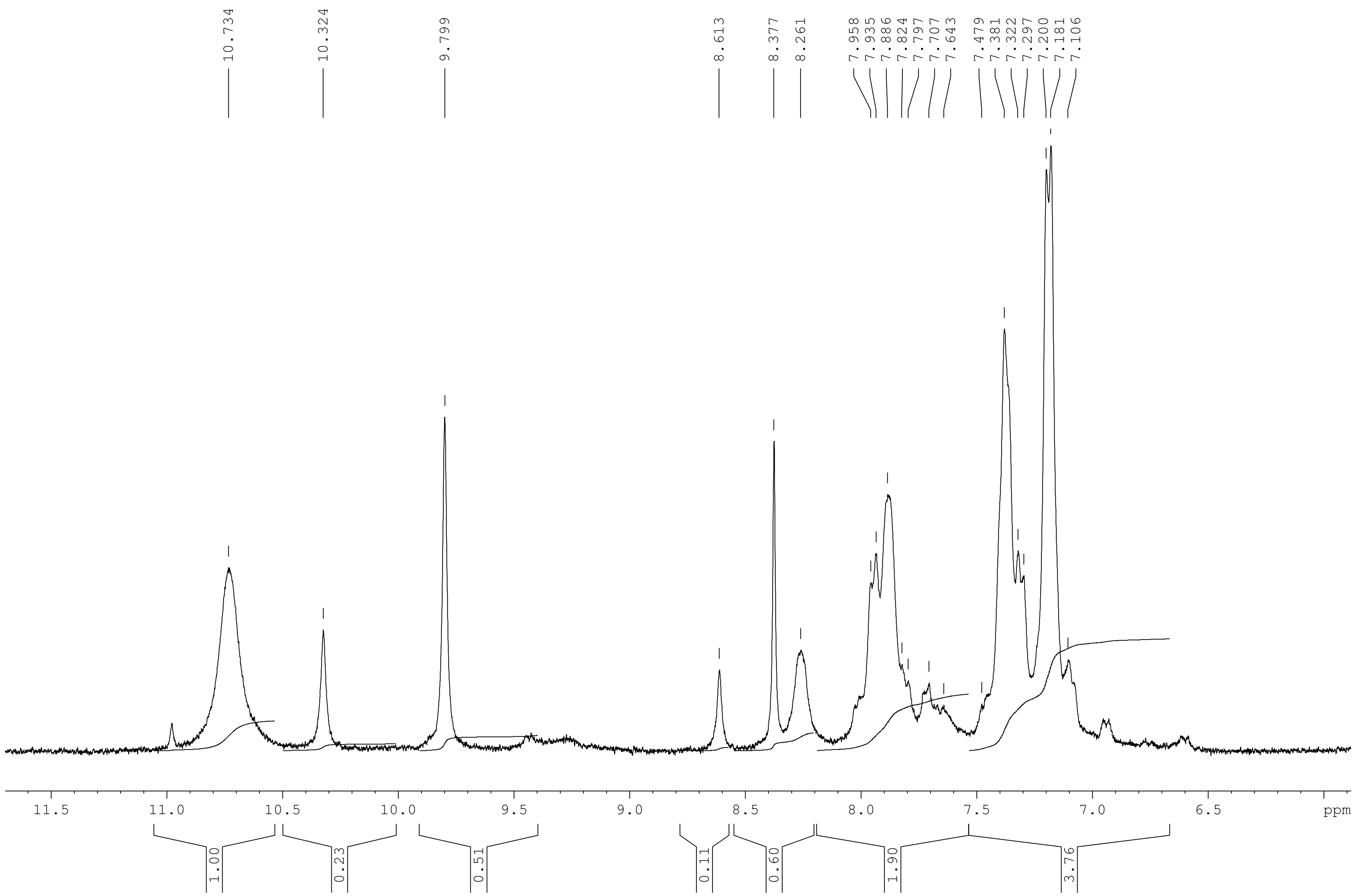

Supplement: Supplementary file 1 [file pharmaceuticals-19-01485-s001.zip › NMR of metal complex.pdf]
