# Supplementary material for: Benzamides of 2-(aminophenyl)benzimidazoles and 2-(aminophenyl)indoles as Anticancer Scaffolds: Synthesis, In Silico and In Vitro Evaluation
Source: Pharmaceuticals (Basel). 2026 Sep 17;19(9):1485. doi: 10.3390/ph19091485 (PMC13610037; doi:10.3390/ph19091485)
Supplement: Supplementary file 1 [file pharmaceuticals-19-01485-s001.zip › Raw NMR spectra.pdf]

3NPT-CNMR

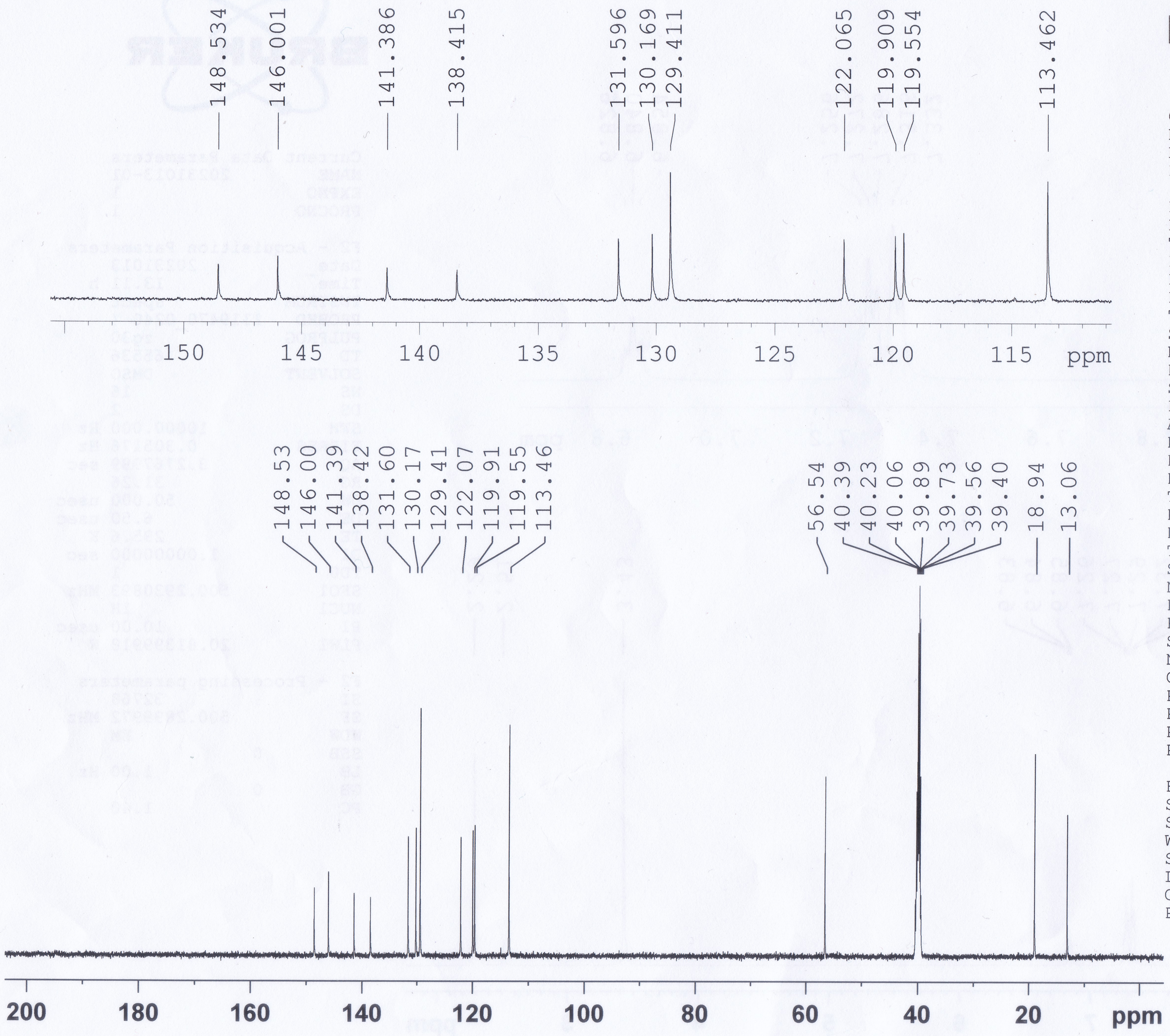

Current Data Parameters

NAME	20231013-02	
EXPNO	1	
PROCNO	1	

F2 - Acquisition Parameters

Date_	20231013	
Time	13.40	h
INSTRUM	spect	
PROBHD	Z119470_0240 (	
PULPROG	zgpg30	
TD	65536	
SOLVENT	DMSO	
NS	103	
DS	4	
SWH	29761.904	Hz
FIDRES	0.908261	Hz
AQ	1.1010048	sec
RG	189	
DW	16.800	usec
DE	6.50	usec
TE	296.4	K
D1	2.00000000	sec
D11	0.03000000	sec
TD0	1	
SFO1	125.8106004	MHz
NUC1	13C	
P1	10.00	usec
PLW1	88.97299957	W
SFO2	500.2920012	MHz
NUC2	1H	
CPDPRG[2	waltz16	
PCPD2	80.00	usec
PLW2	20.81399918	W
PLW12	0.31764621	W
PLW13	0.15920430	W

F2 - Processing parameters

SI	32768	
SF	125.7980206	MHz
WDW	EM	
SSB	0	
LB	1.00	Hz
GB	0	
PC	1.40	

3NPT-HNMR

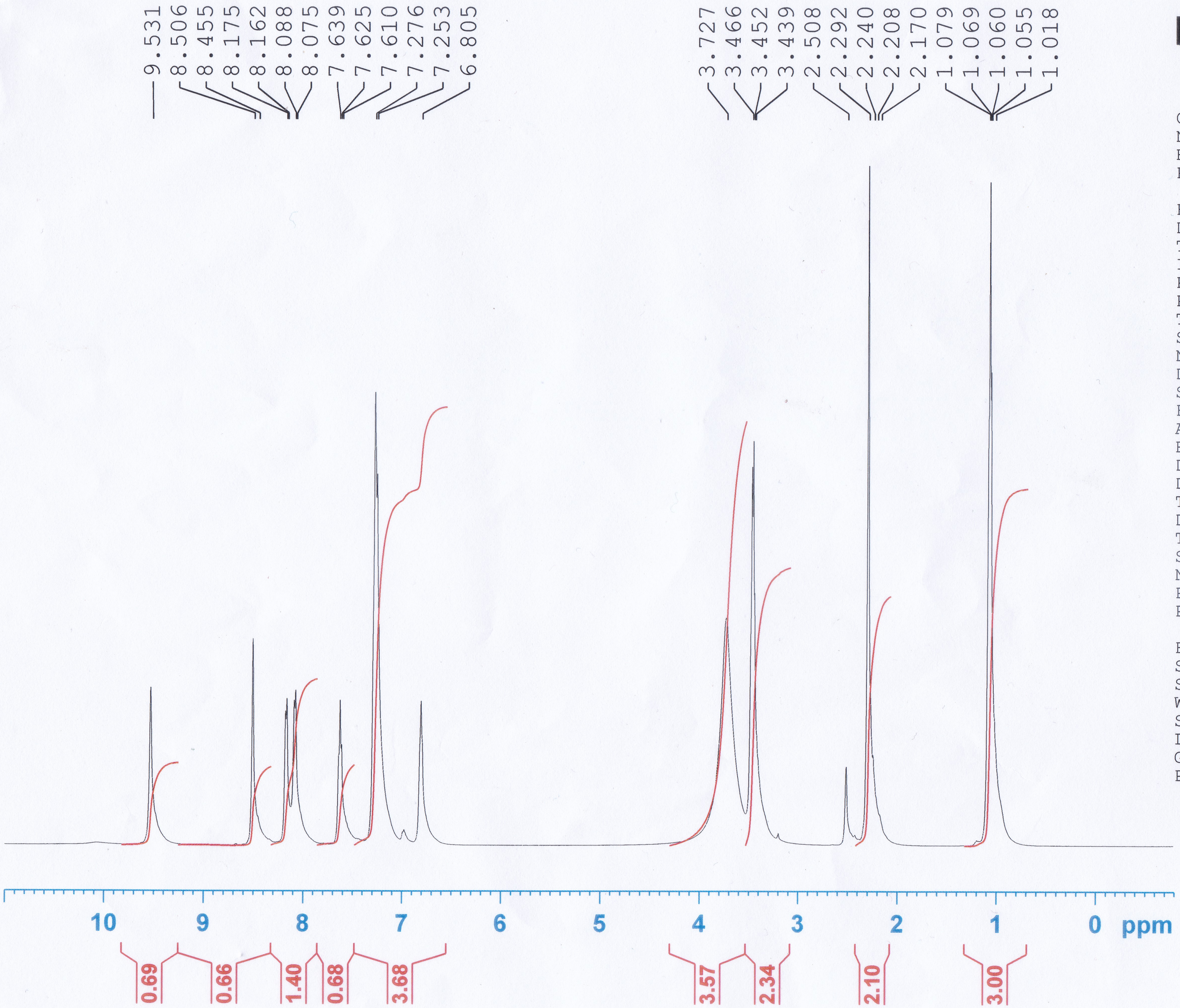

BRUKER

Current Data Parameters

NAME	20231013-02
EXPNO	2
PROCNO	1

F2 - Acquisition Parameters

Date_	20231013
Time	13.44 h
INSTRUM	spect
PROBHD	Z119470_0240 (
PULPROG	zg30
TD	65536
SOLVENT	DMSO
NS	16
DS	2
SWH	10000.000 Hz
FIDRES	0.305176 Hz
AQ	3.2767999 sec
RG	19.43
DW	50.000 usec
DE	6.50 usec
TE	296.2 K
D1	1.00000000 sec
TD0	1
SFO1	500.2930893 MHz
NUC1	1H
P1	10.00 usec
PLW1	20.81399918 W

F2 - Processing parameters

SI		65536
SF		500.2900000 MHz
WDW		EM
SSB	0	
LB		0.30 Hz
GB	0	
PC		1.00

4NPT-HNMR

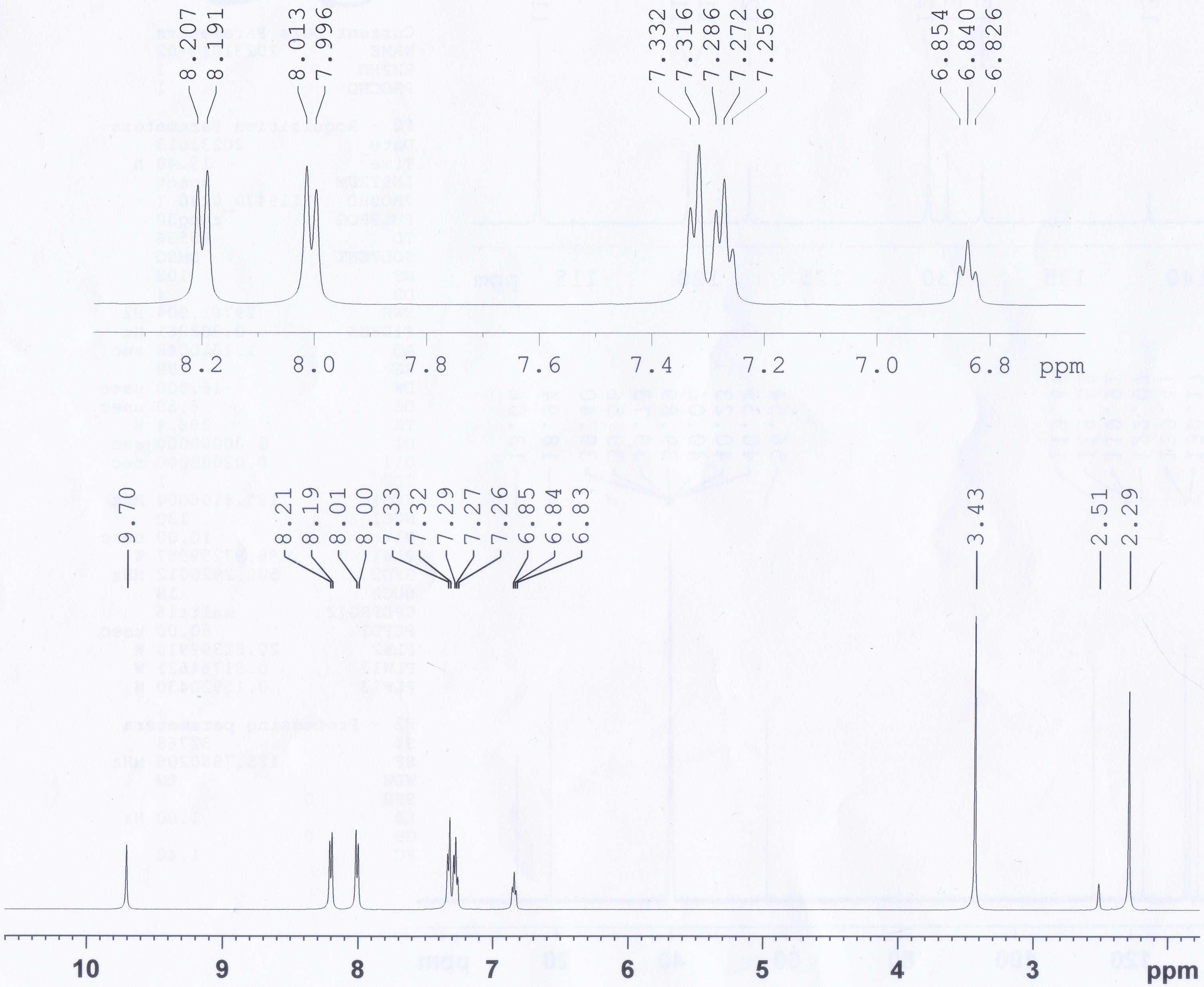

Current Data Parameters

NAME	20231013-01
EXPNO	1
PROCNO	1

F2 - Acquisition Parameters

Date_	20231013
Time	13.11 h
INSTRUM	spect
PROBHD	Z119470_0240 (
PULPROG	zg30
TD	65536
SOLVENT	DMSO
NS	16
DS	2
SWH	10000.000 Hz
FIDRES	0.305176 Hz
AQ	3.2767999 sec
RG	31.26
DW	50.000 usec
DE	6.50 usec
TE	295.6 K
D1	1.00000000 sec
TD0	1
SFO1	500.2930893 MHz
NUC1	1H
P1	10.00 usec
PLW1	20.81399918 W

F2 - Processing parameters

SI	32768
SF	500.2899972 MHz
WDW	EM
SSB	0
LB	1.00 Hz
GB	0
PC	1.40

4-API-B/DMSO-d6/Adil Saeed/Dr. Humaira Nadeem/Riphah Univ

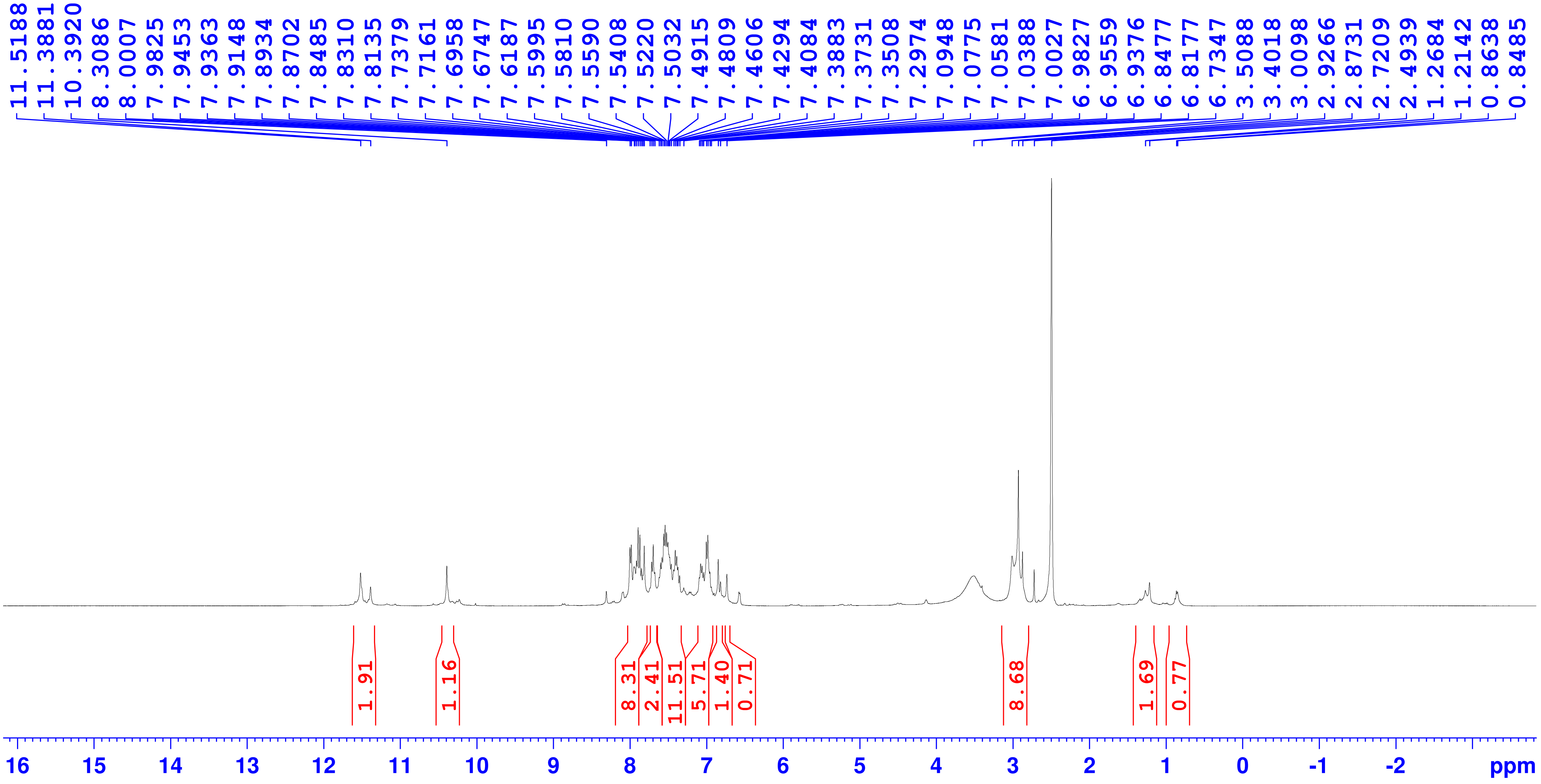

4-API-B/DMSO-d6/Adil Saeed/Dr. Humaira Nadeem/Riphah Univ

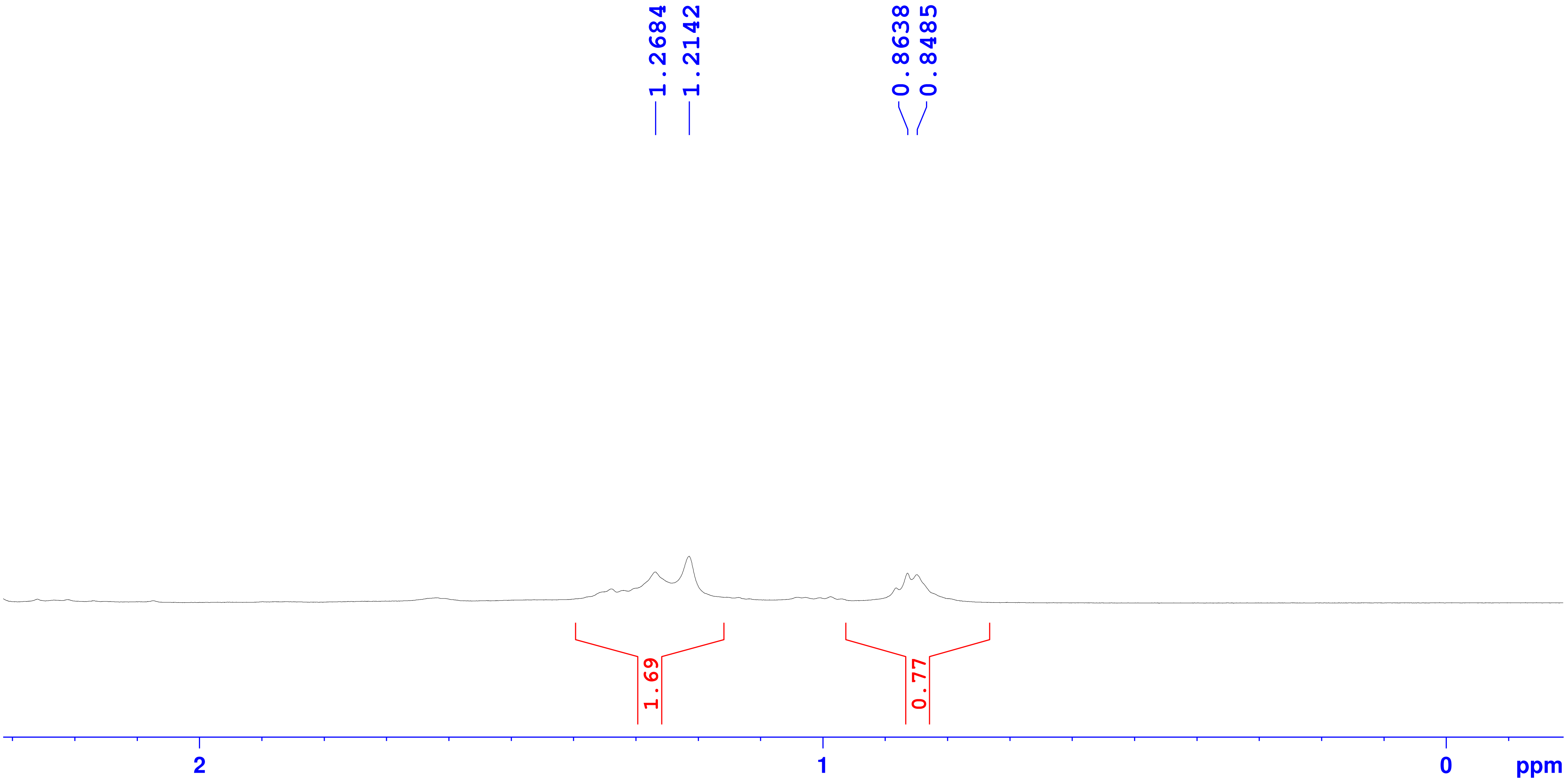

4-API-B/DMSO-d6/Adil Saeed/Dr. Humaira Nadeem/Riphah Univ

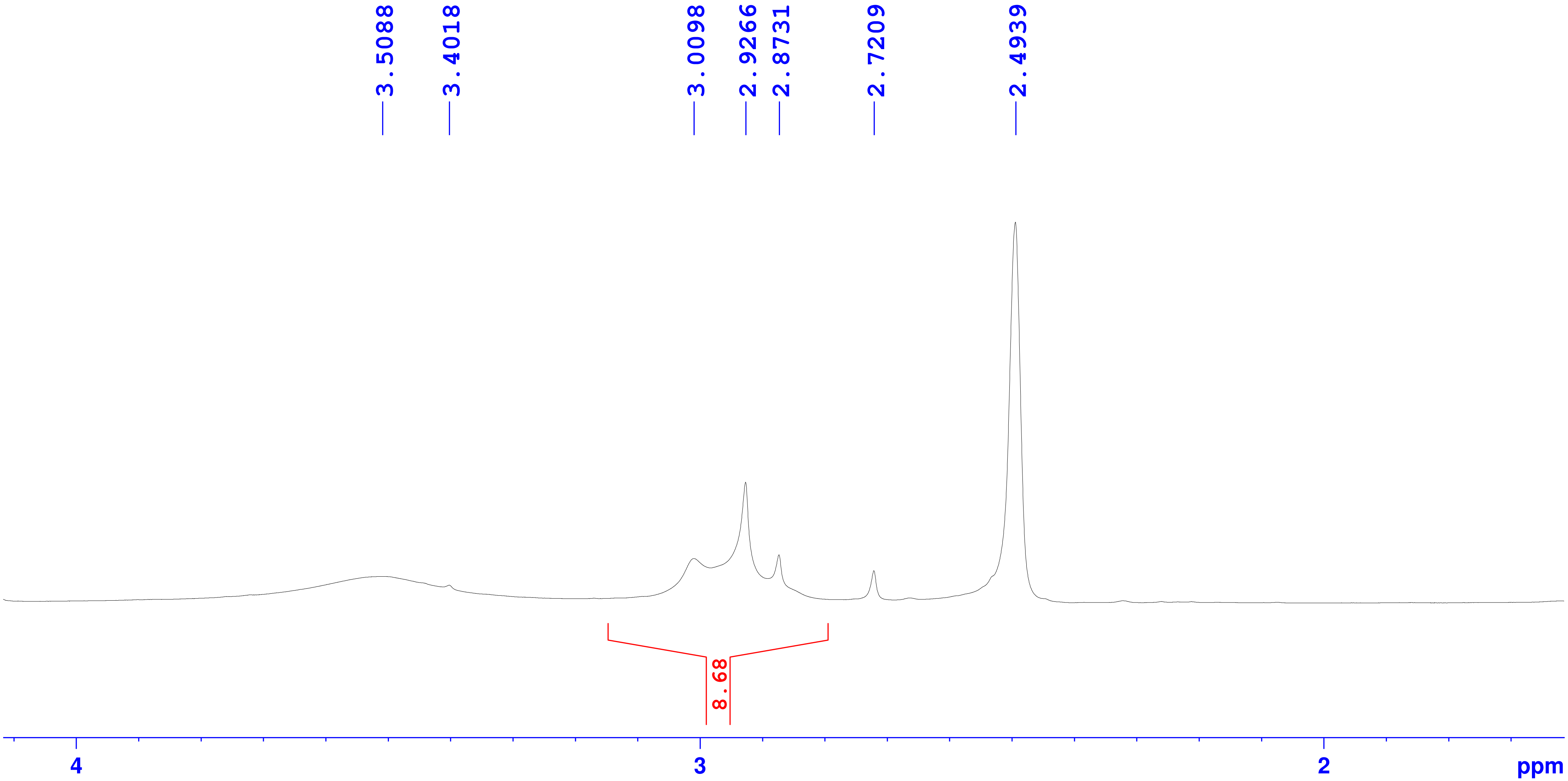

4-API-B/DMSO-d6/Adil Saeed/Dr. Humaira Nadeem/Riphah Univ

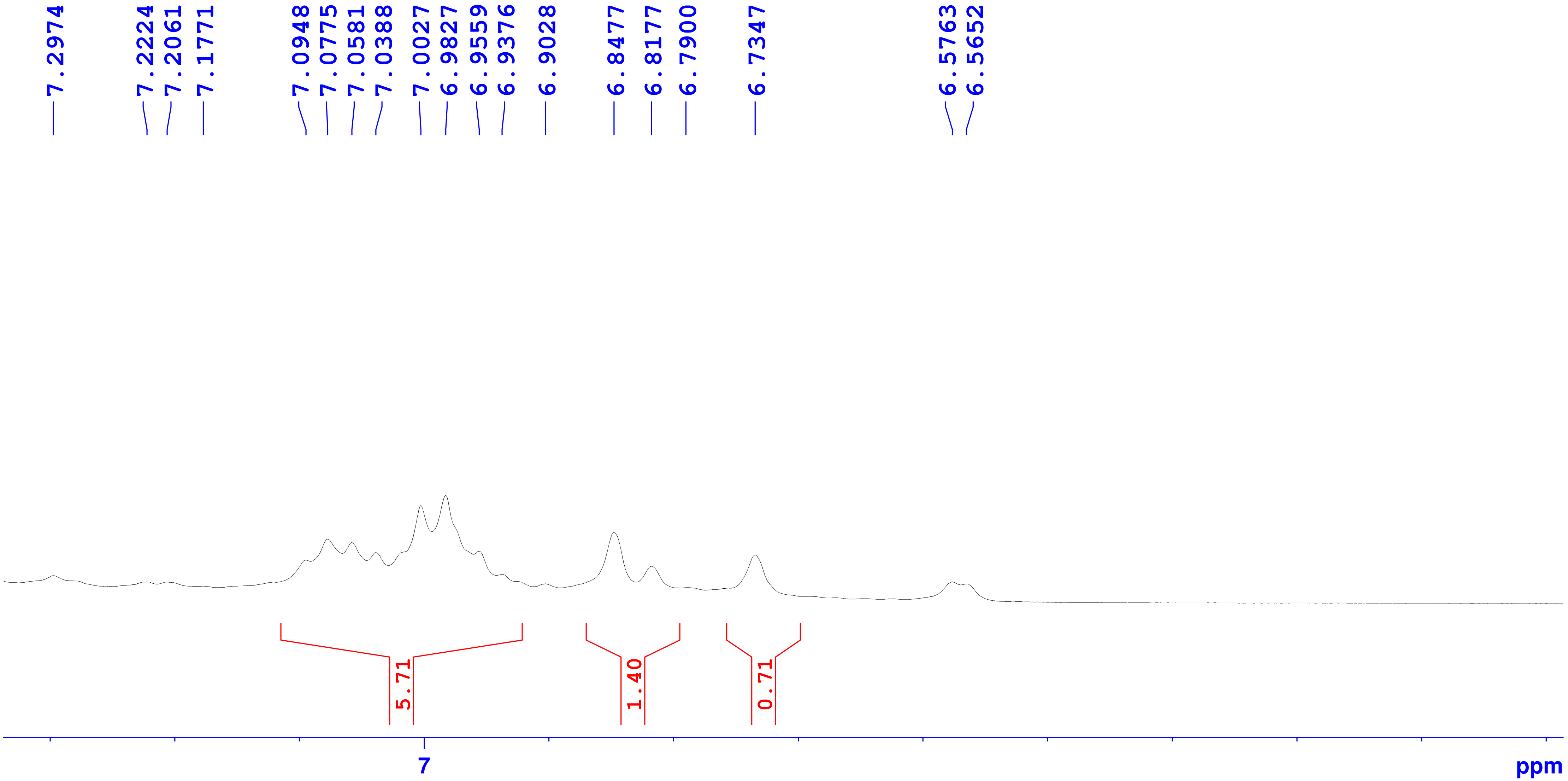

4-API-B/DMSO-d6/Adil Saeed/Dr. Humaira Nadeem/Riphah Univ

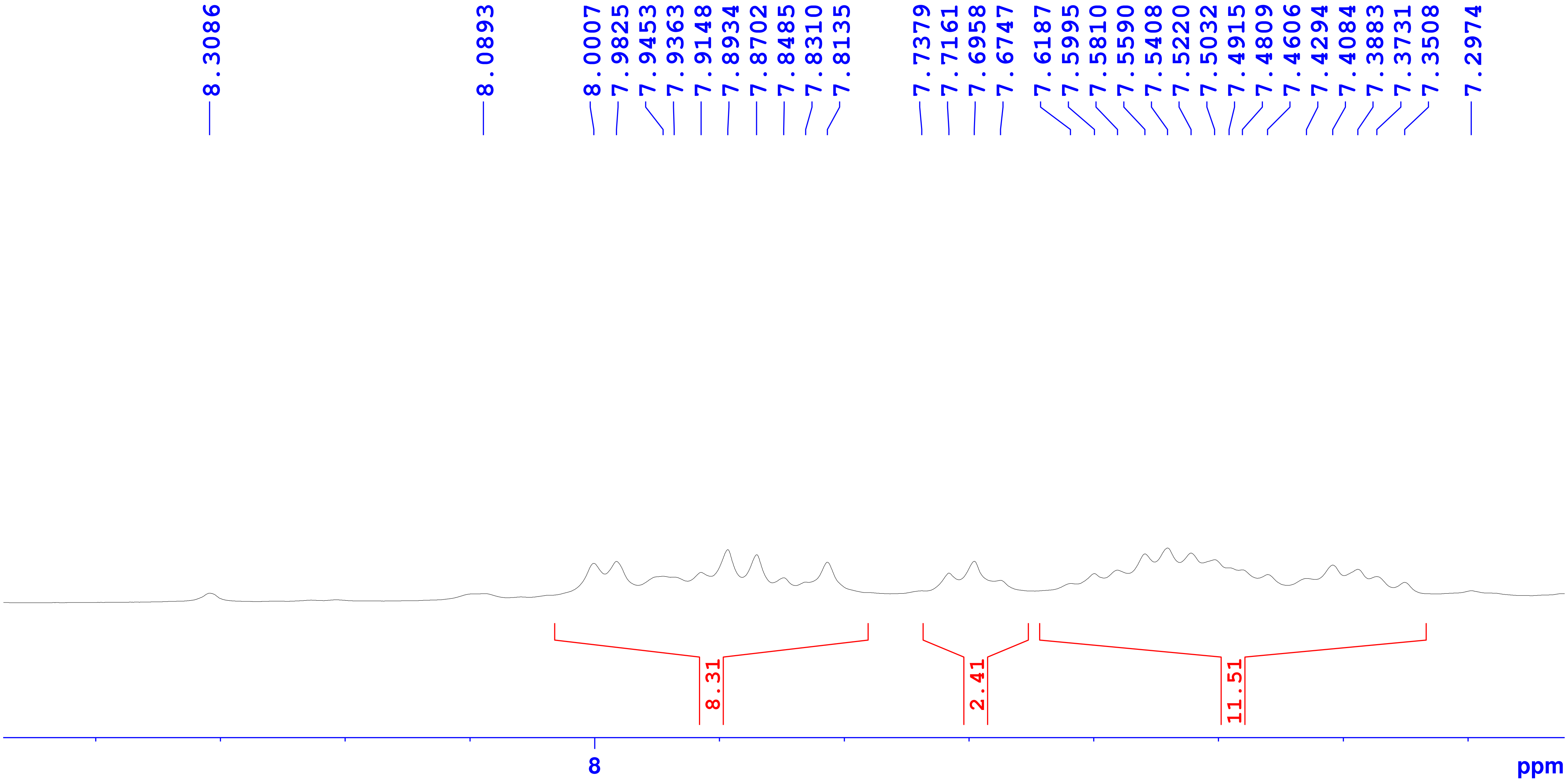

4-API-B/DMSO-d6/Adil Saeed/Dr. Humaira Nadeem/Riphah Univ

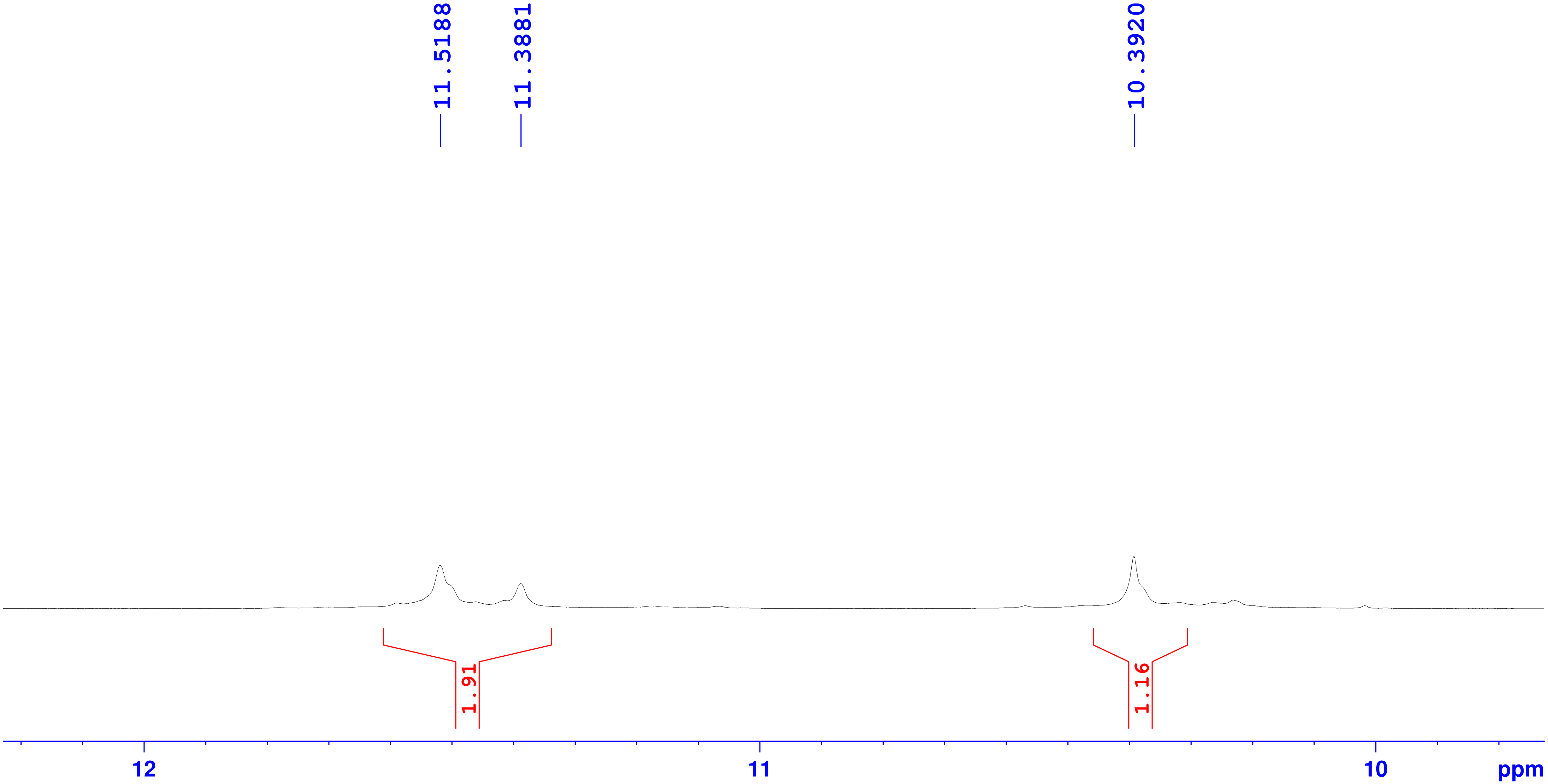

4-API-B/DMSO-d6/Adil Saeed/Dr. Humaira Nadeem/Riphah Univ

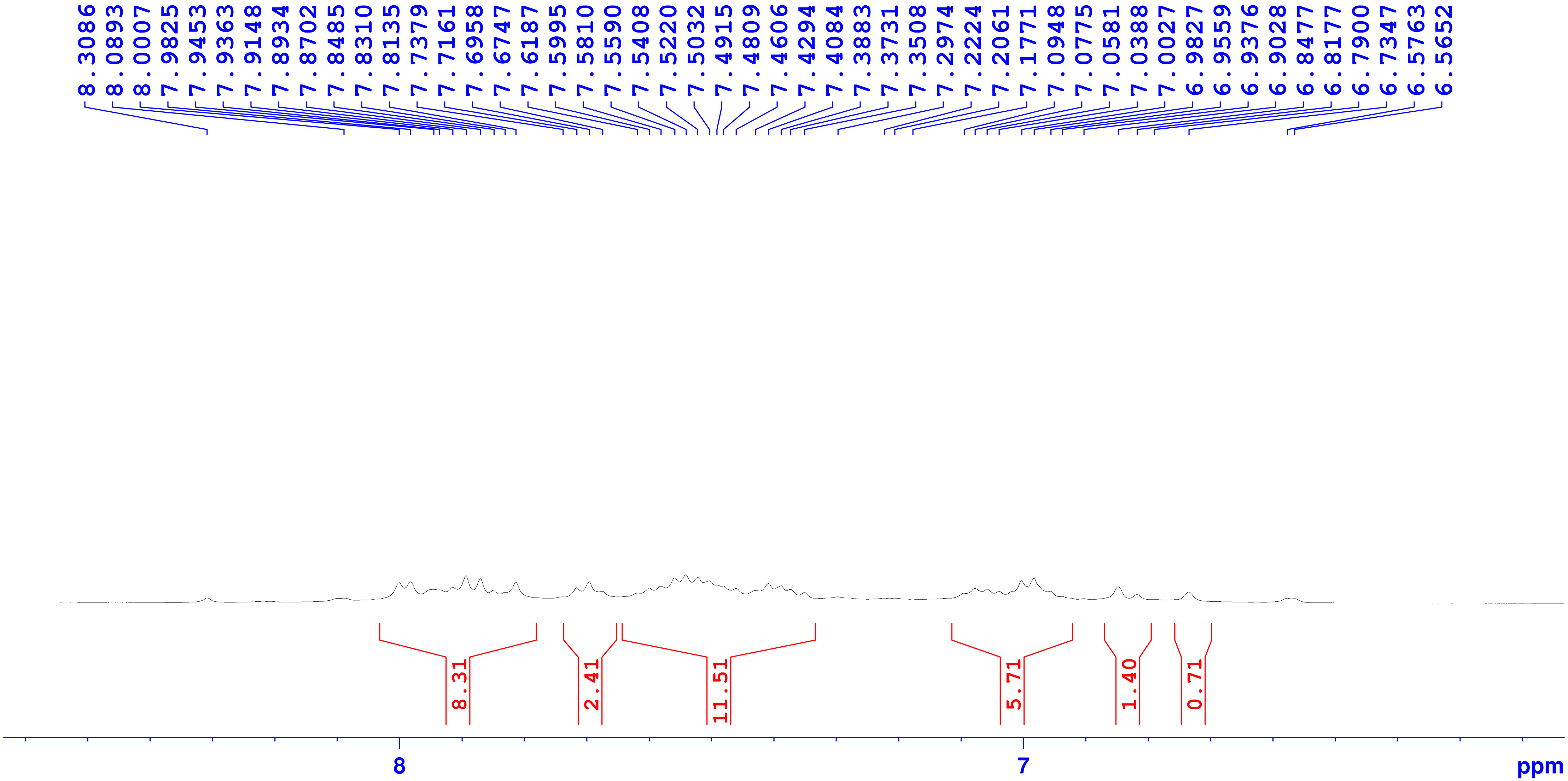

4-API-B/DMSO-d6/Adil Saeed/Dr. Humaira Nadeem/Riphah Univ

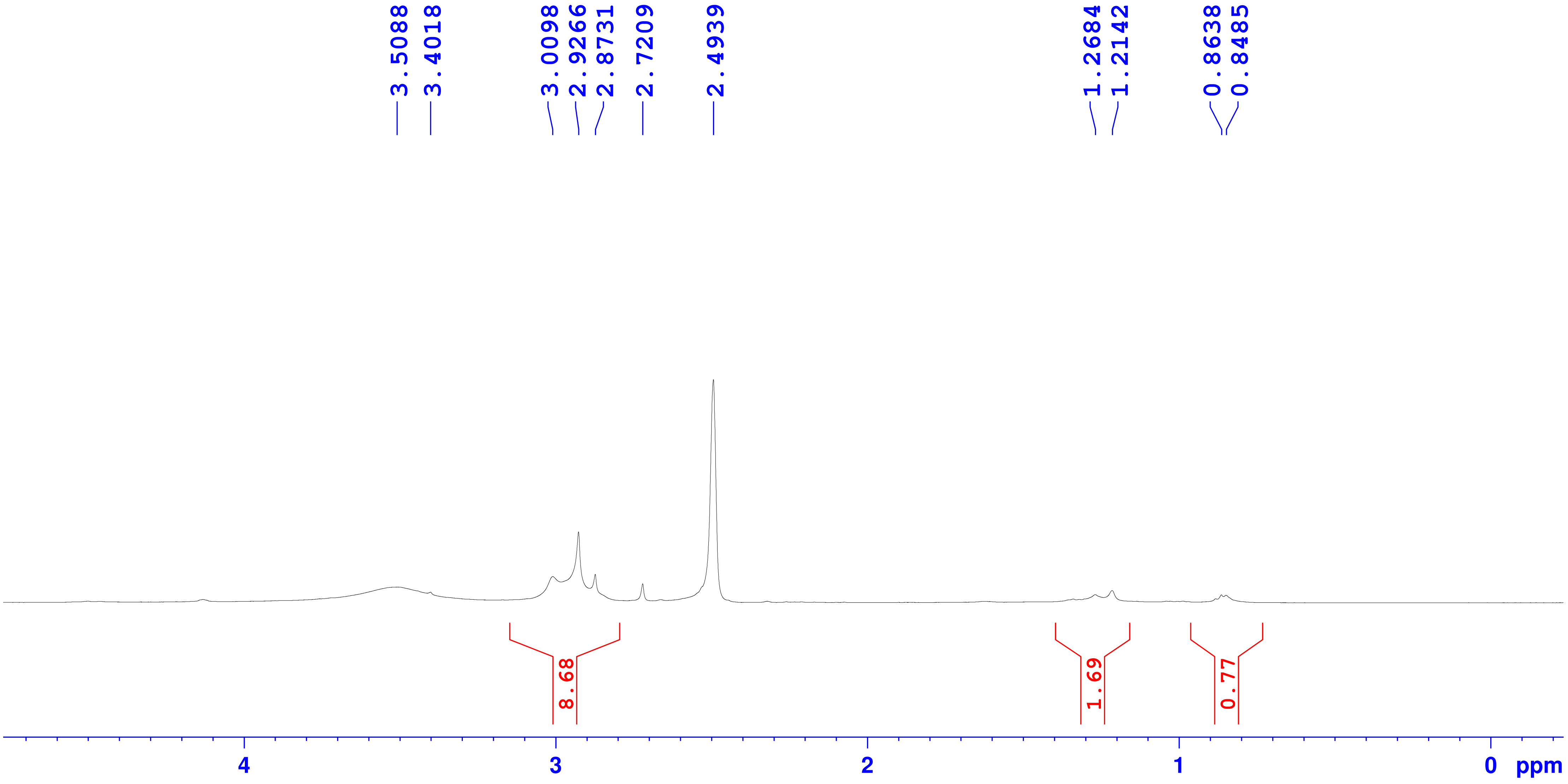

4-API-B/DMSO-d6/Adil Saeed/Dr. Humaira Nadeem/Riphah Univ

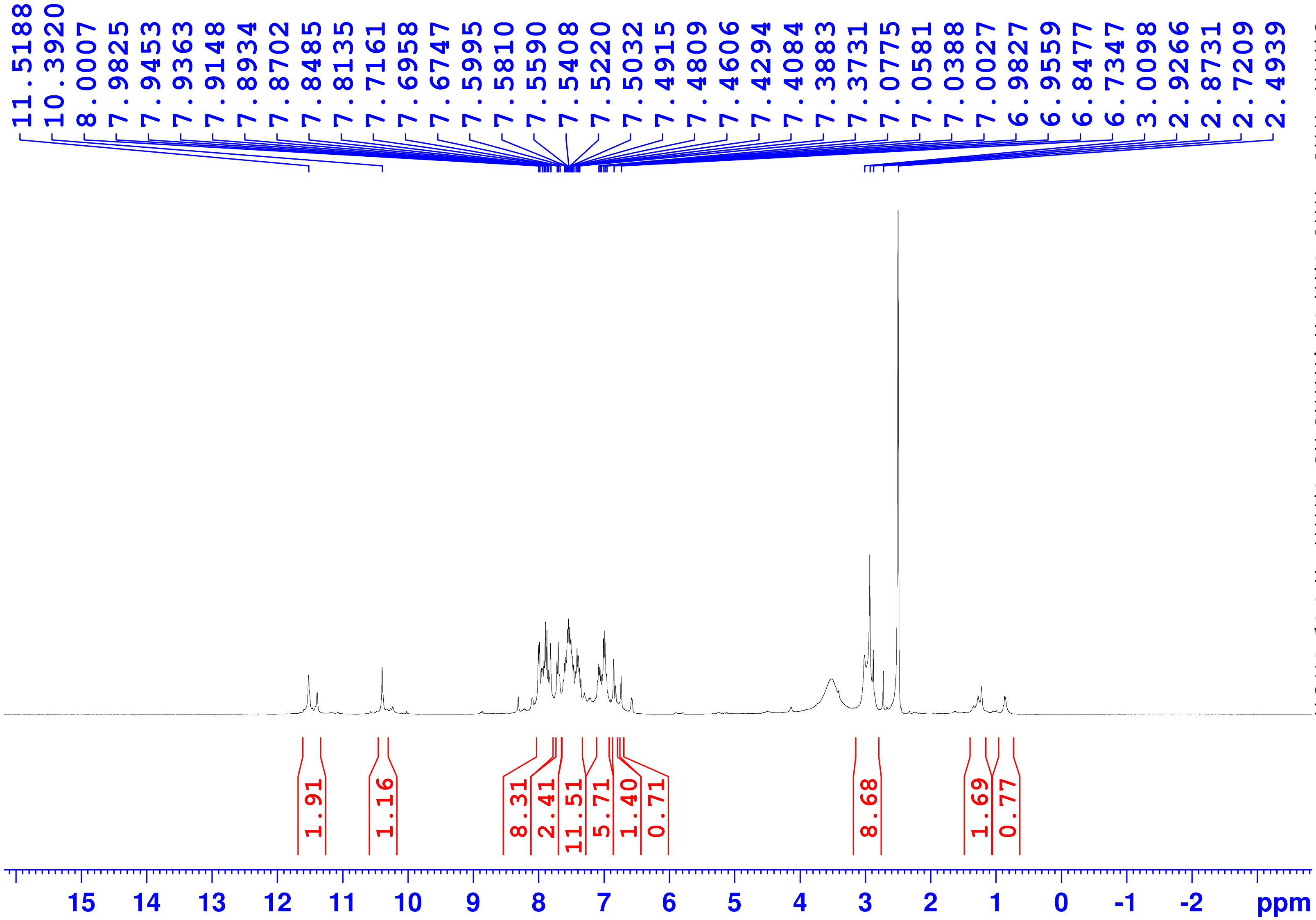

Current Data Parameters

NAME	Dr. Humaira Nadeem Riphah
EXPNO	1
PROCNO	1

F2 - Acquisition Parameters

Date_	20240611
Time	2.36 h
INSTRUM	spect
PROBHD	Z116098_0621 (
PULPROG	zg30
TD	65536
SOLVENT	DMSO
NS	16
DS	2
SWH	8012.820 Hz
FIDRES	0.244532 Hz
AQ	4.0894465 sec
RG	55.79
DW	62.400 usec
DE	6.50 usec
TE	298.0 K
D1	1.00000000 sec
TD0	1
SFO1	400.1324708 MHz
NUC1	1H
P1	10.00 usec
PLW1	16.68099976 W

F2 - Processing parameters

SI	65536
SF	400.1300000 MHz
WDW	EM
SSB	0
LB	0.30 Hz
GB	0
PC	1.00

4-APB-B/DMSO-d6/Adil Saeed/Dr. Humaira Nadeem/Riphah Univ

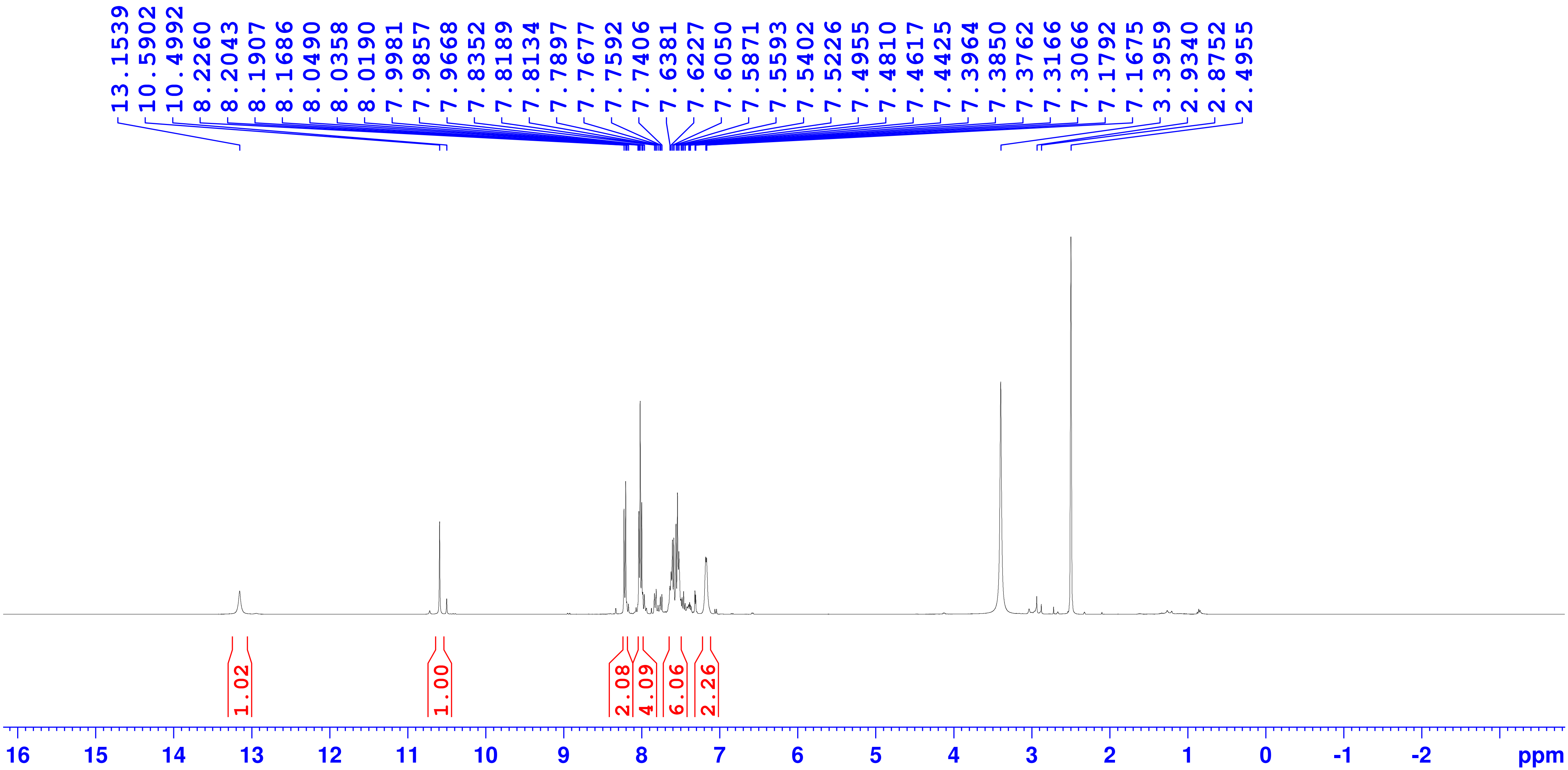

4-APB-B/DMSO-d6/Adil Saeed/Dr. Humaira Nadeem/Riphah Univ

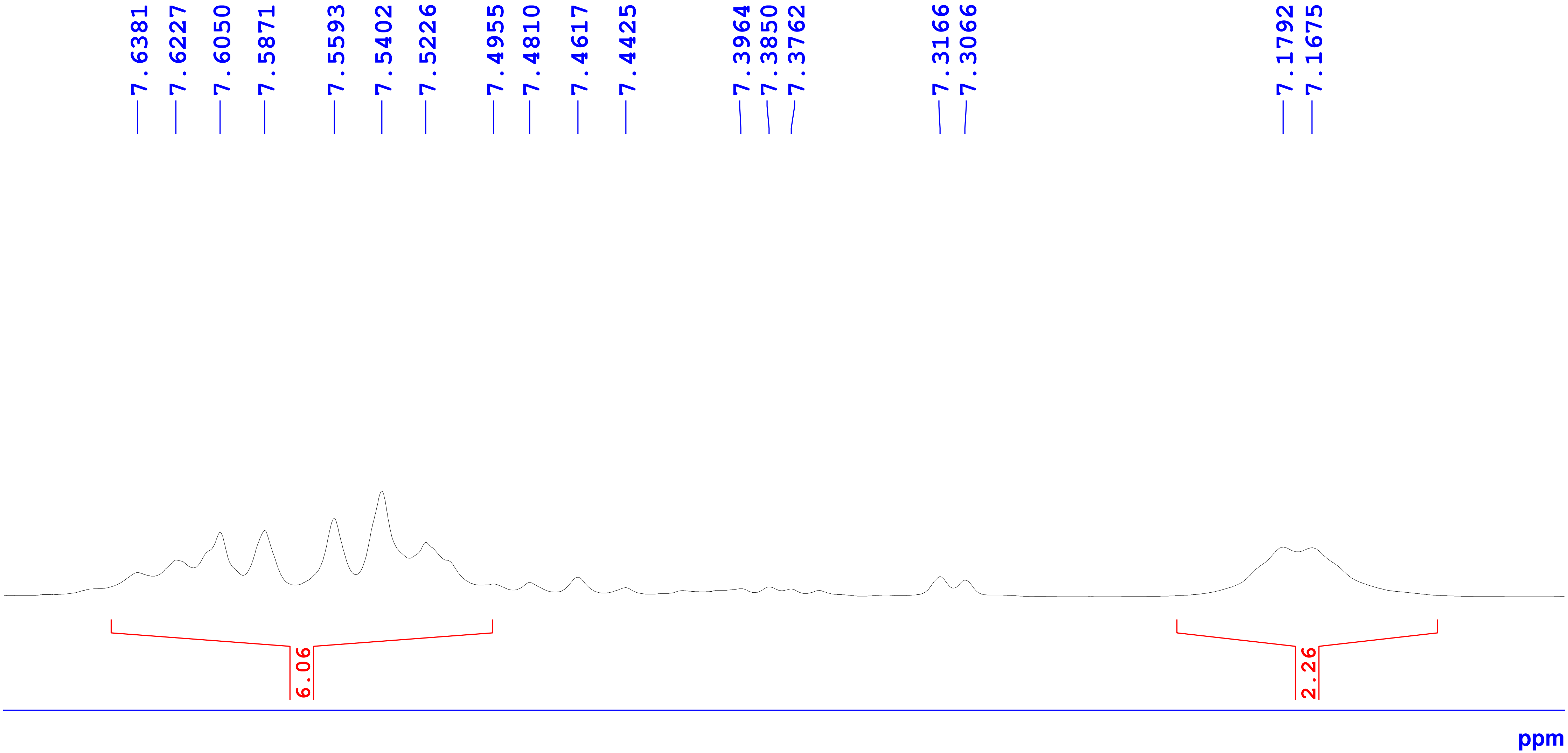

4-APB-B/DMSO-d6/Adil Saeed/Dr. Humaira Nadeem/Riphah Univ

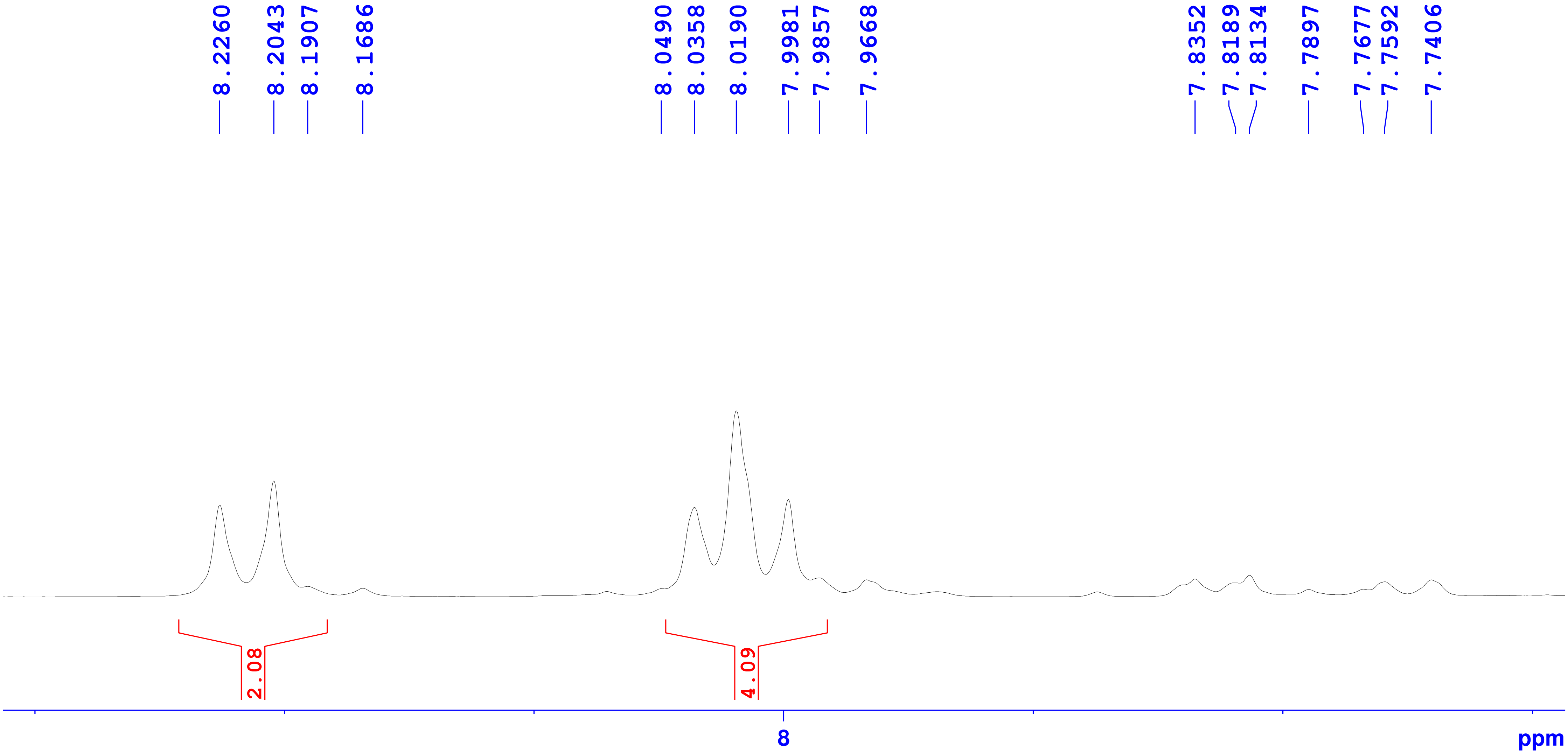

4-APB-B/DMSO-d6/Adil Saeed/Dr. Humaira Nadeem/Riphah Univ

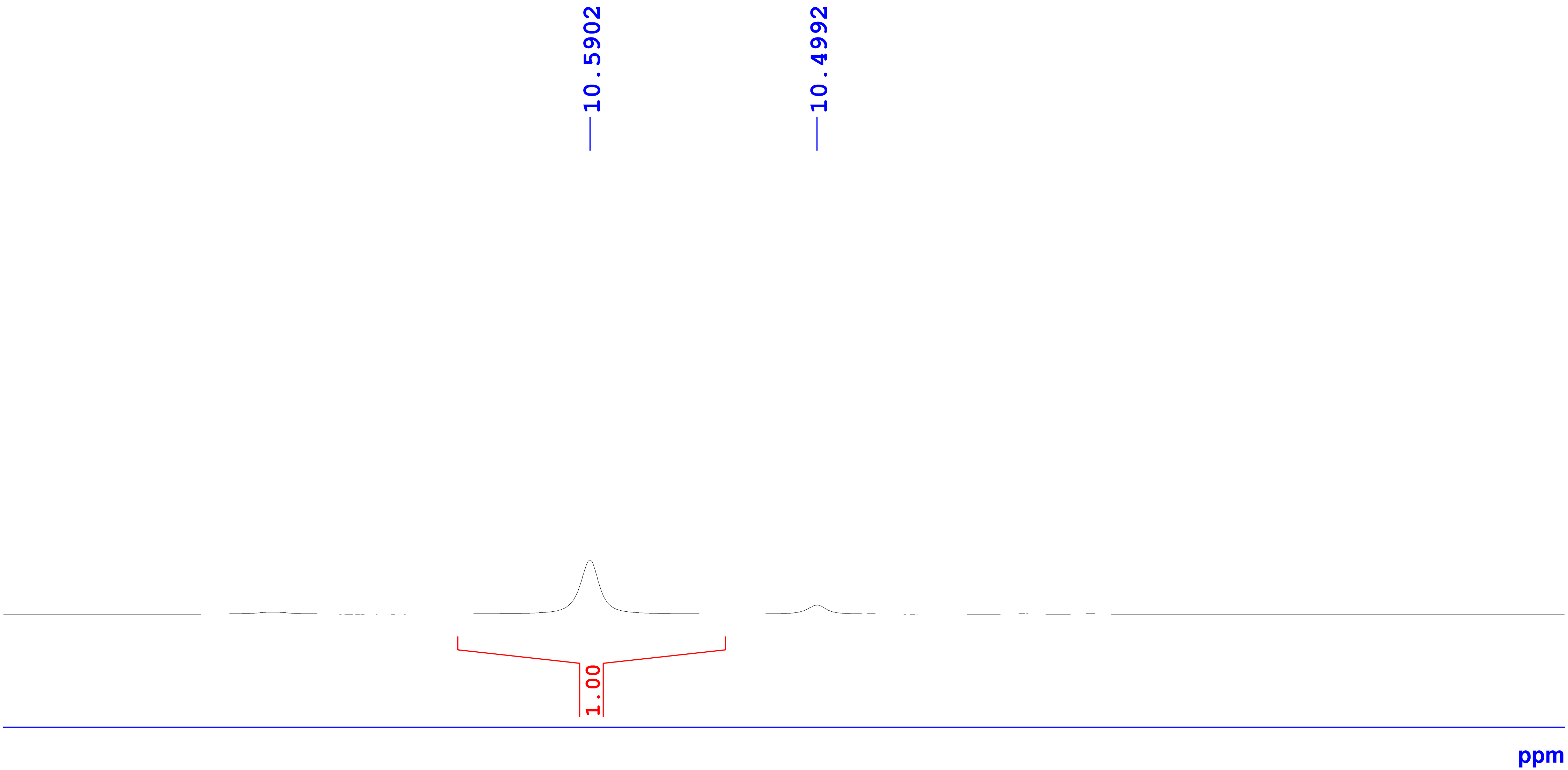

4-APB-B/DMSO-d6/Adil Saeed/Dr. Humaira Nadeem/Riphah Univ

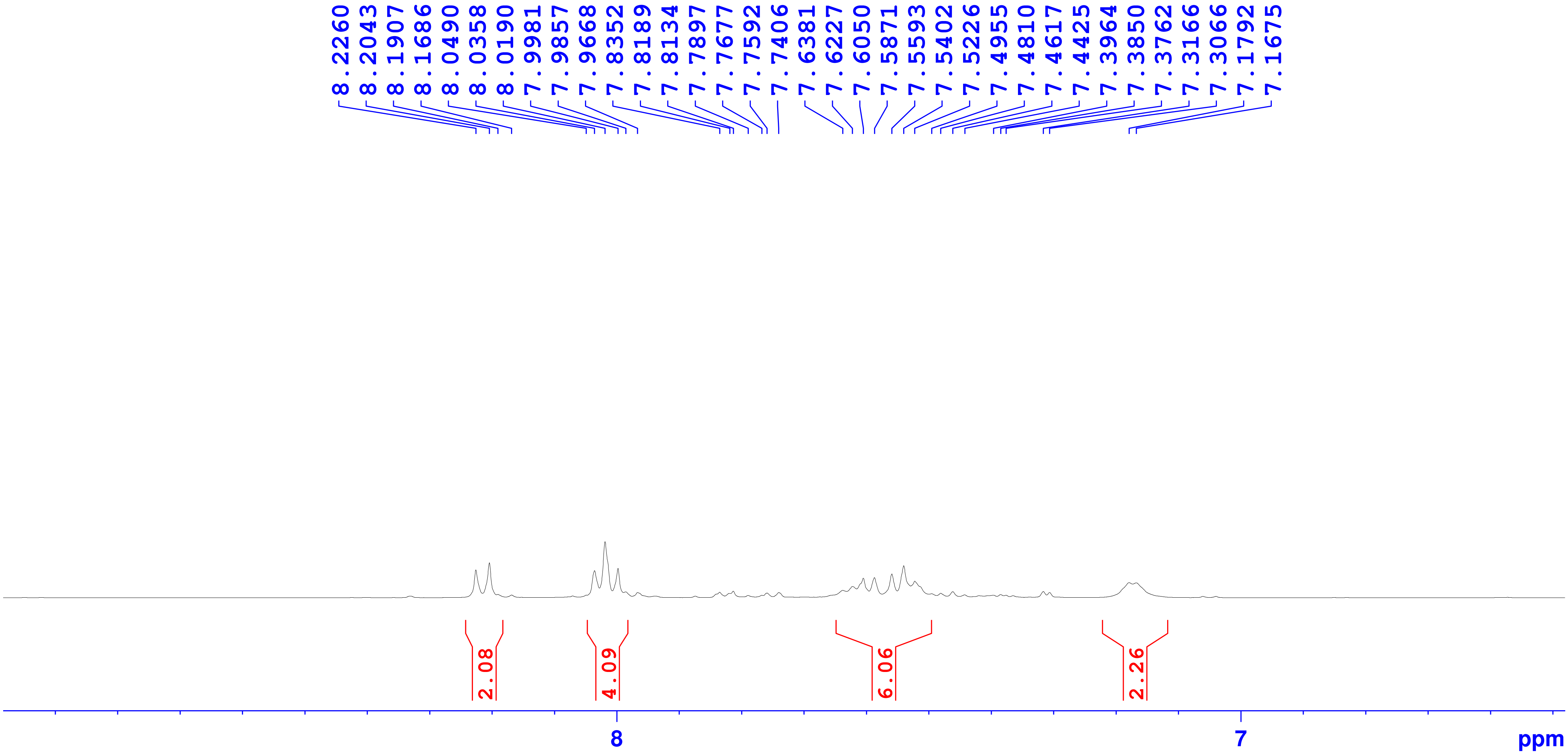

4-APB-B/DMSO-d6/Adil Saeed/Dr. Humaira Nadeem/Riphah Univ

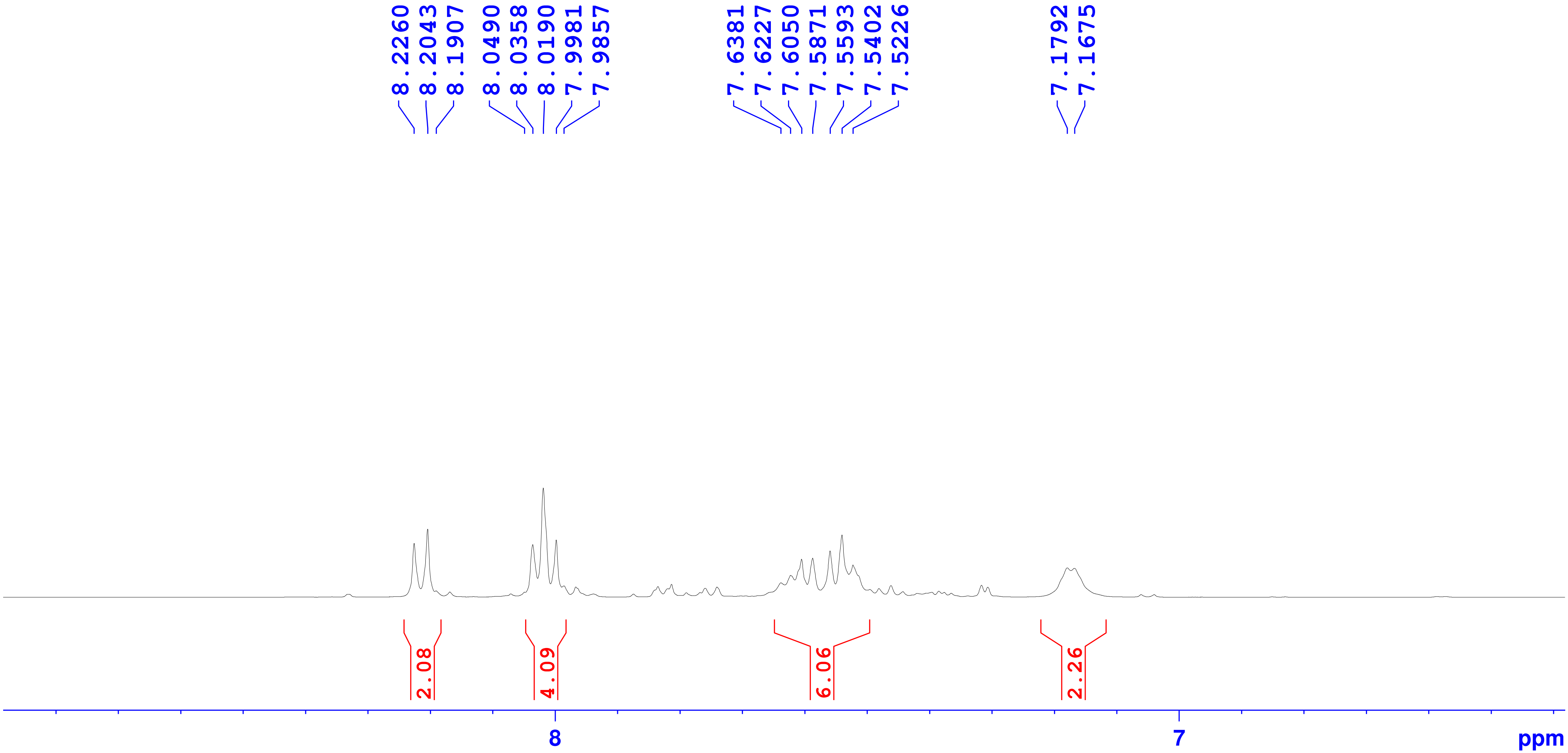

4-APB-B/DMSO-d6/Adil Saeed/Dr. Humaira Nadeem/Riphah Univ

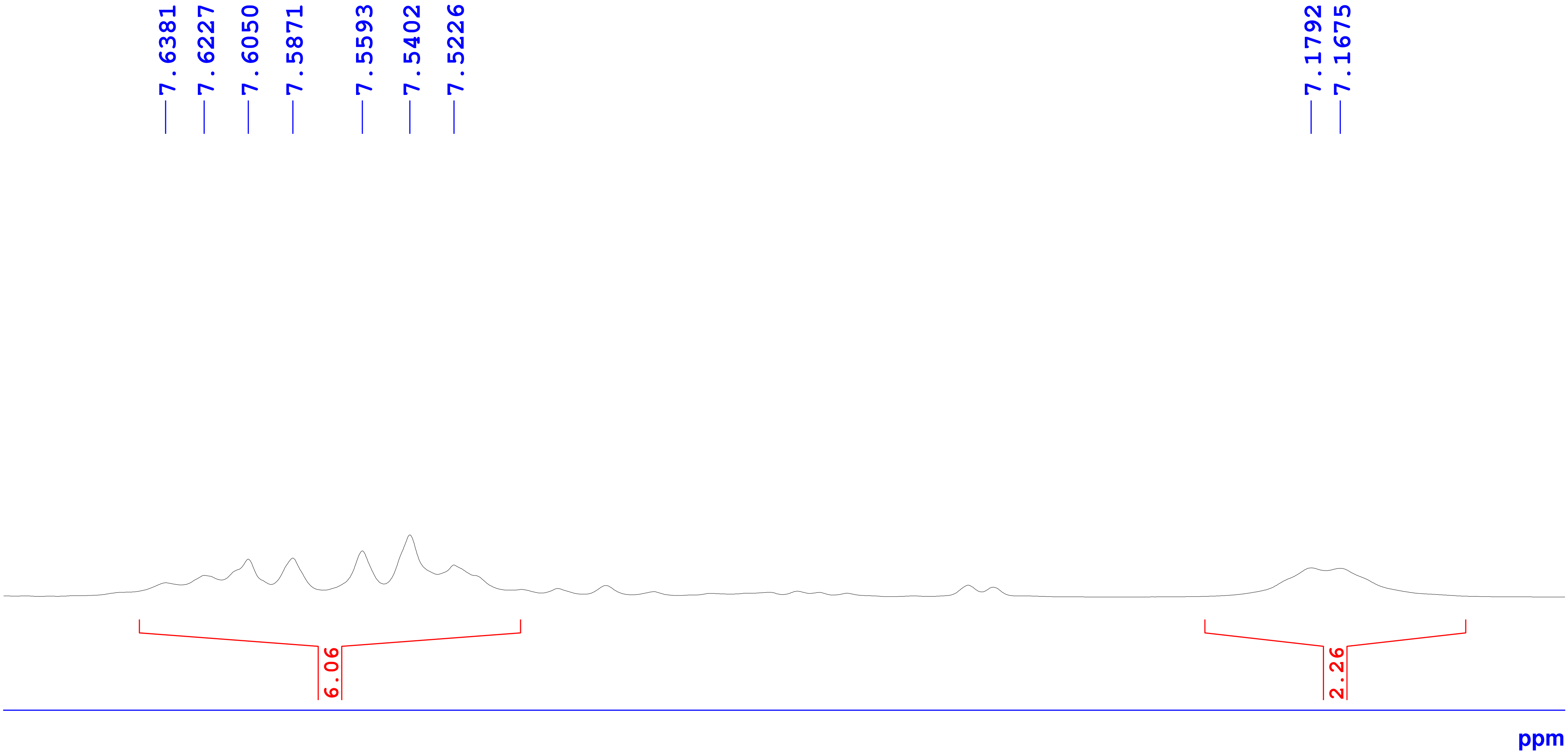

4-APB-B/DMSO-d6/Adil Saeed/Dr. Humaira Nadeem/Riphah Univ

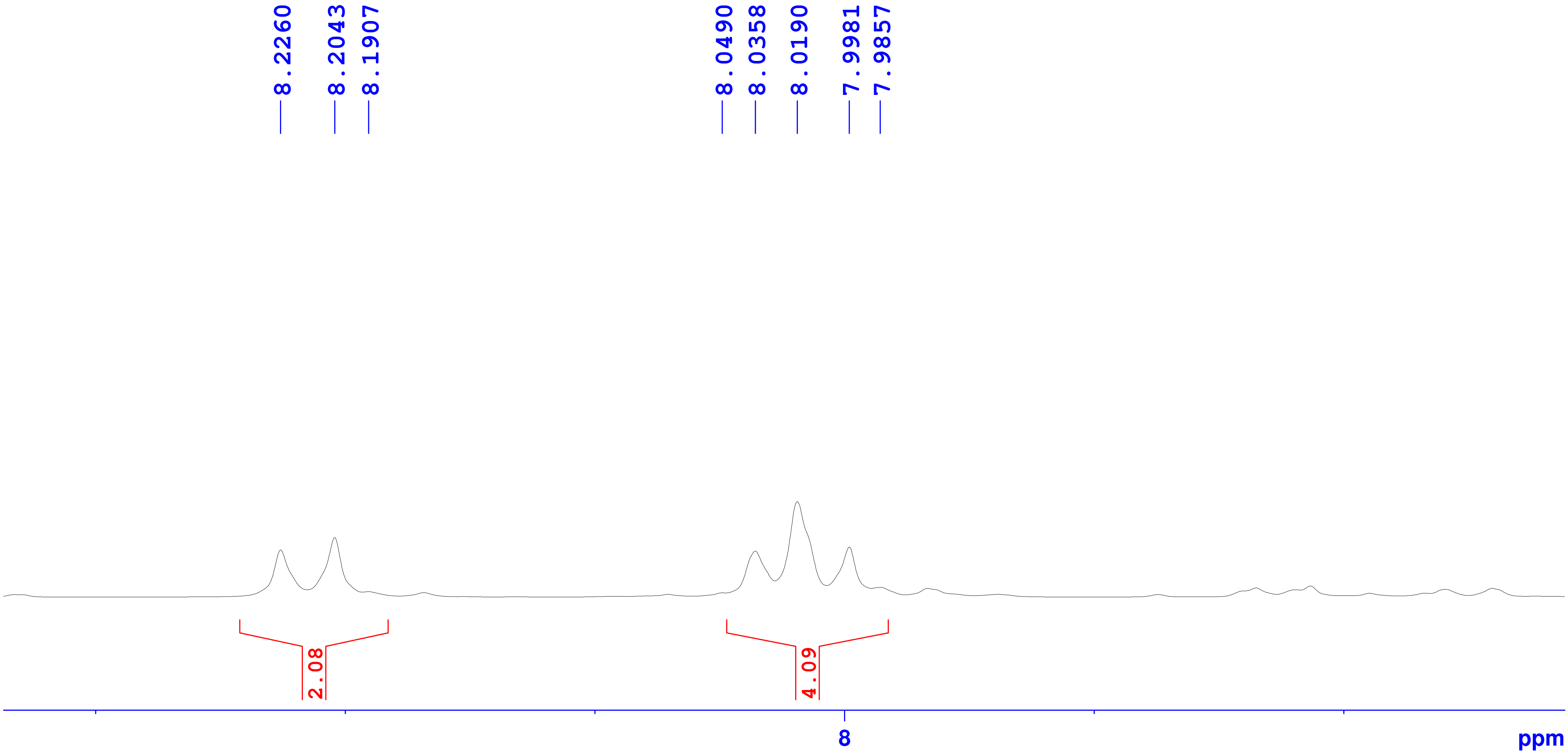

4-APB-B/DMSO-d6/Adil Saeed/Dr. Humaira Nadeem/Riphah Univ

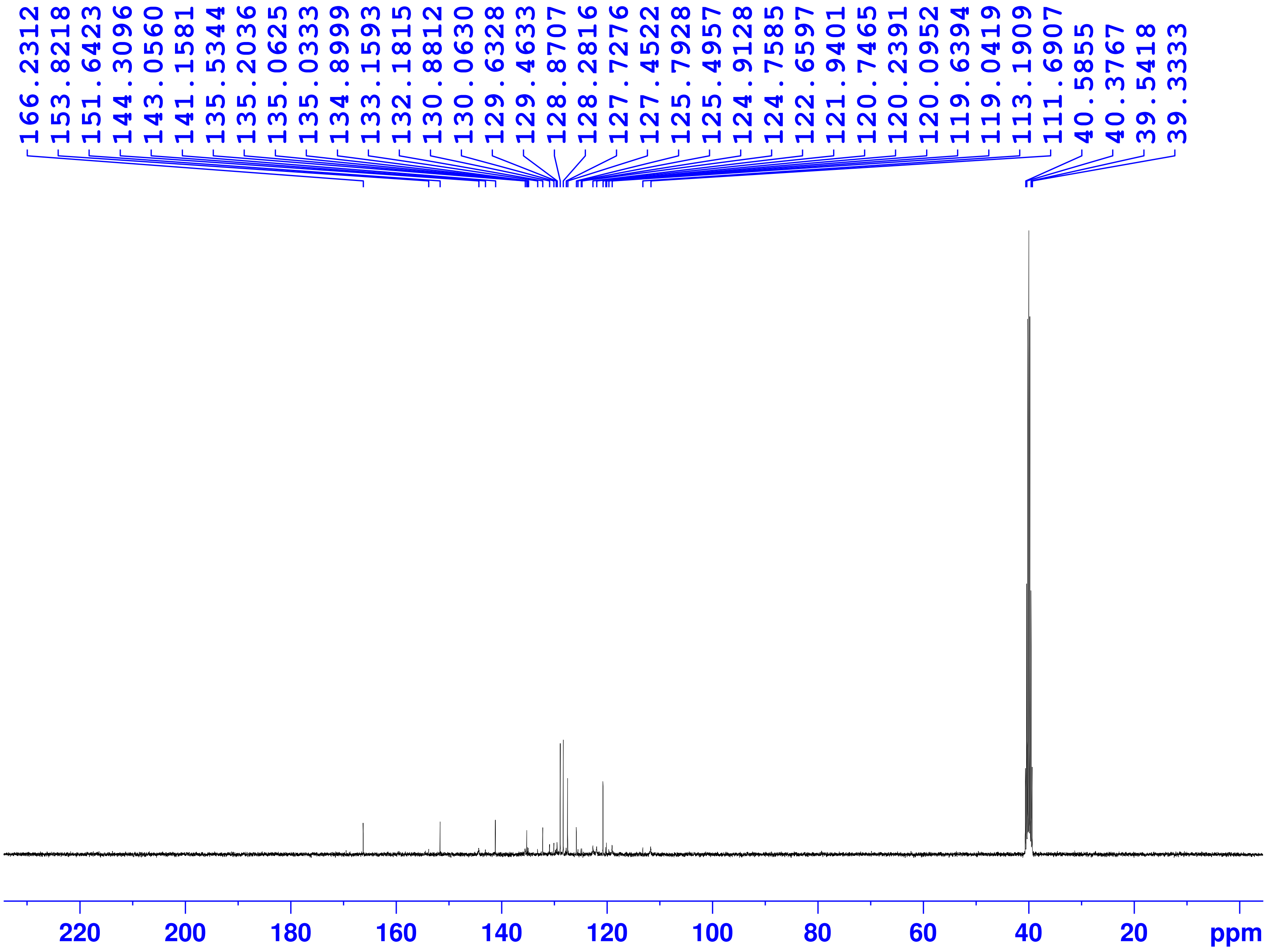

BRUKER

Current Data Parameters

NAME	Dr. Humaira Nadeem Riphah
EXPNO	4
PROCNO	1

F2 - Acquisition Parameters

Date_	20240611
Time	3.28 h
INSTRUM	spect
PROBHD	Z116098_0621 (
PULPROG	zgpg30
TD	65536
SOLVENT	DMSO
NS	400
DS	2
SWH	24038.461 Hz
FIDRES	0.733596 Hz
AQ	1.3631488 sec
RG	199.48
DW	20.800 usec
DE	6.50 usec
TE	298.0 K
D1	2.00000000 sec
D11	0.03000000 sec
TD0	1
SFO1	100.6243390 MHz
NUC1	13C
P1	10.00 usec
PLW1	72.56700134 W
SFO2	400.1316005 MHz
NUC2	1H
CPDPRG[2	waltz16
PCPD2	90.00 usec
PLW2	16.68099976 W
PLW12	0.20593999 W
PLW13	0.10342000 W

F2 - Processing parameters

SI	32768
SF	100.6127685 MHz
WDW	EM
SSB	0
LB	1.00 Hz
GB	0
PC	1.40

4-APB-B/DMSO-d6/Adil Saeed/Dr. Humaira Nadeem/Riphah Univ

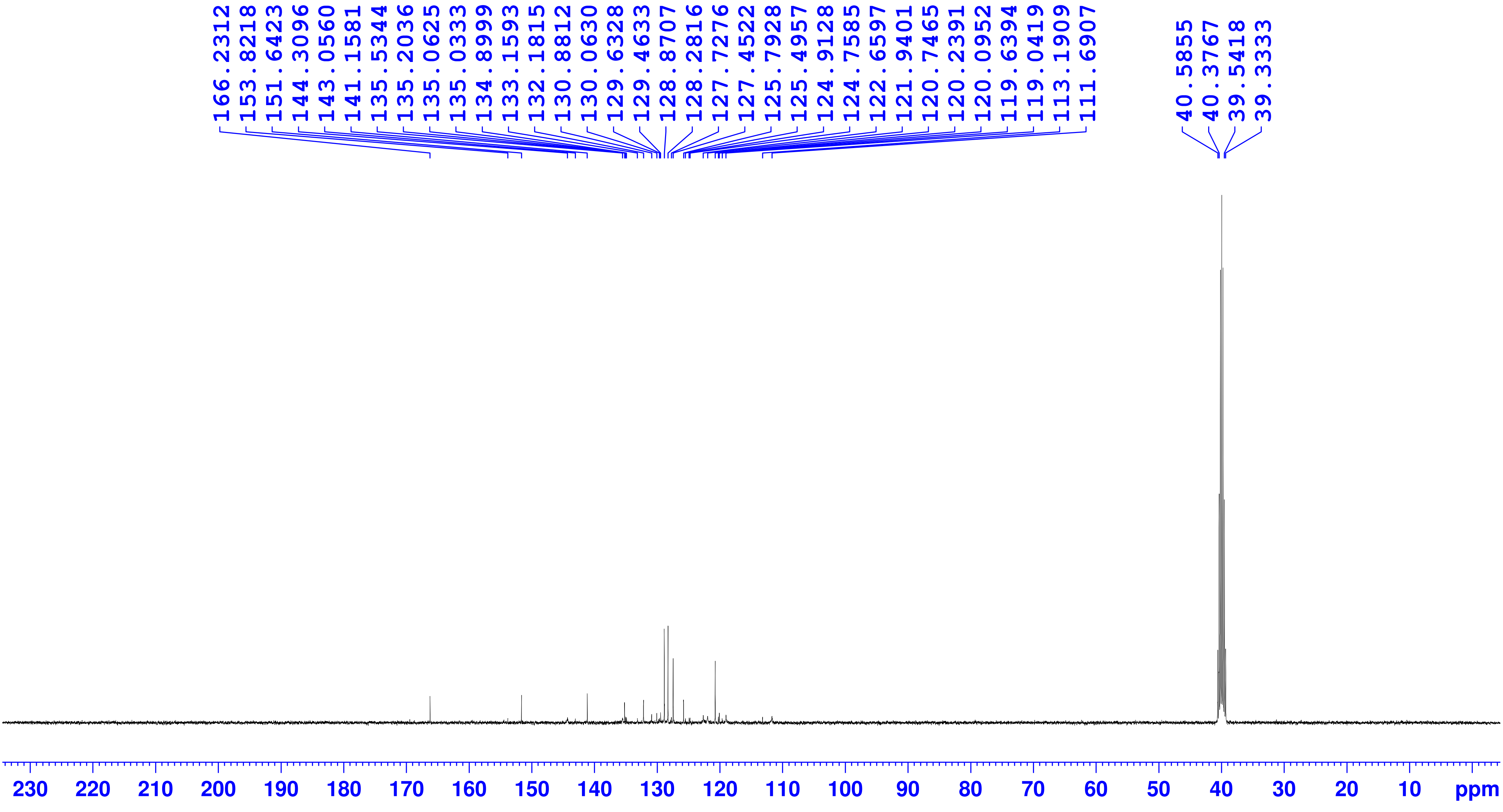

4-APB-B/DMSO-d6/Adil Saeed/Dr. Humaira Nadeem/Riphah Univ

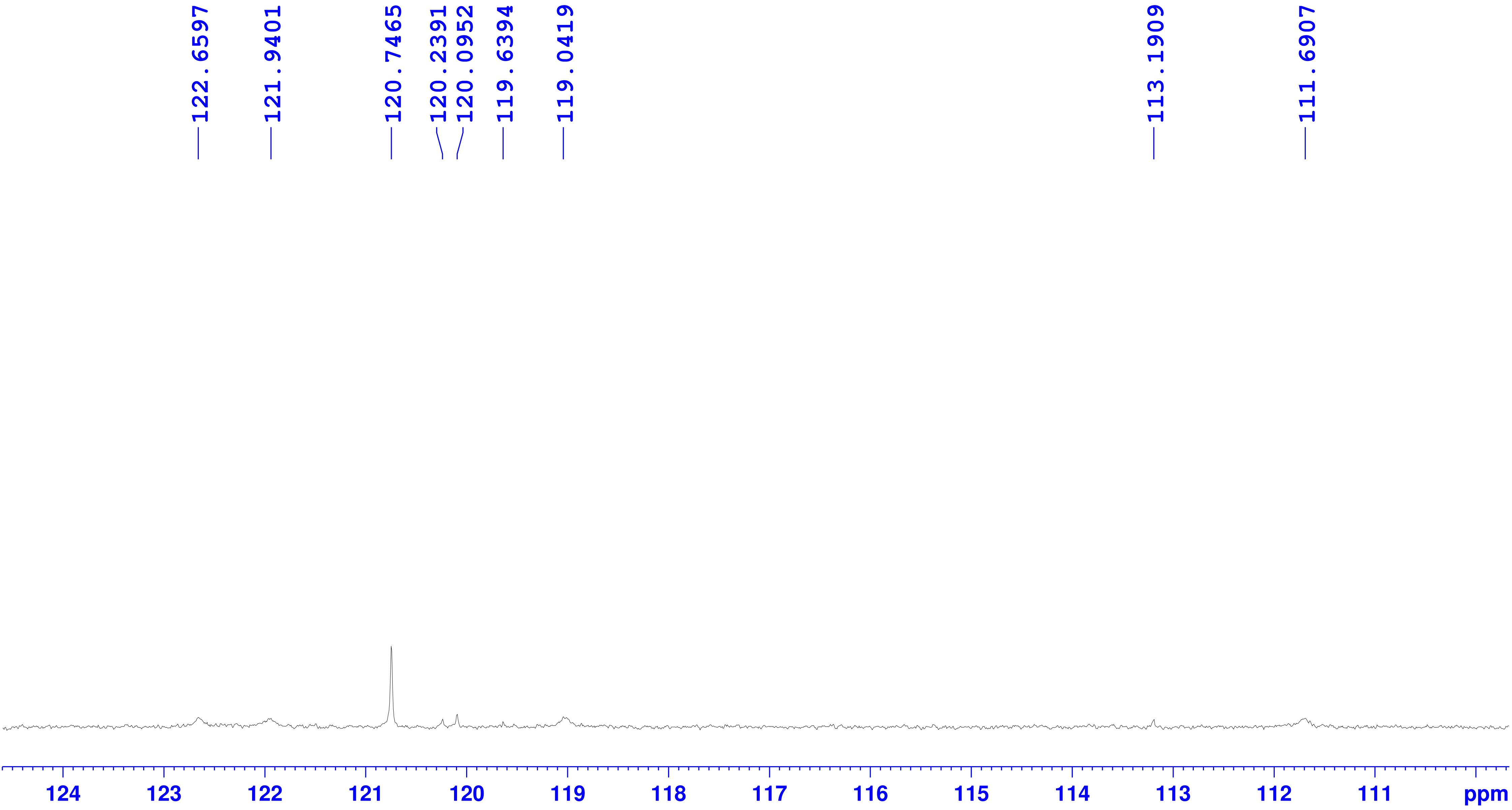

4-APB-B/DMSO-d6/Adil Saeed/Dr. Humaira Nadeem/Riphah Univ

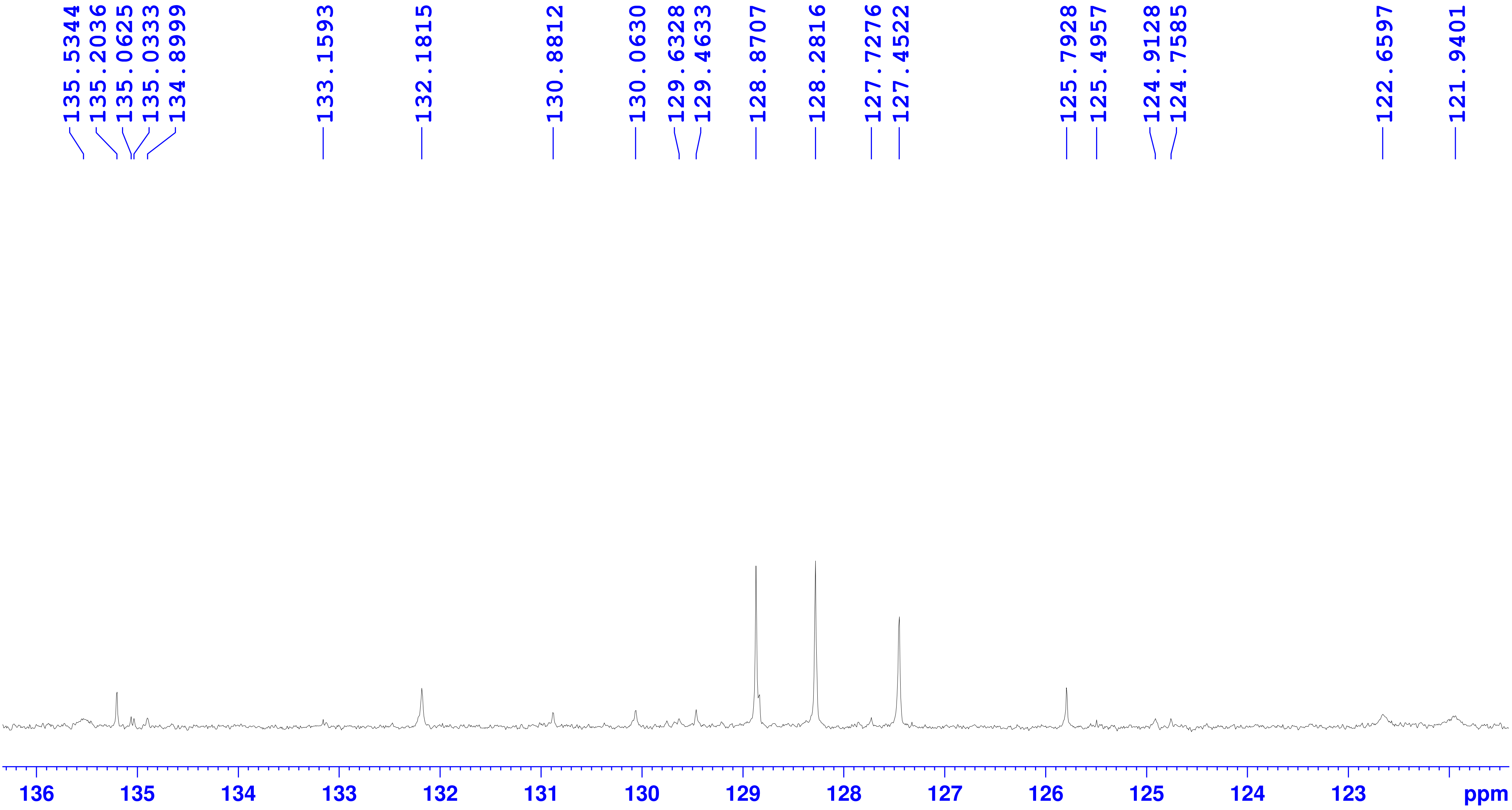

4-APB-B/DMSO-d6/Adil Saeed/Dr. Humaira Nadeem/Riphah Univ

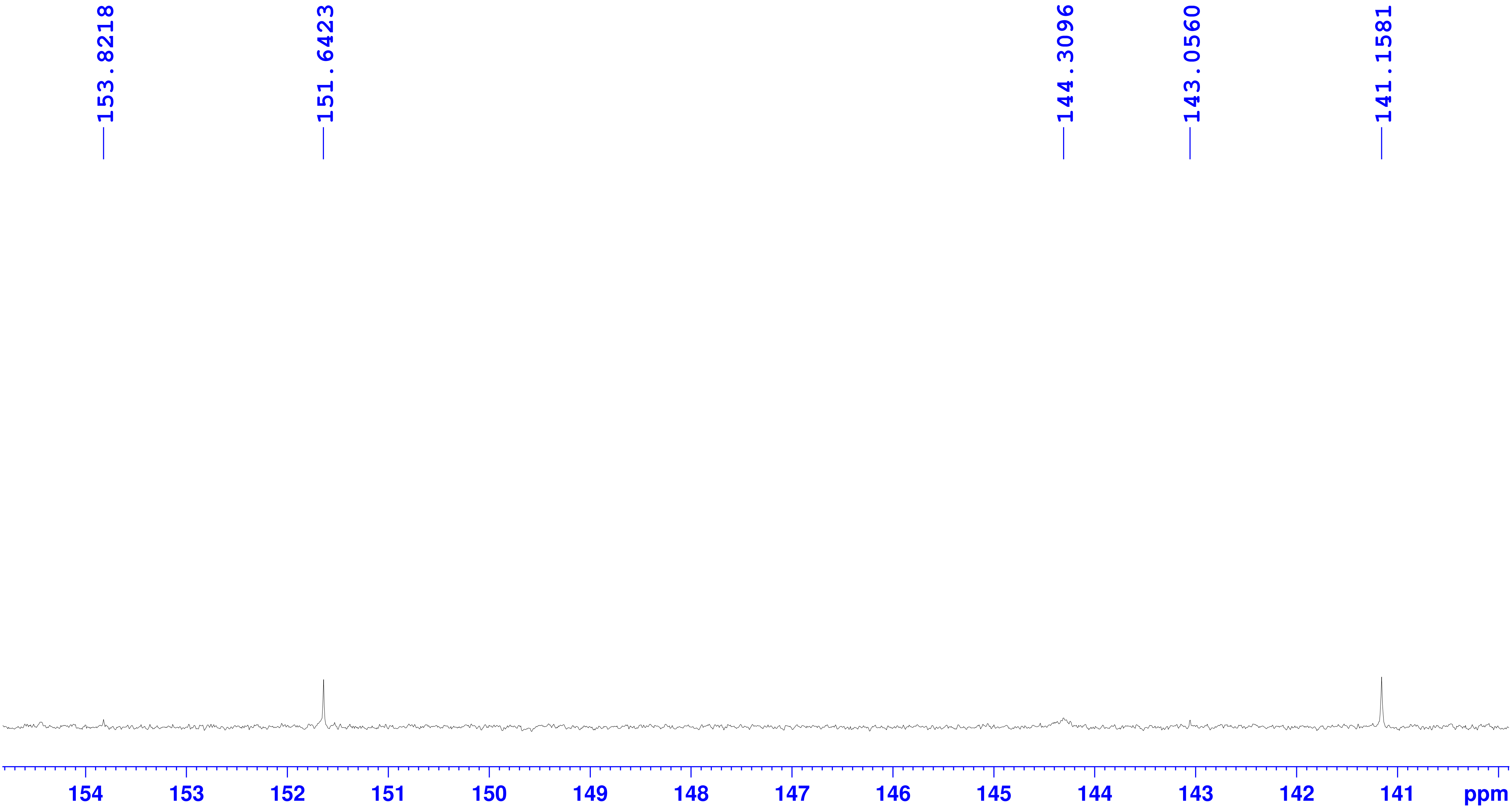

4-APB-B/DMSO-d6/Adil Saeed/Dr. Humaira Nadeem/Riphah Univ

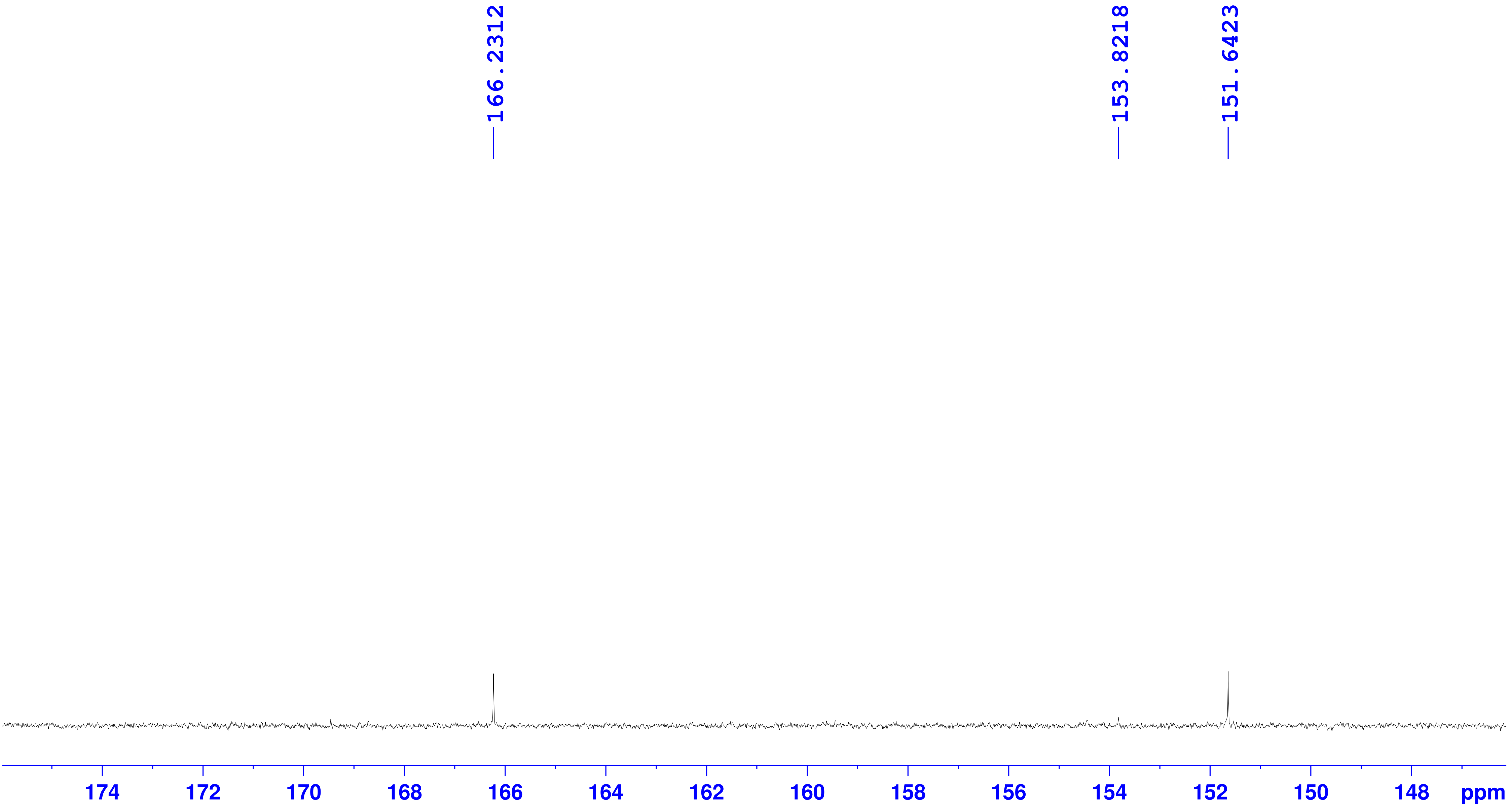

4-APB-B/DMSO-d6/Adil Saeed/Dr. Humaira Nadeem/Riphah Univ

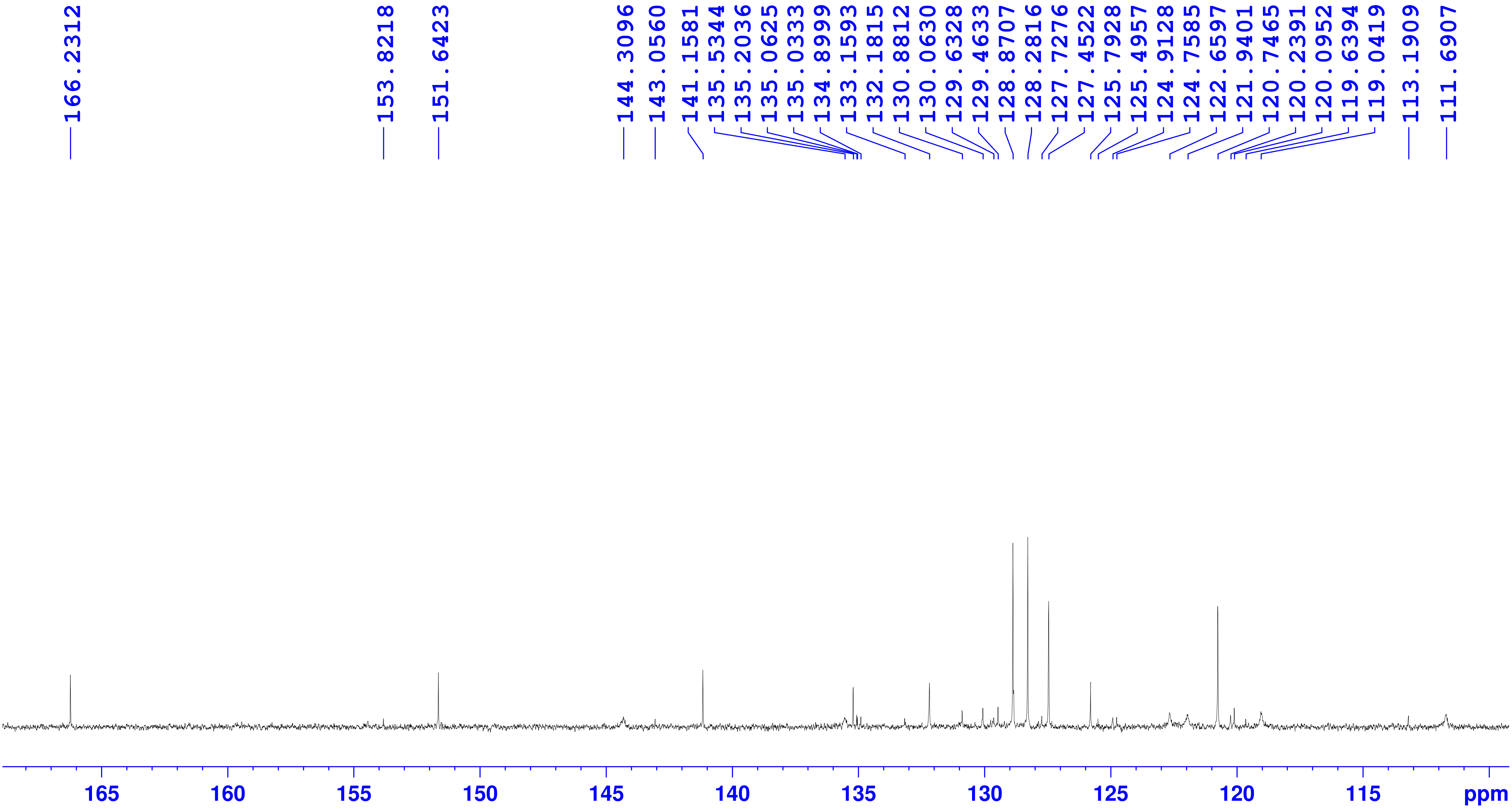

4-API-B/DMSO-d6/Adil Saeed/Dr. Humaira Nadeem/Riphah Univ

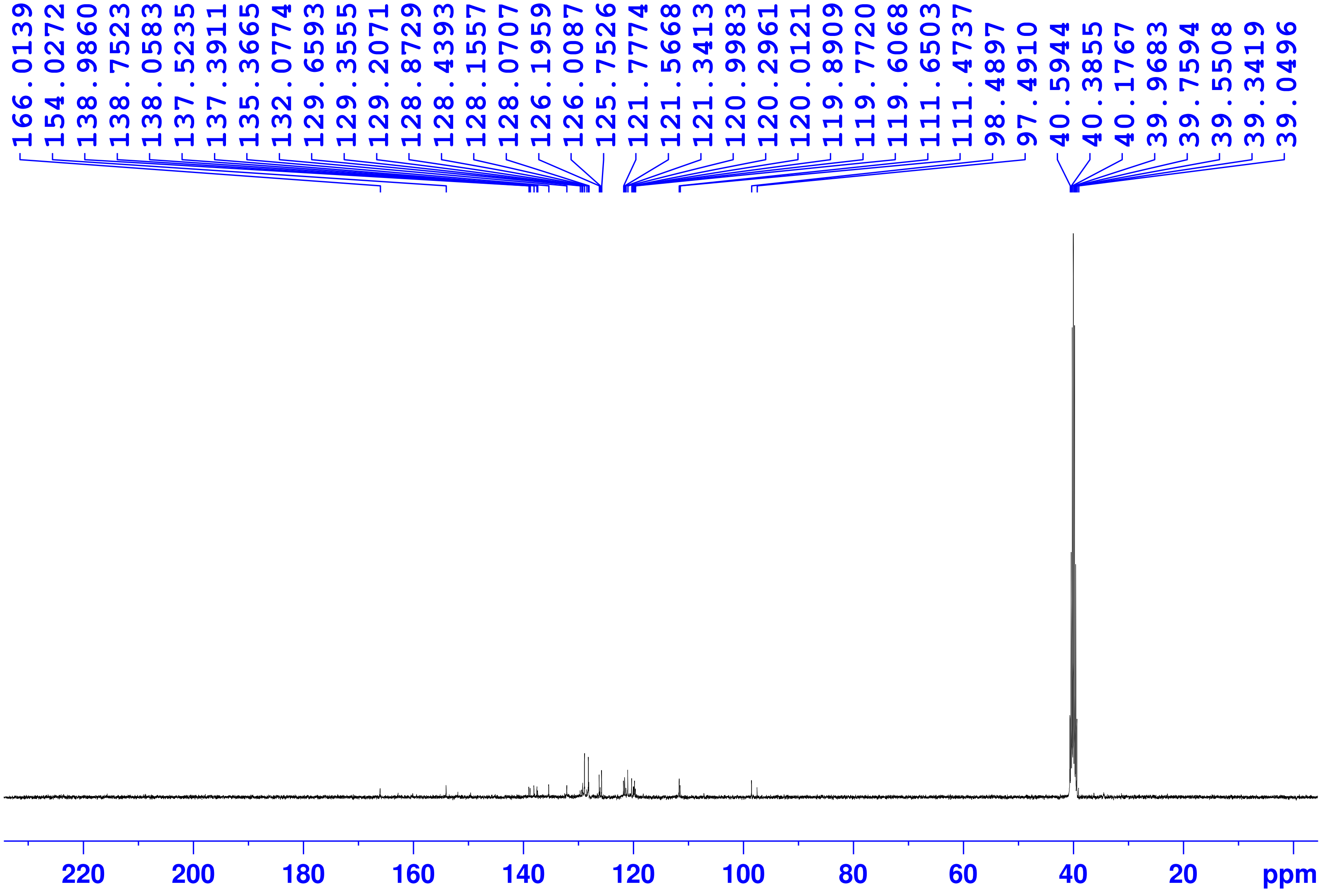

Current Data Parameters

NAME	Dr. Humaira Nadeem Riphah
EXPNO	2
PROCNO	1

F2 - Acquisition Parameters

Date_	20240611
Time	3.00 h
INSTRUM	spect
PROBHD	Z116098_0621 (
PULPROG	zgpg30
TD	65536
SOLVENT	DMSO
NS	400
DS	2
SWH	24038.461 Hz
FIDRES	0.733596 Hz
AQ	1.3631488 sec
RG	199.48
DW	20.800 usec
DE	6.50 usec
TE	298.0 K
D1	2.00000000 sec
D11	0.03000000 sec
TD0	1
SFO1	100.6243390 MHz
NUC1	13C
P1	10.00 usec
PLW1	72.56700134 W
SFO2	400.1316005 MHz
NUC2	1H
CPDPRG[2	waltz16
PCPD2	90.00 usec
PLW2	16.68099976 W
PLW12	0.20593999 W
PLW13	0.10342000 W

F2 - Processing parameters

SI	32768
SF	100.6127685 MHz
WDW	EM
SSB	0
LB	1.00 Hz
GB	0
PC	1.40

4-API-B/DMSO-d6/Adil Saeed/Dr. Humaira Nadeem/Riphah Univ

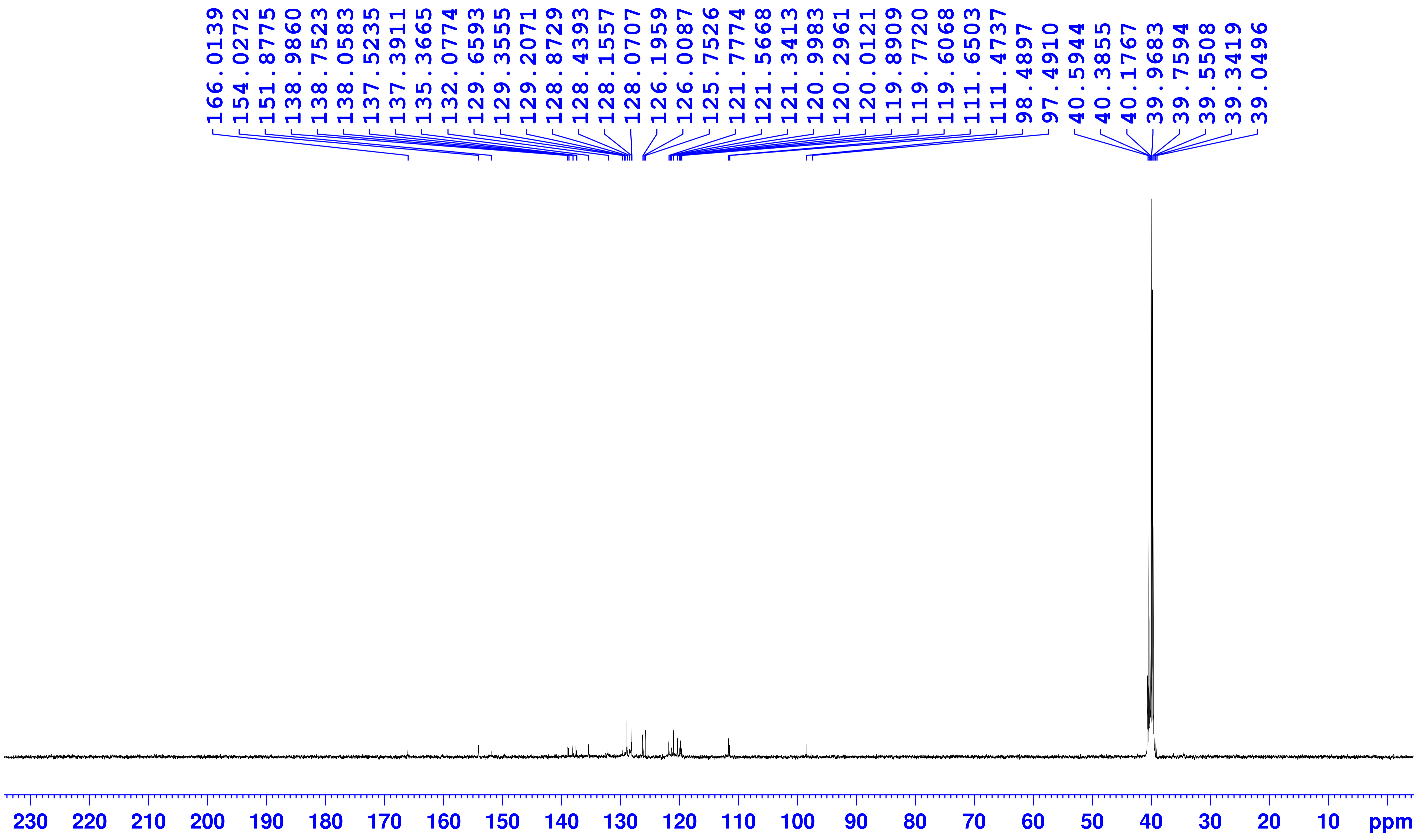

4-API-B/DMSO-d6/Adil Saeed/Dr. Humaira Nadeem/Riphah Univ

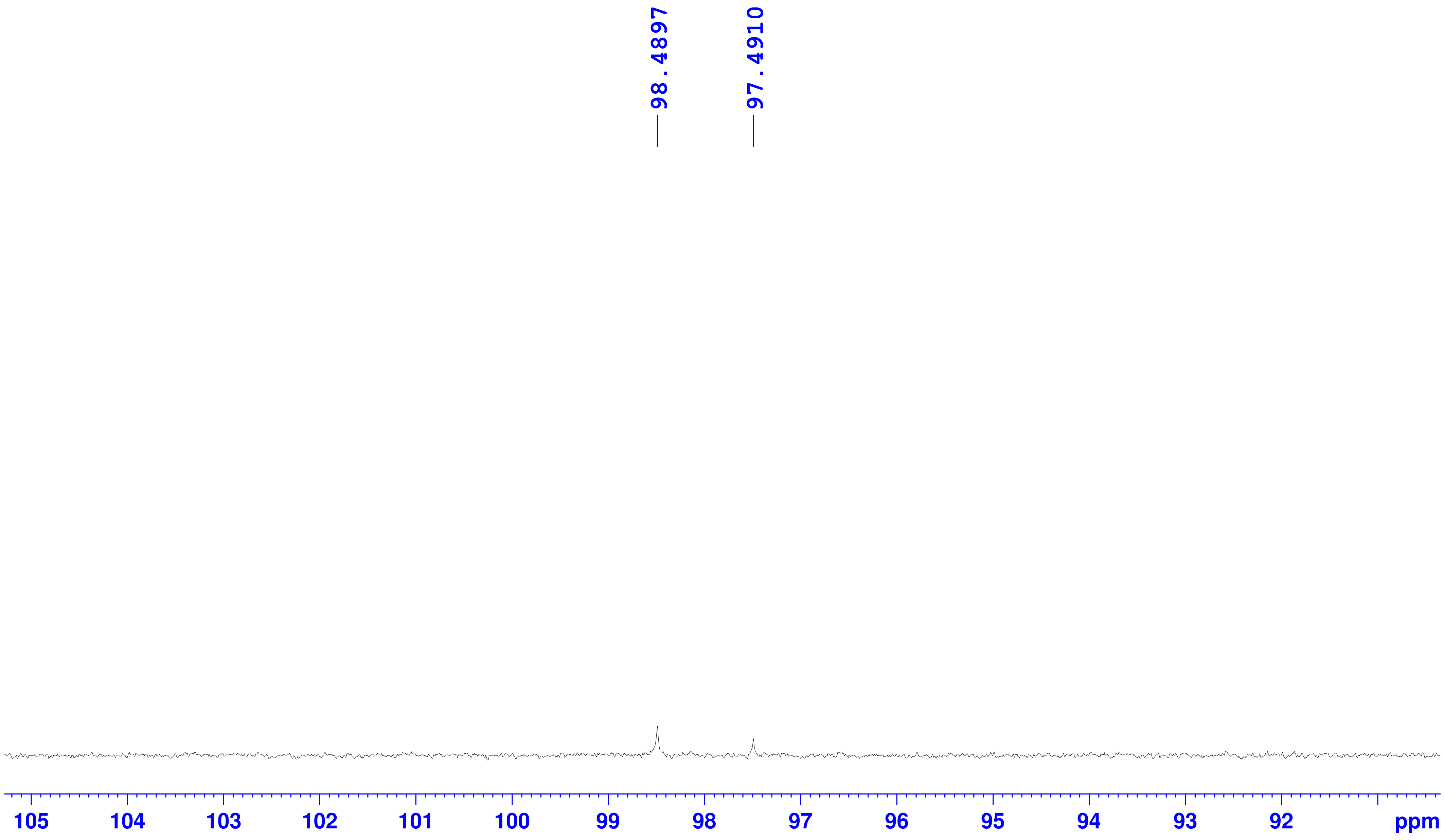

4-API-B/DMSO-d6/Adil Saeed/Dr. Humaira Nadeem/Riphah Univ

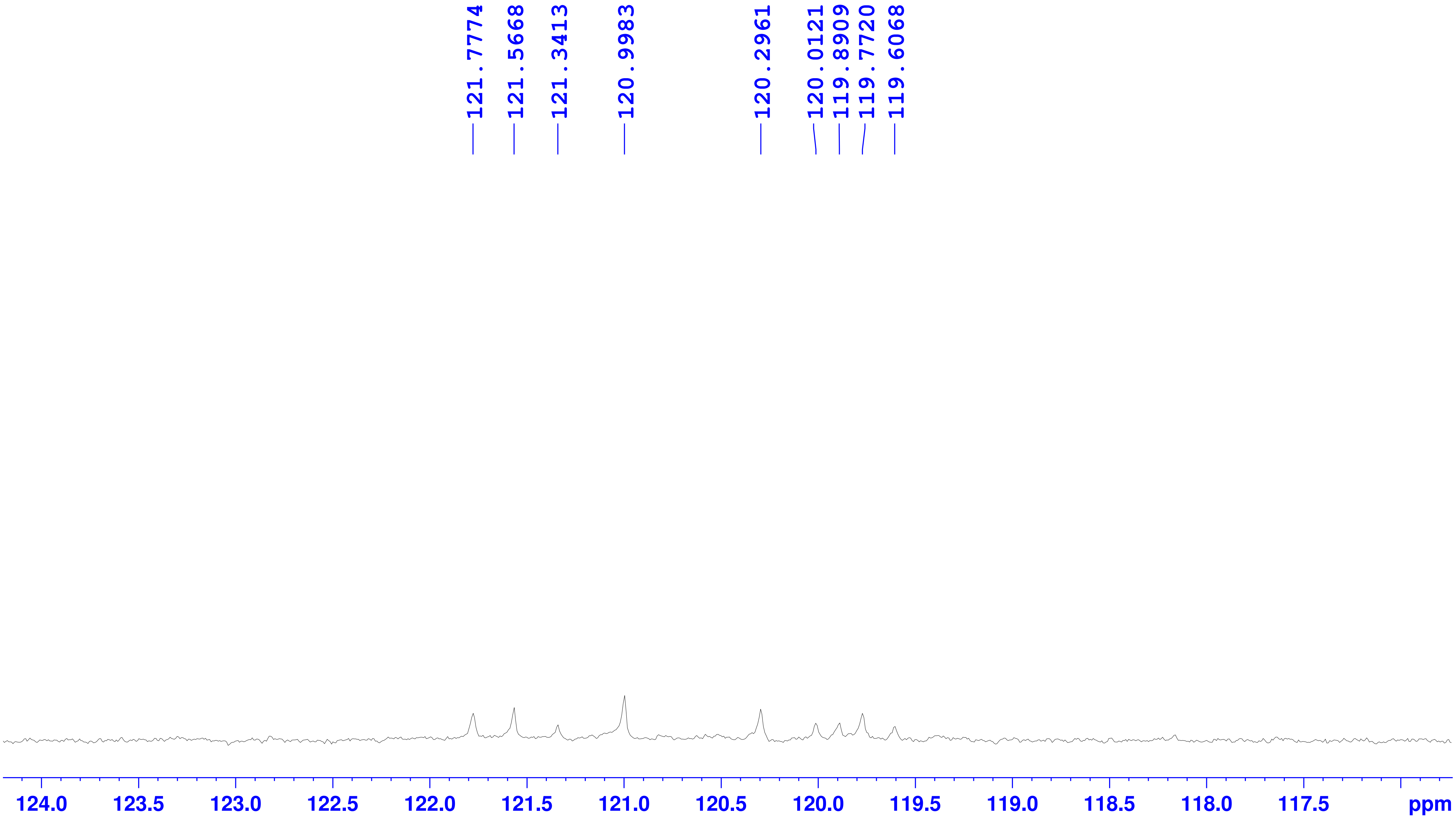

4-API-B/DMSO-d6/Adil Saeed/Dr. Humaira Nadeem/Riphah Univ

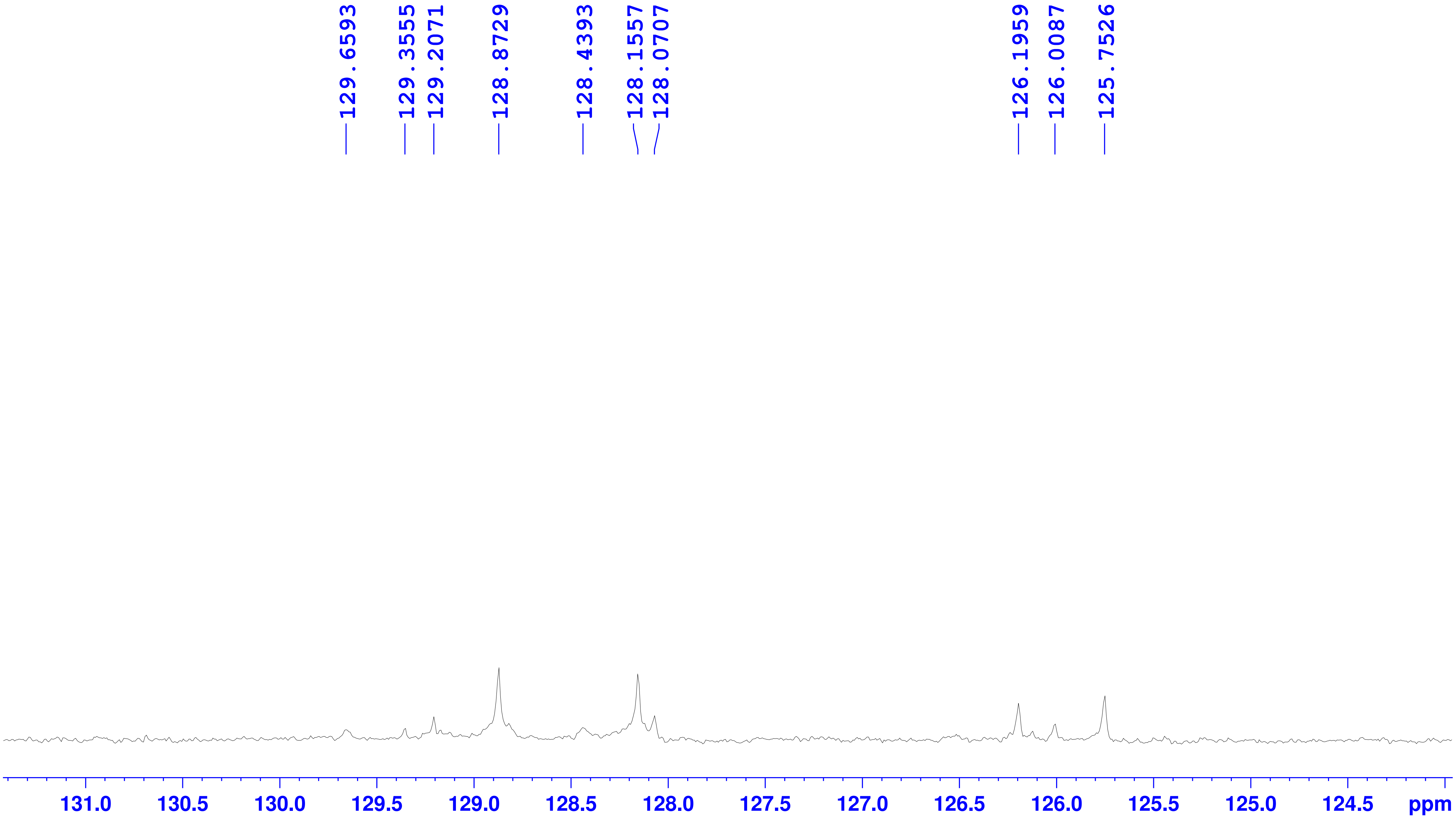

4-API-B/DMSO-d6/Adil Saeed/Dr. Humaira Nadeem/Riphah Univ

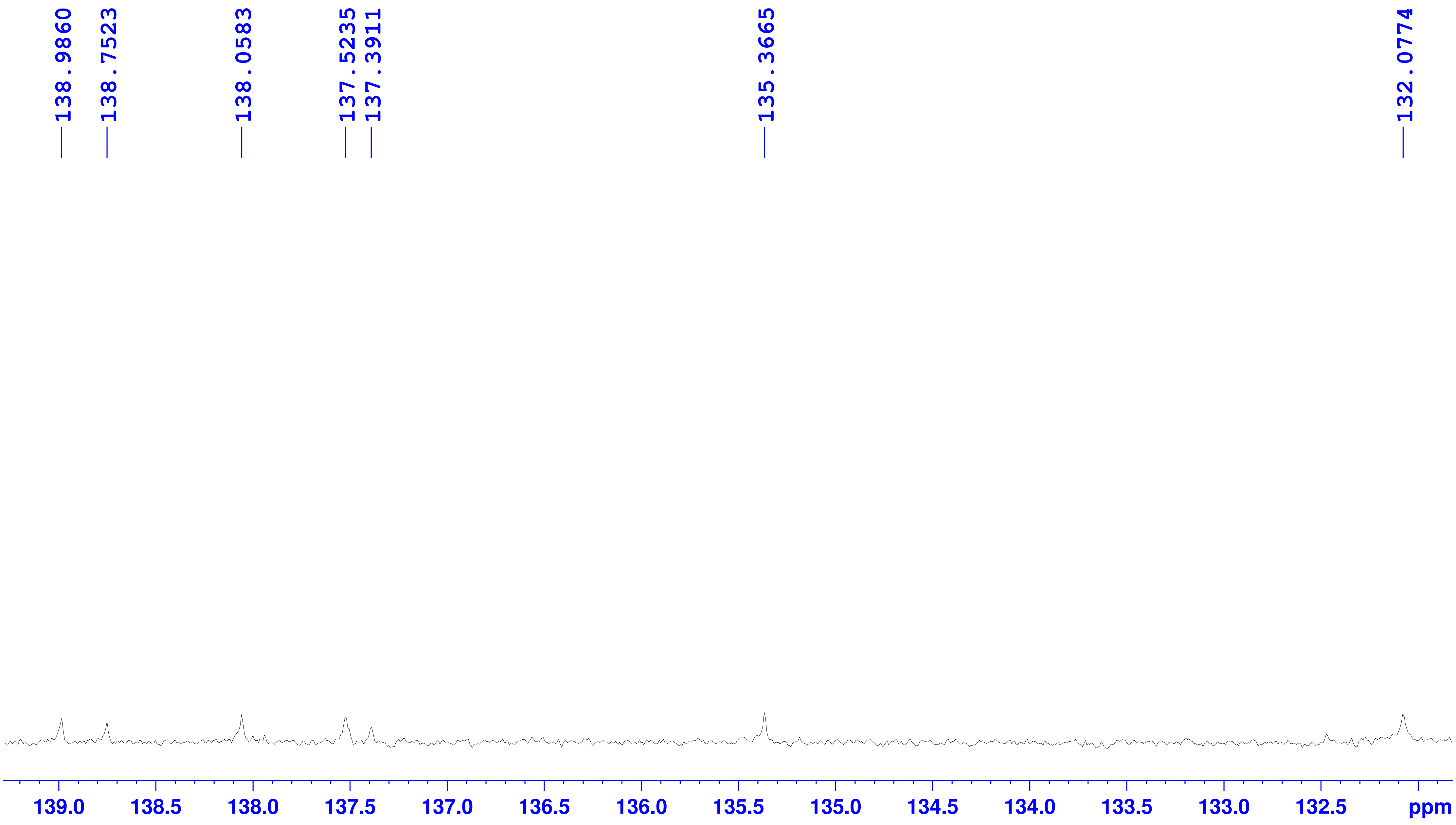

4-API-B/DMSO-d6/Adil Saeed/Dr. Humaira Nadeem/Riphah Univ

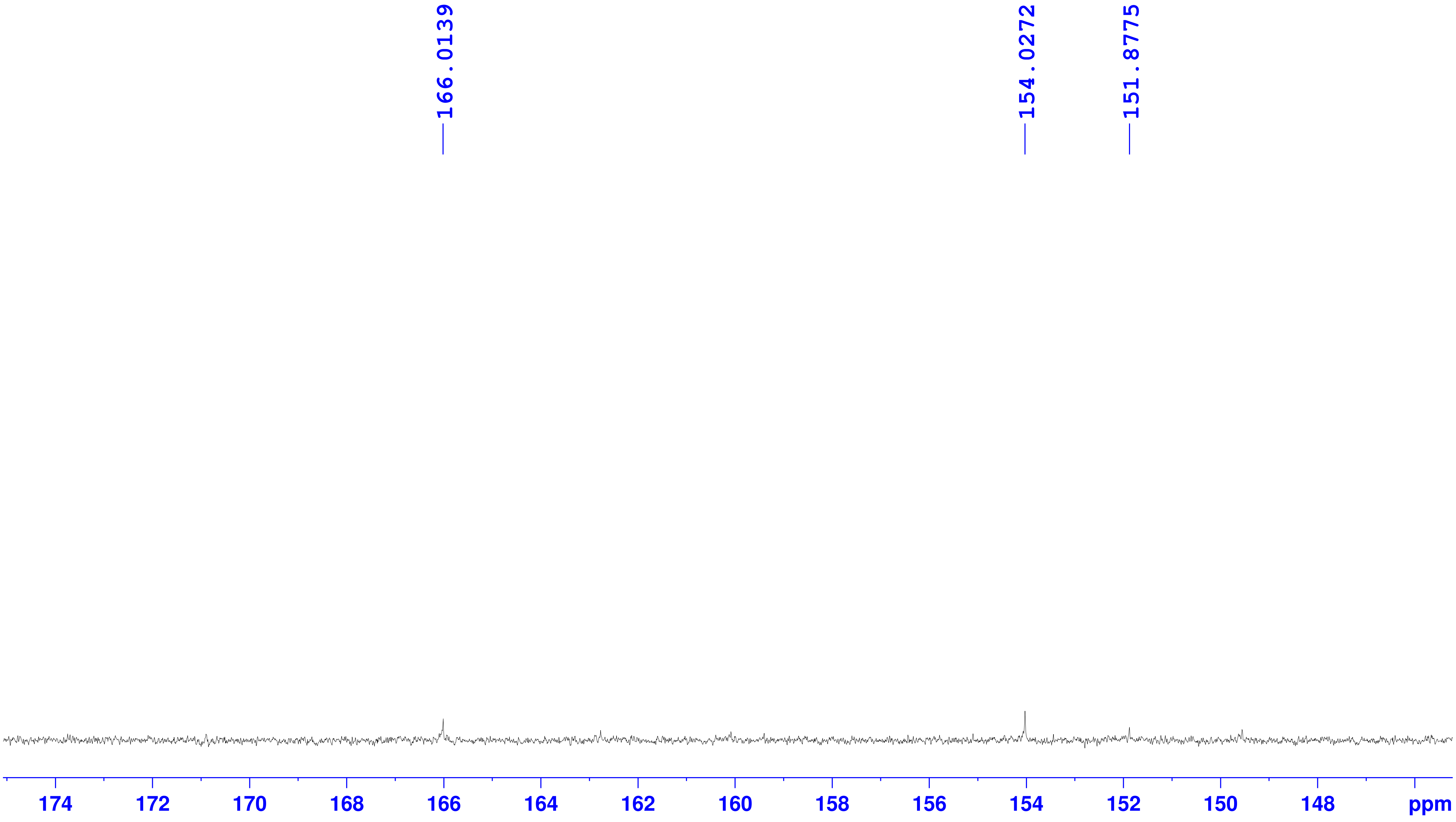

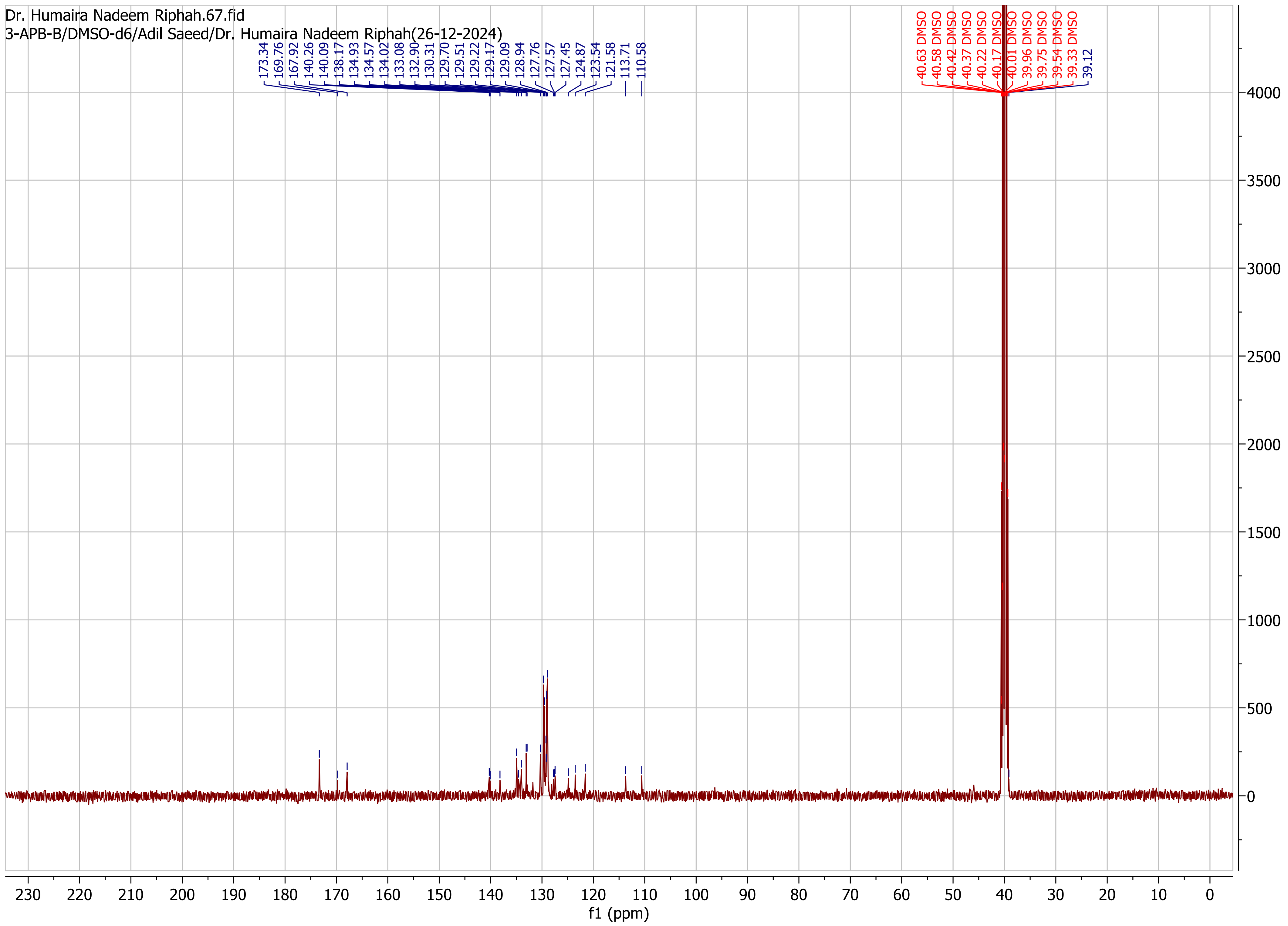
Dr. Humaira Nadeem Riphah.67.fid
3-APB-B/DMSO-d6/Adil Saeed/Dr. Humaira Nadeem Riphah(26-12-2024)
173.34
169.76
167.92
140.26
140.09
138.17
134.93
134.57
134.02
133.08
132.90
130.31
129.70
129.51
129.22
129.17
129.09
128.94
127.76
127.57
127.45
124.87
123.54
121.58
113.71
110.58
40.63 DMSO
40.58 DMSO
40.42 DMSO
40.37 DMSO
40.22 DMSO
40.17 DMSO
40.01 DMSO
39.96 DMSO
39.75 DMSO
39.54 DMSO
39.33 DMSO
39.12
f1 (ppm)

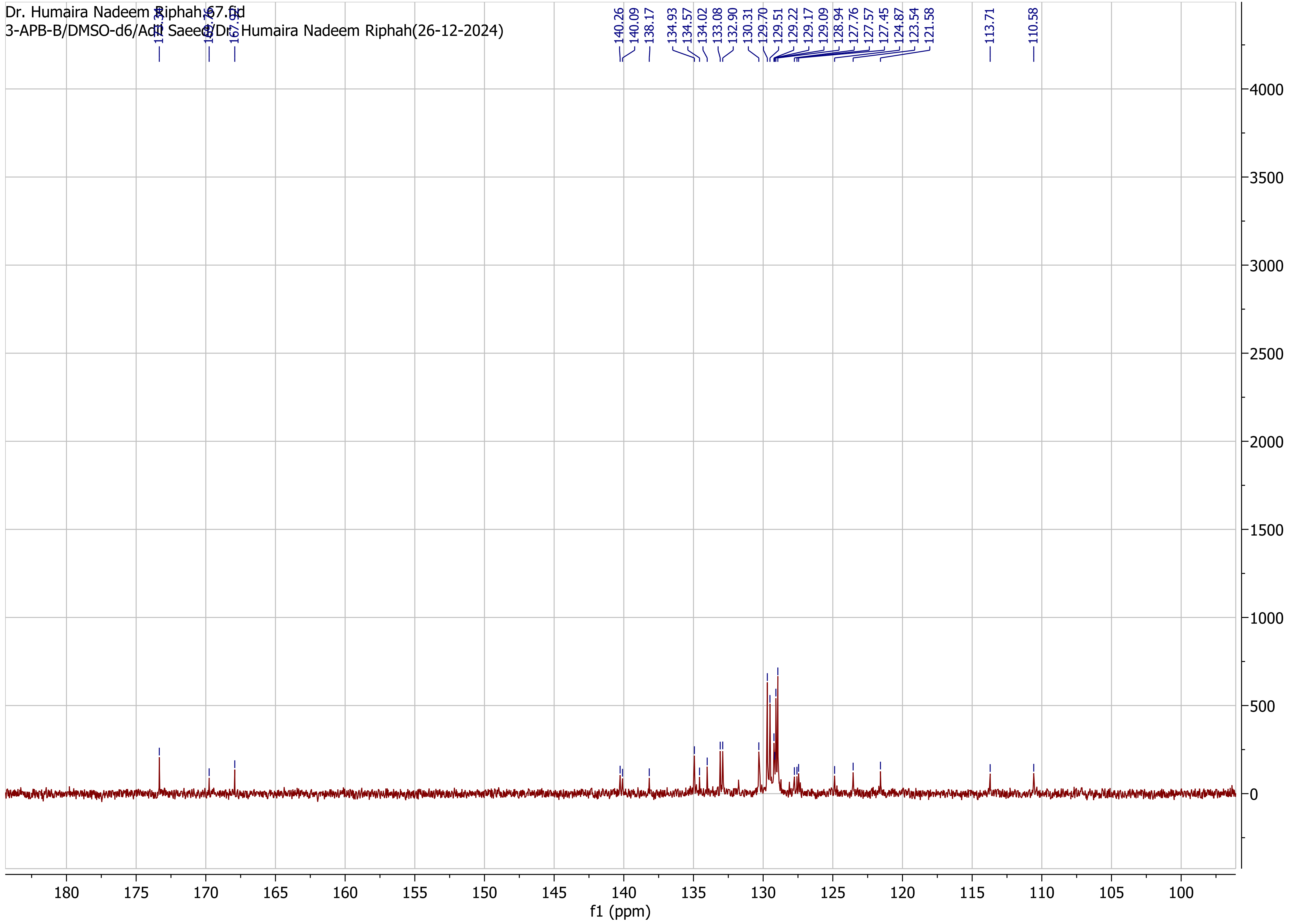

Dr. Humaira Nadeem Riphah.67.fid
3-APB-B/DMSO-d6/Adil Saeed/Dr. Humaira Nadeem Riphah(26-12-2024)
173.34
169.76
167.92
140.26
140.09
138.17
134.93
134.57
134.02
133.08
132.90
130.31
129.70
129.51
129.22
129.17
129.09
128.94
127.76
127.57
127.45
124.87
123.54
121.58
113.71
110.58
4000
3500
3000
2500
2000
1500
1000
500
0
180
175
170
165
160
155
150
145
140
135
130
125
120
115
110
105
100
f1 (ppm)

Dr. Humaira Nadeem Riphah.66.fid
3-APB-B/DMSO-d6/Adil Saeed/Dr. Humaira Nadeem Riphah(26-12-2024)

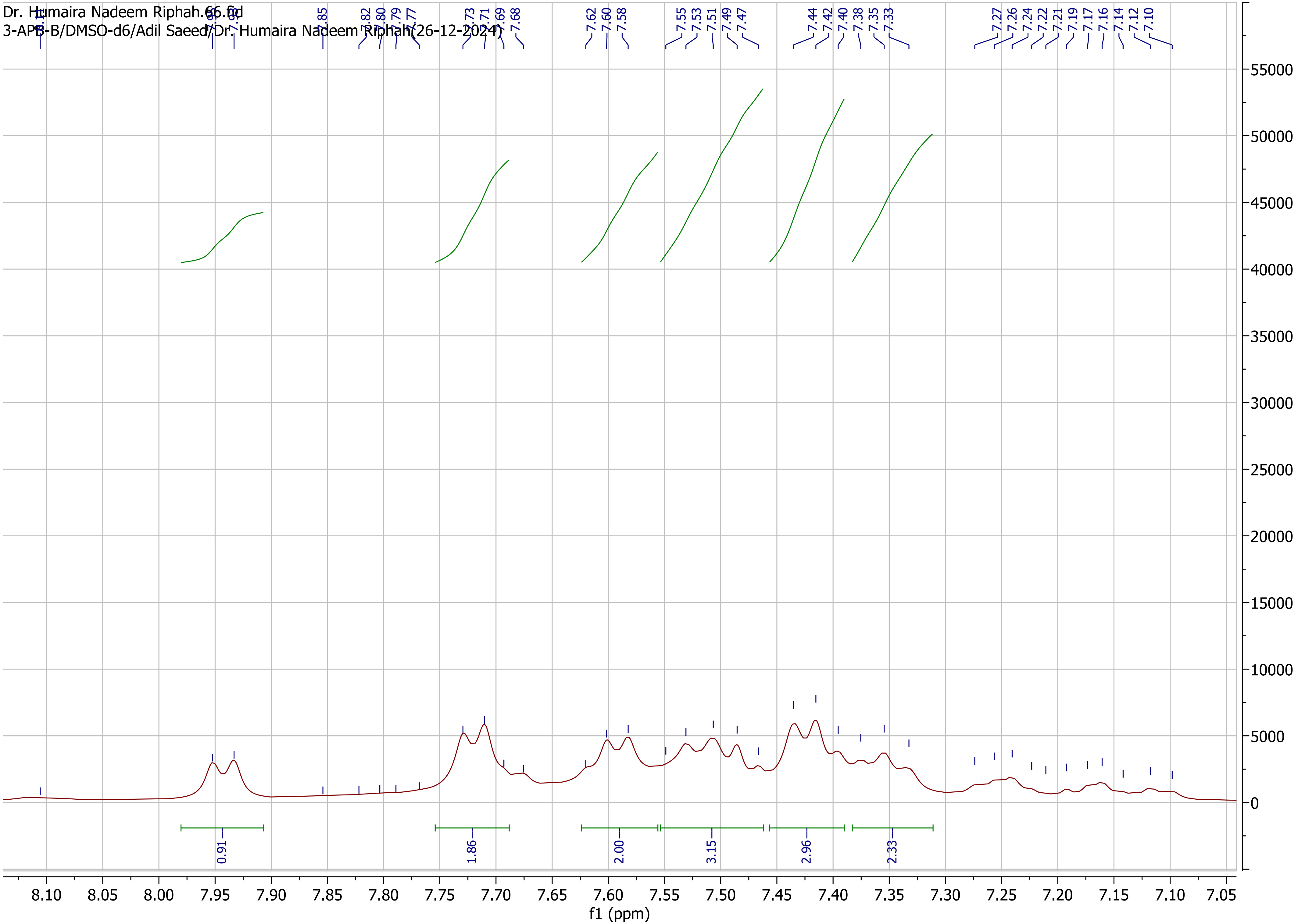

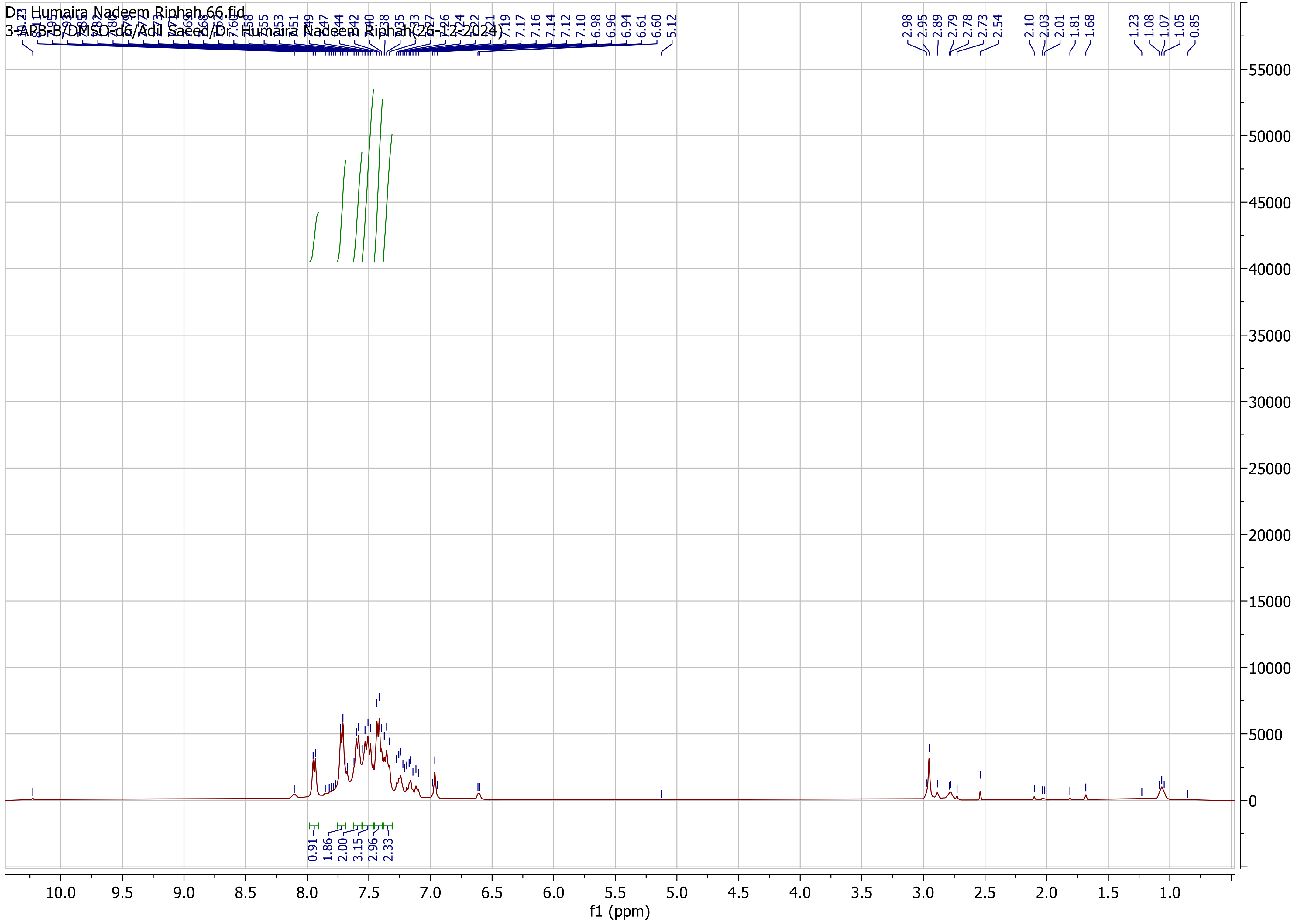

Dr. Humaira Nadeem Riphah.66.fid
3-APB-B/DMSO-d6/Adil Saeed/Dr. Humaira Nadeem Riphah(26-12-2024)
7.19
7.17
7.16
7.14
7.12
7.10
6.98
6.96
6.94
6.61
6.60
5.12
2.98
2.95
2.89
2.79
2.78
2.73
2.54
2.10
2.03
2.01
1.81
1.68
1.23
1.08
1.07
1.05
0.85
0.91
1.86
2.00
3.15
2.96
2.33
f1 (ppm)

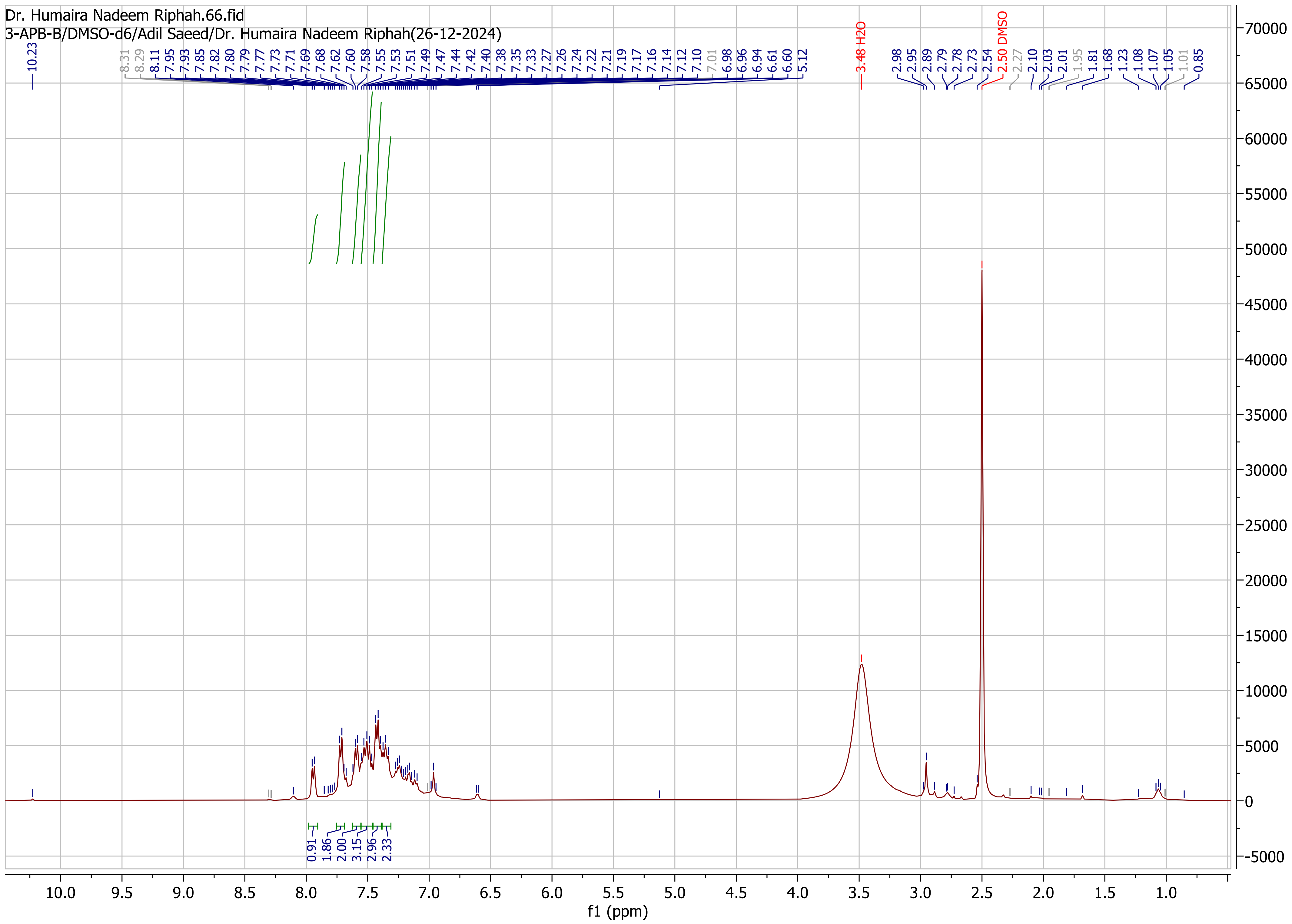

Dr. Humaira Nadeem Riphah.66.fid
3-APB-B/DMSO-d6/Adil Saeed/Dr. Humaira Nadeem Riphah(26-12-2024)
3.48 H2O
2.50 DMSO
f1 (ppm)

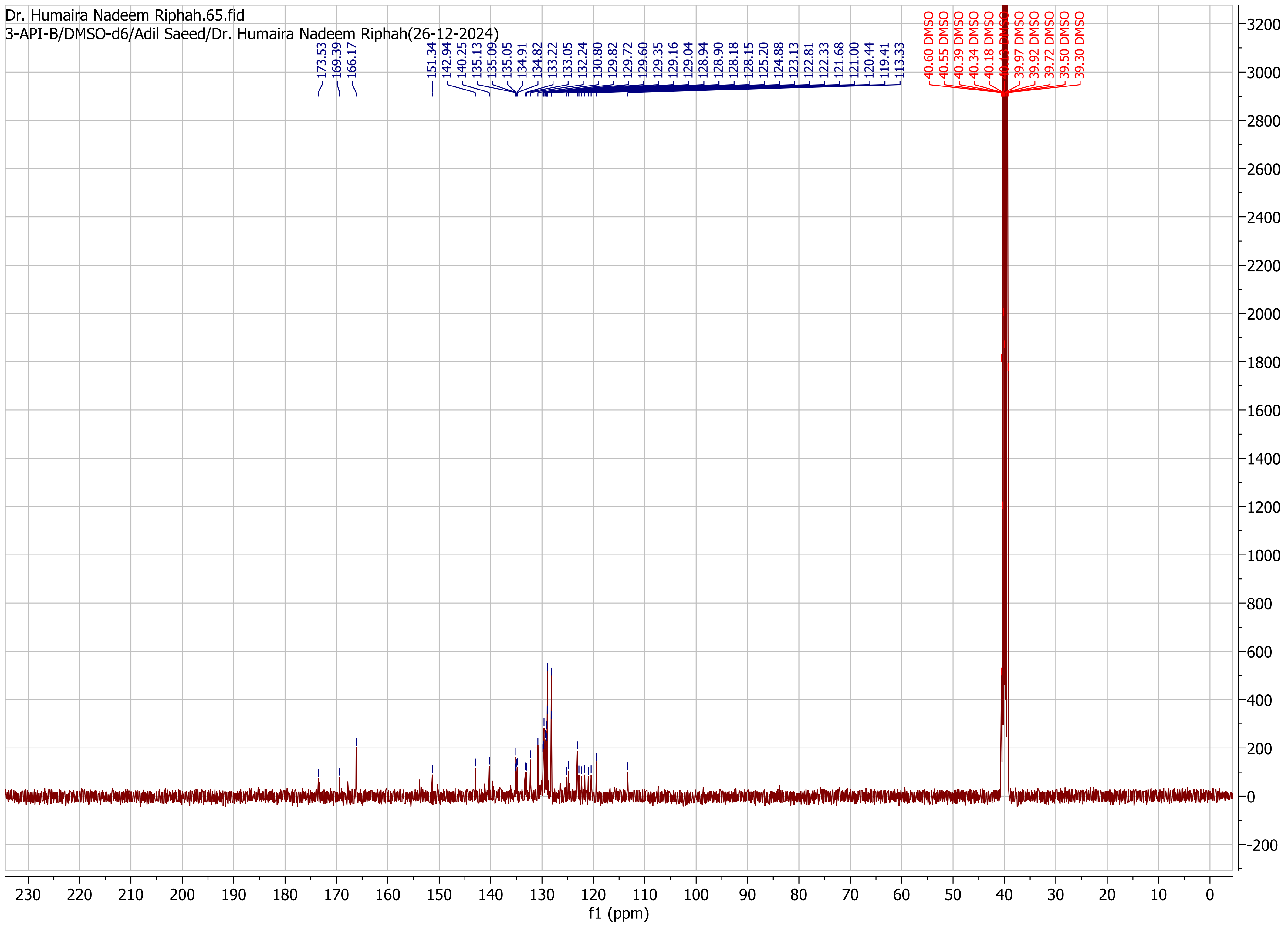

Dr. Humaira Nadeem Riphah.65.fid
3-API-B/DMSO-d6/Adil Saeed/Dr. Humaira Nadeem Riphah(26-12-2024)
173.53
169.39
166.17
151.34
142.94
140.25
135.13
135.09
135.05
134.91
134.82
133.22
133.05
132.24
130.80
129.82
129.72
129.60
129.35
129.16
129.04
128.94
128.90
128.18
128.15
125.20
124.88
123.13
122.81
122.33
121.68
121.00
120.44
119.41
113.33
40.60 DMSO
40.55 DMSO
40.39 DMSO
40.34 DMSO
40.18 DMSO
39.97 DMSO
39.92 DMSO
39.72 DMSO
39.50 DMSO
39.30 DMSO
f1 (ppm)

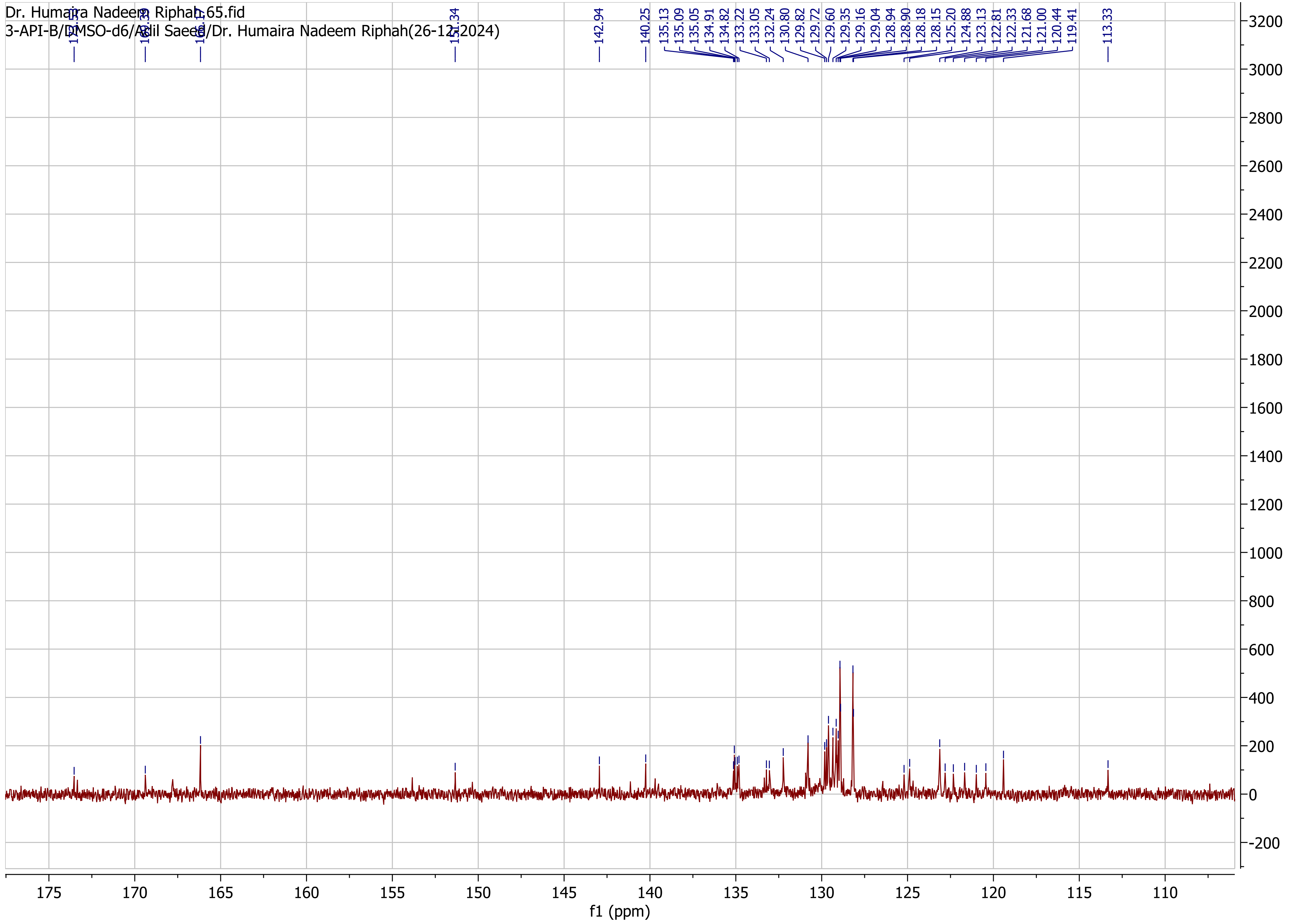
Dr. Humaira Nadeem Riphah.65.fid
3-API-B/DMSO-d6/Adil Saeed/Dr. Humaira Nadeem Riphah(26-12-2024)
151.34
142.94
140.25
135.13
135.09
135.05
134.91
134.82
133.22
133.05
132.24
130.80
129.82
129.72
129.60
129.35
129.16
129.04
128.94
128.90
128.18
128.15
125.20
124.88
123.13
122.81
122.33
121.68
121.00
120.44
119.41
113.33
f1 (ppm)

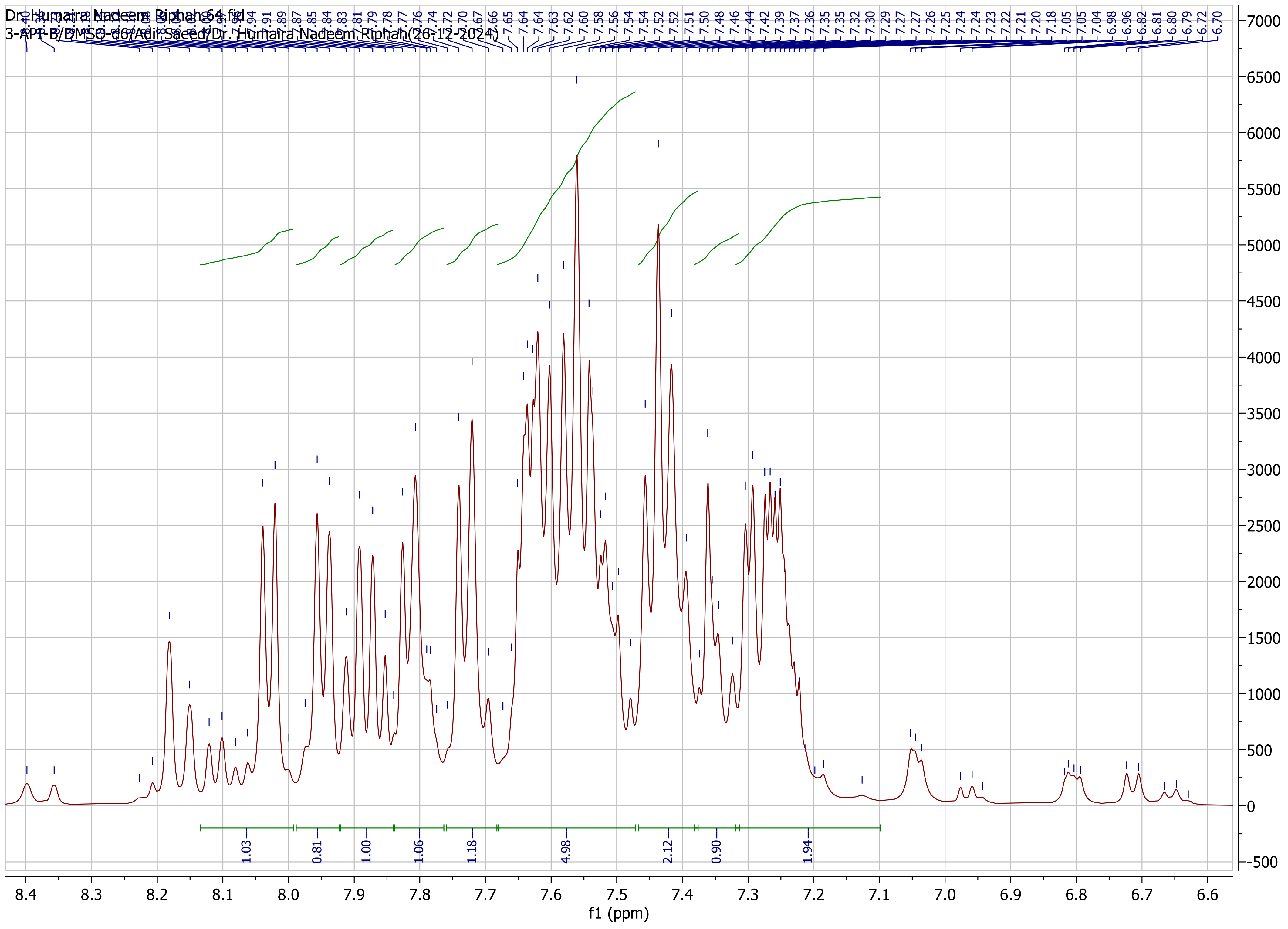
Dr. Humaira Nadeem Riphah 64.fid
3-APT-B/DMSO-d6/Adil Saeed/Dr. Humaira Nadeem Riphah(26-12-2024)
1.03
0.81
1.00
1.06
1.18
4.98
2.12
0.90
1.94
f1 (ppm)

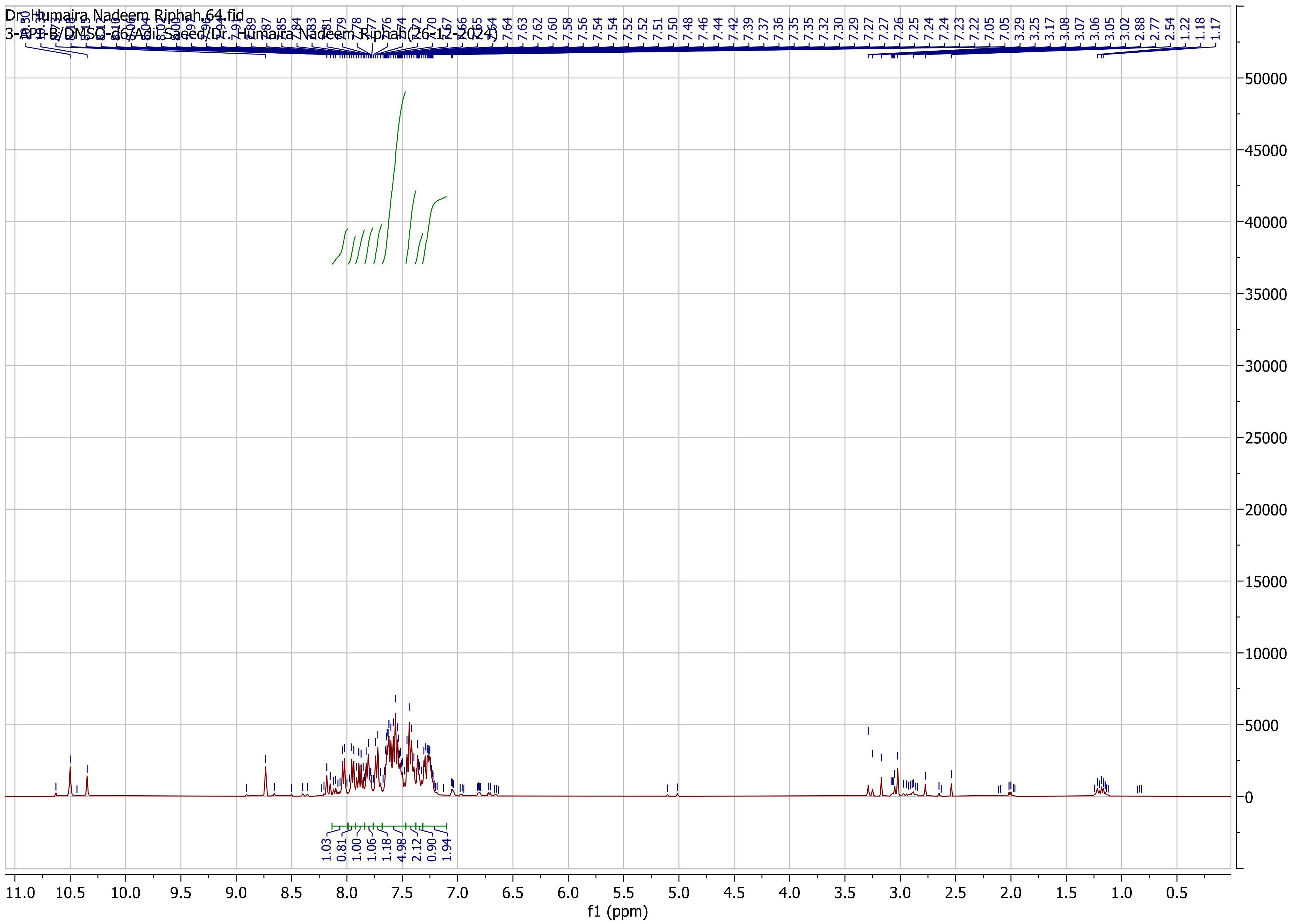
Dr Humaira Nadeem Riphah.64.fid
3-AP1-B/DMSO-d6/Adil Saeed/Dr. Humaira Nadeem Riphah(26-12-2024)
7.64 7.63 7.62 7.60 7.58 7.56 7.54 7.54 7.52 7.52 7.51 7.50 7.48 7.46 7.44 7.42 7.39 7.37 7.36 7.35 7.35 7.32 7.30 7.29 7.27 7.27 7.26 7.25 7.24 7.24 7.23 7.22 7.05 7.05 3.29 3.25 3.17 3.08 3.07 3.06 3.05 3.02 2.88 2.77 2.54 1.22 1.18 1.17
1.03 0.81 1.00 1.06 1.18 4.98 2.12 0.90 1.94
f1 (ppm)

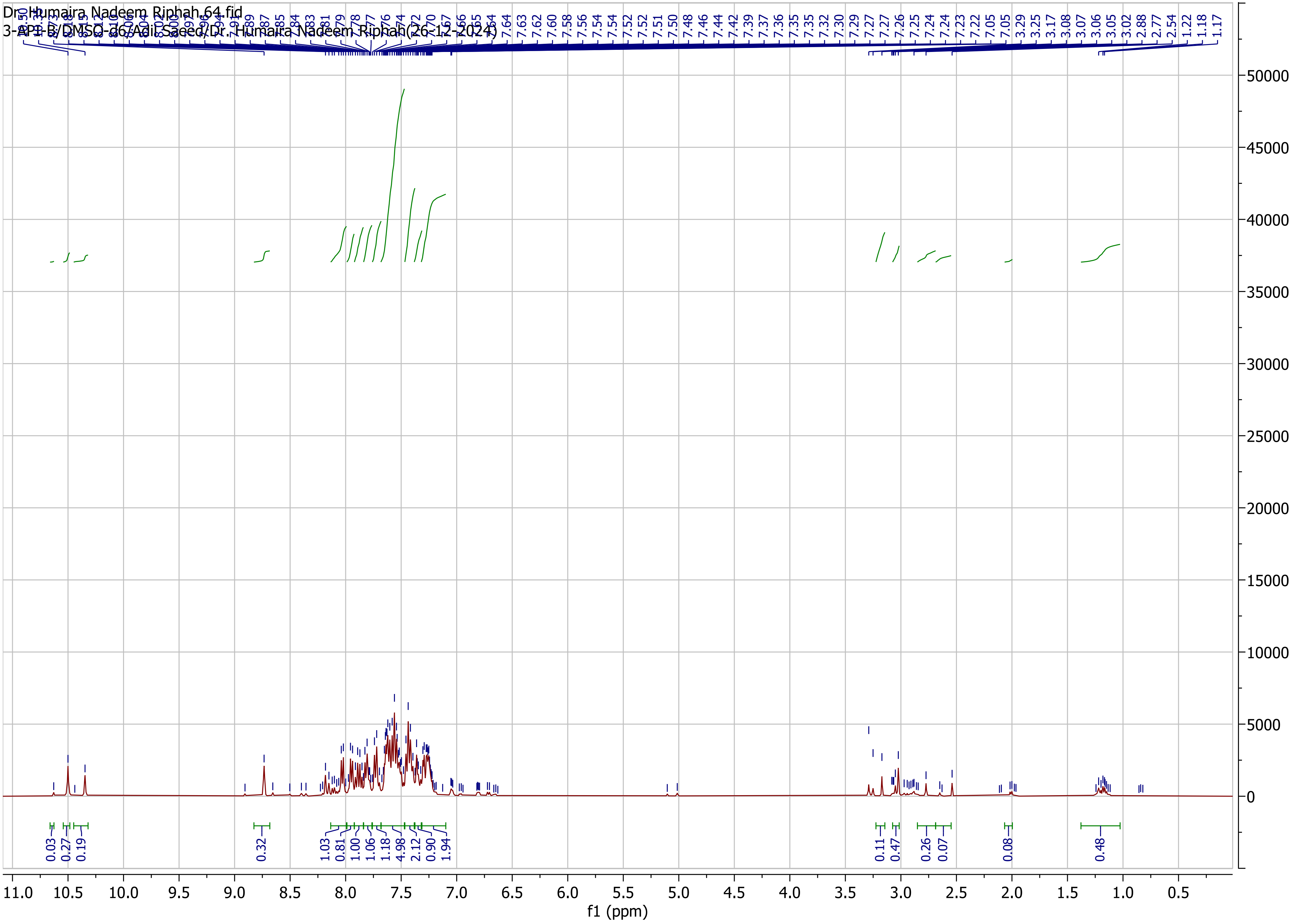

Dr Humaira Nadeem Riphah.64.fid
3-AP1-B/DMSO-d6/Adil Saeed/Dr. Humaira Nadeem Riphah(26-12-2024)
7.64
7.63
7.62
7.60
7.58
7.56
7.54
7.54
7.52
7.52
7.51
7.50
7.48
7.46
7.44
7.42
7.39
7.37
7.36
7.35
7.35
7.32
7.30
7.29
7.27
7.27
7.26
7.25
7.24
7.24
7.23
7.22
7.05
7.05
3.29
3.25
3.17
3.08
3.07
3.06
3.05
3.02
2.88
2.77
2.54
1.22
1.18
1.17
0.03
0.27
0.19
0.32
1.03
0.81
1.00
1.06
1.18
4.98
2.12
0.90
1.94
0.11
0.47
0.26
0.07
0.08
0.48
11.0
10.5
10.0
9.5
9.0
8.5
8.0
7.5
7.0
6.5
6.0
5.5
5.0
4.5
4.0
3.5
3.0
2.5
2.0
1.5
1.0
0.5
f1 (ppm)
50000
45000
40000
35000
30000
25000
20000
15000
10000
5000
0

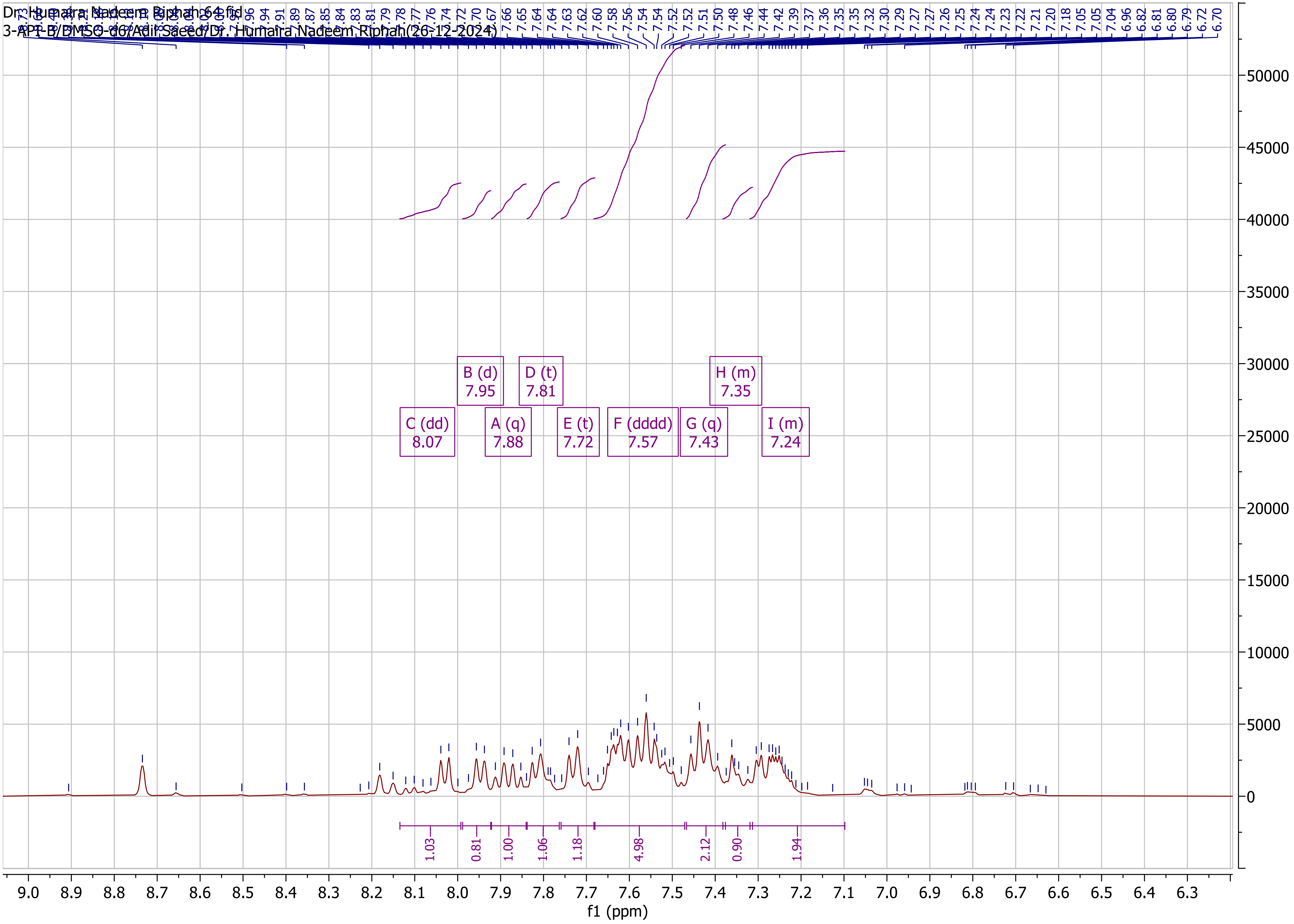

Dr. Humaira Nadeem Riphah 64 fid
3-APT-B/DMSO-d6/Adil Saeed/Dr. Humaira Nadeem Riphah(26-12-2024)
B (d) 7.95
D (t) 7.81
H (m) 7.35
C (dd) 8.07
A (q) 7.88
E (t) 7.72
F (dddd) 7.57
G (q) 7.43
I (m) 7.24
1.03
0.81
1.00
1.06
1.18
4.98
2.12
0.90
1.94
f1 (ppm)
